# MiMiR – an integrated platform for microarray data sharing, mining and analysis

**DOI:** 10.1186/1471-2105-9-379

**Published:** 2008-09-18

**Authors:** Chris Tomlinson, Manjula Thimma, Stelios Alexandrakis, Tito Castillo, Jayne L Dennis, Anthony Brooks, Thomas Bradley, Carly Turnbull, Ekaterini Blaveri, Geraint Barton, Norie Chiba, Klio Maratou, Pat Soutter, Tim Aitman, Laurence Game

**Affiliations:** 1 Microarray Centre, MRC Clinical Sciences Centre and Imperial College, Hammersmith Hospital, Du Cane Road, London, W12 0NN, UK; 2 Physiological Genomics and Medicine Group, MRC Clinical Sciences Centre, Faculty of Medicine, Imperial College, Hammersmith Hospital, Du Cane Road, London, W12 0NN, UK; 3 Health Dialog, Wellington House, East Road, Cambridge, CB1 1BH, UK; 4 NCRI Informatics Initiative, 61 Lincoln's Inn Fields, London, WC2A 3PX, UK; 5 Centre for Integrated Systems Biology at Imperial College, Imperial College, South Kensington Campus, London, SW7 2AZ, UK; 6 Bioinformatics Support Service, Imperial College, South Kensington Campus, London, SW7 2AZ, UK; 7 Imperial College, Hammersmith Hospital, Du Cane Road, London, W12 0NN, UK

## Abstract

**Background:**

Despite considerable efforts within the microarray community for standardising data format, content and description, microarray technologies present major challenges in managing, sharing, analysing and re-using the large amount of data generated locally or internationally. Additionally, it is recognised that inconsistent and low quality experimental annotation in public data repositories significantly compromises the re-use of microarray data for meta-analysis. MiMiR, the **Mi**croarray data **Mi**ning **R**esource was designed to tackle some of these limitations and challenges. Here we present new software components and enhancements to the original infrastructure that increase accessibility, utility and opportunities for large scale mining of experimental and clinical data.

**Results:**

A user friendly Online Annotation Tool allows researchers to submit detailed experimental information via the web at the time of data generation rather than at the time of publication. This ensures the easy access and high accuracy of meta-data collected. Experiments are programmatically built in the MiMiR database from the submitted information and details are systematically curated and further annotated by a team of trained annotators using a new Curation and Annotation Tool. Clinical information can be annotated and coded with a clinical Data Mapping Tool within an appropriate ethical framework. Users can visualise experimental annotation, assess data quality, download and share data via a web-based experiment browser called MiMiR Online. All requests to access data in MiMiR are routed through a sophisticated middleware security layer thereby allowing secure data access and sharing amongst MiMiR registered users prior to publication. Data in MiMiR can be mined and analysed using the integrated EMAAS open source analysis web portal or via export of data and meta-data into Rosetta Resolver data analysis package.

**Conclusion:**

The new MiMiR suite of software enables systematic and effective capture of extensive experimental and clinical information with the highest MIAME score, and secure data sharing prior to publication. MiMiR currently contains more than 150 experiments corresponding to over 3000 hybridisations and supports the Microarray Centre's large microarray user community and two international consortia. The MiMiR flexible and scalable hardware and software architecture enables secure warehousing of thousands of datasets, including clinical studies, from microarray and potentially other -omics technologies.

## Background

Microarray technologies have matured rapidly over the past few years and present major challenges in managing, sharing, analysing and re-using the large amount of data generated [1] despite the considerable international efforts in standardising data format, content and description [2-6]. Vast numbers of microarray experiments are performed worldwide every year, many of which become available upon publication via public repositories like Gene Expression Omnibus [7] and ArrayExpress [8]. Many microarray databases have been created to support local communities with various focuses, for example on species [9] and  microarray platform [10,11], disease [12-14], institutions or research projects [15-18].

There are two major limitations of public repositories and most microarray databases. First, although most microarray databases are 'MIAME-compliant', i.e. are designed to capture the MIAME [5] minimal experimental information, these standards and guidelines are often not enforced, leading to variable, often very minimal, levels of experimental detail stored alongside microarray data. Because researchers who submit data to public repositories are ultimately responsible for the completeness, quality and accuracy of their submission [7], the majority of data sets in public repositories have insufficient experimental information available in order for the data to be re-used effectively in a different analysis. A recent study looking at Affymetrix data in GEO and ArrayExpress identified that only 38% of the microarray data meets the quality and format standards necessary for further integrative analysis [1]. Second, the absence of appropriate security models in public repositories and many microarray databases makes it difficult or sometimes impossible to share data online prior to publication or to securely store sensitive biological or clinical information that would be important for meta-analysis. In addition, the effective collection, annotation and mining of detailed information on clinical samples e.g. patient age at diagnosis, detailed disease and treatment information, clinical treatment follow up and outcome data, is particularly challenging due to legal restrictions associated with storing and disclosing patient and volunteer clinical information (even in an anonymised way).

MiMiR, the **Mi**croarray Data **Mi**ning **R**esource is an integrated platform for microarray data sharing, mining and analysis that addresses many of these limitations. MiMiR stores experimental information to a level of detail higher than that suggested by MIAME using ontologies and naming conventions [19]. It provides a powerful platform for large scale data mining and analysis and enables deposition of data in ArrayExpress on publication. MiMiR was initially developed to be used within the Microarray Centre and was not directly accessible to researchers [19]. Here we describe new software components and enhancements of the original infrastructure that allow researchers to securely submit, access, share and analyse microarray and meta-data. Specifically, we have created: (i) a re-engineered hardware and software architecture that protects the MiMiR database integrity and enables secure online sharing of unpublished and public data amongst registered users; (ii) a new web based annotation tool allowing researchers to easily and quickly submit information about their experiments and samples; (iii) new sophisticated curation and annotation tools which automatically create annotated experiments in MiMiR and enable in-house annotators to check it and add ontology terms and systematic naming conventions; (iv) a clinical Data Mapping Tool to securely capture clinical information in a systematic way within an appropriate ethical framework; (v) a new user-friendly web interface that is used by researchers to visualise extensive experimental annotation, to download data and quality assessment reports and to share un-published datasets with collaborators or other registered users of the system; (vi) a re-engineered MAGE-ML pipeline for exporting experiments from MiMiR into the ArrayExpress repository or into the Rosetta Resolver package for data analysis; (vii) programmatic access to MiMiR from the new open source microarray data analysis software package EMAAS [20], allowing users to export selected data and associated meta-data for analysis.

## Construction and content

### • MiMiR security model and software architecture

**Figure 1:**
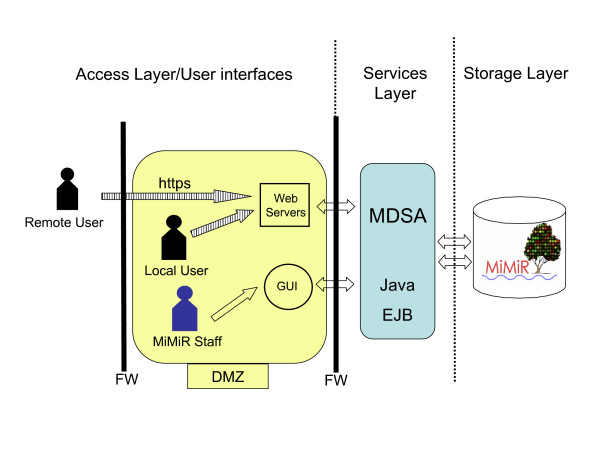
**The three-tier hardware and software architecture of MiMiR comprises a storage layer, an application services layer and an access/user interface layer (delimited by dashed lines).** The MiMiR backend database is physically protected by two firewalls (FW) and is only accessible through the middle tier application servers (MDSA) which act as trusted middleware service and security gateway. The demilitarised zone (DMZ) sits between the two firewalls. All requests and retrieval of data to and from the web servers in the access layer are done in an encrypted format (marked 'https' with shaded arrows). MiMiR staff access internal tools for experimental annotation (GUI) using a less secure data transmission.

In order to support the growing volume of stored data, the efficient mining capabilities and secure data access by multiple concurrent users, we modified the original MiMiR infrastructure and database schema [19]. A three-tier architecture comprising a data storage layer, an application services layer and a user interface layer was designed and implemented (Figure 1). This layered approach decouples (i.e. reduces the dependency of) the various software components from the MiMiR database which ensures high scalability and a flexible environment for software and applications development. The web and any other user-interface layer application servers are located in the de-militarised zone (DMZ) [21] which is protected by one firewall. A sophisticated middleware layer framework, called MiMiR Data Services Architecture (MDSA), was developed using Enterprise JavaBeans (EJB) to allow highly secure remote access to the data in MiMiR via a role/permissions-based security model. A list of registered users and role-specific permissions is maintained in the database to identify and grant access rights. All the client applications are accessible to registered users upon login with username and password. Clinical information stored in MiMiR can also be filtered according to ethical policies before being delivered back to the client or written into the clinical part of MiMiR.

### • Experimental information capture, curation and annotation

MiMiR stores a high level of experimental information which exceeds that required by the MIAME guidelines [19]. The experimental annotation process was enhanced by implementing an online data collection tool to allow users to easily and quickly submit, via the web, detailed experimental information. An internal curation and annotation tool automatically constructs an experimental model in MiMiR based on information provided, which can be checked and further annotated by trained staff.

#### MiMiR online experimental data collection

Experimental information is collected from users at the time of data generation rather than at the time of publication. This ensures easy recall, access and high accuracy of the meta-data provided and recorded. A web application, built using the Apache/php5/MySQL and Secure Sockets Layer (SSL) technologies, enable efficient capture and automatic submission of comprehensive experimental information at no cost to Centre staff time. Data collection is done through successive stages (Additional File 1) at which a comprehensive set of fields are presented, some of which are mandatory. Drop-down menus are available where possible to limit the use of free text and to facilitate data capture by minimising typing. Additional information can also be uploaded, for example Agilent Bioanalyser traces or Excel spreadsheets of quality control (QC) information. The successive stages follow a logical order and enable customised fields to be presented depending on choices applied in the previous step (Additional File 2).

Each stage was implemented in a flexible way to enable the easy capture of diverse experimental designs including complex pooling and splitting strategies. Data captured can be saved at any stage with the option to complete the remaining stages at a later time. Options to duplicate entries are available, where appropriate, to reduce the amount of typing for capturing details about multiple similar samples. The Online Annotation Tool is currently configured to capture data from gene expression studies including single-channel (Affymetrix 3', Exon and Gene arrays) and two-colour (Agilent) arrays, miRNA profiling, and can, in future, be extended to other microarray applications such as ChIP-on-chip. A detailed Help menu is available at each stage with comprehensive examples of experiment, sample or QC information recorded. The Online Annotation Tool allows users to rapidly and efficiently submit many experimental and QC details: it takes less than one hour to complete the entire process for the majority of experiments submitted to the Centre (involving up to 50 samples). Large scale experiments (with more than 200 samples) can be submitted using the Online Annotation Tool or via a standardised spreadsheet-based pipeline under development that can be customised for individual projects and parsed programmatically for storage into MiMiR.

Once all stages of data capture are completed online, the information provided is automatically checked for inconsistencies and missing data and is then ready for internal curation using the Curation and Annotation tools.

#### Curation and further annotation of experimental information

**Figure 2:**
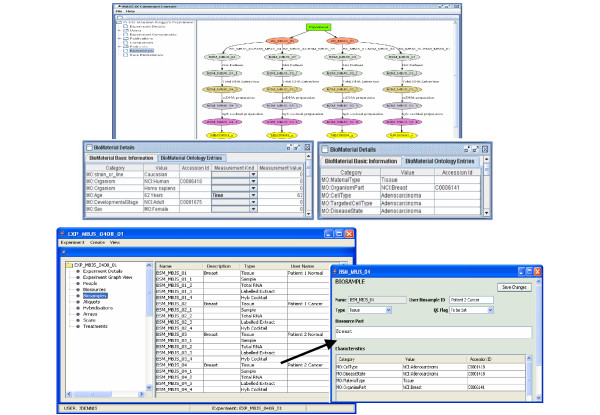
**Screen shots of the Curation and Annotation Tools. a: **Visualisation using the Curation Tool of experimental information submitted online. An object model of the experiment is programmatically built and represented graphically, where nodes represent the experiment and biomaterials (organisms with a prefix BS_ and samples with a prefix BSM_), and treatments are represented by arcs. Nodes are colour-coded to represent different stages of sample preparation; for example, beige and pink nodes correspond to the extracted total RNA and hybridisation cocktail, respectively. The biomaterial information supplied by users via the Online Annotation Tool is automatically displayed in the Curation Tool and assigned with MGED Ontology terms as indicated by the prefix "MO:" (bottom table views). Where appropriate NCI Metathesaurus terms and accession numbers are also automatically assigned and indicated by the "NCI:" prefix. **b:** Table views of biosample details in the Annotation Tool. The table view enables rapid validation of detailed information including sample types, descriptions and names assigned to samples by users. Double clicking on an item in the table opens a pop-up window (insert) where more detailed ontology information can be viewed and edited.

The experimental descriptions submitted by researchers via the Online Annotation Tool are programmatically extracted and assembled into an experiment by a specially designed Curation Tool. The Curation Tool is a Java application that uses an internal UML (Unified Modelling Language) object model to capture all the submitted information including details on experimental design, biosources and biosamples descriptions, compounds, protocols, treatment steps, user details and relevant publications, as well as the relationships between these entities. Automatically built experiment information is presented to annotators in a graphical form (Figure 2a) where nodes represent entities such as biosources, biosamples, treated biosamples, labelled extracts, hybridisation cocktails and scans, while arcs represent the actions required to move from one entity to another (i.e. treatment steps). MGED Ontology and NCI Metathesaurus terms are added to systematically describe certain experimental entities and can be viewed in the Curation Tool. Following creation of the experiment object model, the Annotation Tool is used to further annotate biomaterials and the relationships between them. The Annotation Tool is a Java application that displays information pertaining to biomaterials and hybridisations in a table view, enabling annotators to inspect subsets of data for consistency and accuracy, and to edit fields as appropriate (Figure 2b). MGED Ontology terms can be appended to experimental components using the existing MGED Ontology Viewer available through the Annotation Tool. A comprehensive user guide for the Annotation and Curation tools is available in Additional File 3.

### • Clinical data capture, annotation and link with microarray data

#### Ethical Framework

The storage and analysis of individual clinical and genetic data derived from human patients and volunteers is highly sensitive and requires that appropriate policies and procedures are defined in respect of ethical issues. MiMiR has been given formal approval to operate within strict guidelines under the jurisdiction of a Multi-centre Research Ethics Committee (MREC, Reference: 05/MREC05/69). The approval covers the handling of anonymised subjects clinical information which is typically recorded in hospital patient management systems. The ethical framework that governs the supply of data to the clinical part of MiMiR, called cMiMiR, and the subsequent use by researchers is described in Additional File 4 and Additional Files 5, 6, 7, 8.

#### Data Mapping Tool

Clinical data is commonly recorded in Access, Excel or similar databases that are used as routine patient management systems or clinical trial-specific databases. We developed a Data Mapping Tool to translate clinical information into codified clinical ontology terms and concepts and to allow for these descriptions to be imported into MiMiR in a standardised and structured way. Several coding schemes exist, providing recognised sets of unique concept identifiers. These include SNOMED-CT  and the Unified Medical Language Service (UMLS) . The UMLS was chosen and implemented in MiMiR as it is used by international efforts such as the National Cancer Institute caBIG™  and it can provide access to SNOMED-CT terms via its knowledge source web site . The UMLS API is used to map each entity in the source data to the corresponding clinical ontology term and the associated encoded values are then automatically assigned (Additional File 5). The resulting encoded record is represented in an XML format and linked in the database to the corresponding biosamples and experimental information. A comprehensive user guide with a detailed practical example showing screenshots of the various stages of clinical annotation is available in Additional File 9.

### • MiMiR Online experiment browser

**Figure 3:**
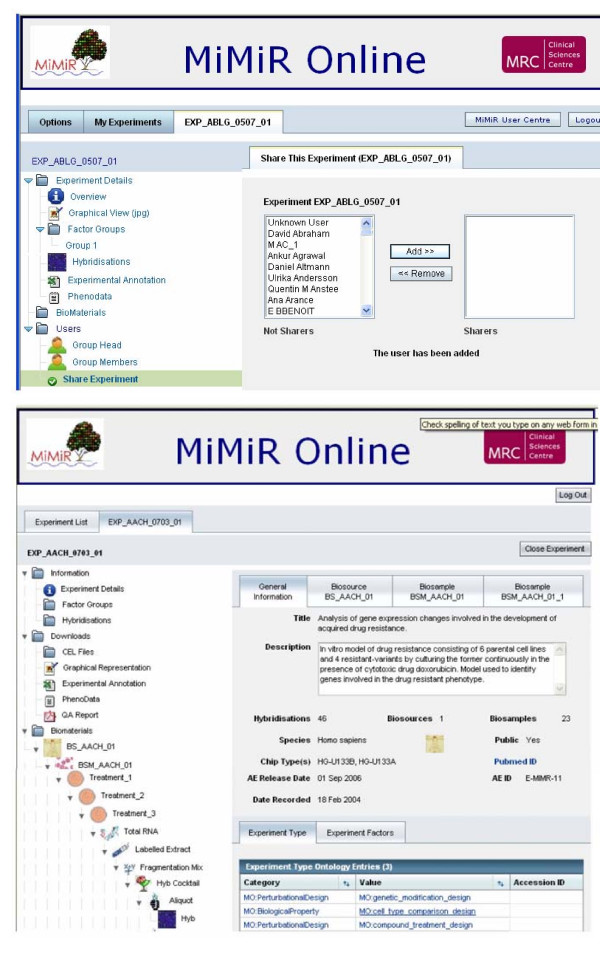
**Screen shots of MiMiR Online. a: **Screen shot of MiMiR Online showing the data sharing functionality. **b:** Screen shot of MiMiR Online showing the left hand tree view icons for navigation and the right hand panel showing the Experiment Details of a public experiment in MiMiR. Several experiments can be opened simultaneously and users can toggle between them using the top panel of tabs. Information including the study description, number of biosources (organisms), biosamples and hybridisations performed, chip type(s) used, private/public status, ArrayExpress accession number and date of public release (if relevant), as well as the active PubMed link if the experiment has been published, can be accessed. MGED Ontology terms are used systematically e.g. Category MO:PerturbationalDesign, Value MO:compound_treatment_design) and experimental factors (e.g. Category MO:compound, Value H2O2_-0.04). The tree view also gives access to information on the Biomaterials i.e. Biosources (whole organisms), Biosamples (material derived from the biosources) and the consecutive treatment steps generating the labelled extract to be hybridised on arrays. Different icons are used in the tree to visually facilitate navigation between the different procedure stages.

Detailed sample/treatment information for each experiment can be accessed via MiMiR Online web front end that communicates with the MiMiR database via the middleware layer. Registered users can view and access public datasets in MiMiR and users with appropriate rights for an experiment (e.g. the owner of a data set) can share un-published experiments with other registered users of the system via the interface (Figure 3a). Two international consortia are currently using MiMiR to centralise and share un-published datasets (European Rat tools for functional Genomics (EURATools)  and Wellcome Trust Cardiovascular Functional Genomics consortium).

Upon logging into MiMiR Online, a list of available experiments is displayed and each experiment can be individually selected to visualise the design, sample and hybridisation details (Figure 3b). Users can navigate through the comprehensive experimental information recorded including factors, biosources, biosamples, treatments, labelled extracts, protocols. Quality control assessments are displayed at several key steps (e.g. total RNA, labelled extract and scan) to flag potentially problematic samples that may lead to unreliable data.

The raw data files can be downloaded from several locations either in bulk (all the files for a single experiment), by factor group or for single hybridisation. Clicking on the 'Download' icon initiates retrieval of the relevant files from the database, which are zipped and sent to the web browser. A processing bar monitors the progress of the download which typically takes less than 30 seconds per .CEL file for an Affymetrix U133Plus 2.0 array.

The quality and reliability of each dataset is assessed by looking at a number of quality assessment (QA) plots and metrics generated using the BioConductor open source software framework  and the Affymetrix Expression Console™ software  that are compiled into a comprehensive QA report available for download in pdf format (Figure 3b). A comprehensive list of pheno-data e.g. experimental factors, biological and technical variables, can also be downloaded and saved in the appropriate format suitable for import into both open source (Bioconductor) or proprietary (Partek.^®^) software. This can be extremely useful to analyse systematic errors (e.g. technical batch effect) alongside studied biological effects.

### • Data Analysis using EMAAS

**Figure 4:**
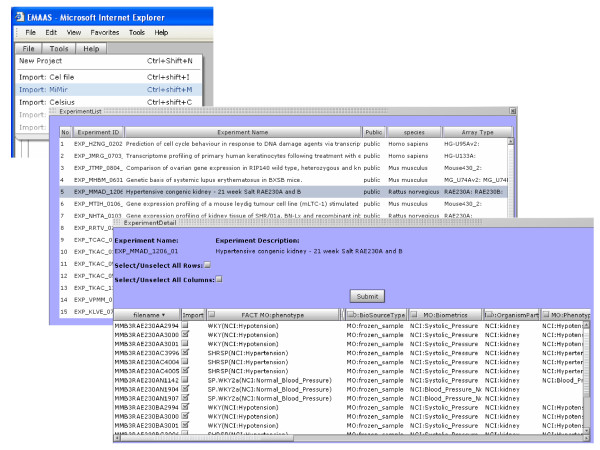
Screen shots of the MiMiR-EMAAS interface, showing a guest user viewing and selecting data and meta-data from a MiMiR experiment to export into EMAAS for analysis.

EMAAS (**E**xtensible **M**icro **A**rray **A**nalysis **S**ystem) is a new web e-support application developed for microarray data analysis . EMAAS utilizes grid technologies to perform analysis tasks, programmatically calling analysis packages such as R-BioConductor and the Affymetrix Power Tools . EMAAS also uses web services to various online facilities such as DAVID [22], CELSIUS [23] and GeneCards [24]. MiMiR registered users can inspect MiMiR experiments and associated information from the EMAAS-MiMiR integrated interface and can select specific data files and associated meta-data to be imported into EMAAS for analysis (Figure 4). The MiMiR middleware is used by EMAAS to securely access the appropriate experimental information, using Java Server Pages (JSPs) and Servlets. EMAAS is currently being used to perform data quality assessment, pre-processing, statistical analysis and functional enrichment analysis of Affymetrix 3' and Exon/Gene ST arrays, with scope to add further functionality for other platforms such as Illumina, Agilent and Codelink arrays [20].

### • MAGE-ML export pipelines to ArrayExpress and Resolver

Data in MiMiR is sent to ArrayExpress upon publication and the original ArrayExpress export pipeline has been re-engineered into a more generic tool. A model-driven approach was adopted, whereby a local UML model was designed to represent all experimental meta-data that is required for a valid MAGE-ML submission to ArrayExpress or to the Resolver analysis package. Two sets of Java classes are created to first interrogate the MiMiR/middleware layer and extract data elements, and to then populate a MAGEstk/Java data model to generate a MAGE-ML (xml) file. The ArrayExpress validation toolkit  is incorporated into the MAGE-ML building process to provide automated validation of MAGE-ML files generated for export to the relevant system. A total of 24 experiments (corresponding to 730 whole genome arrays) have been submitted to ArrayExpress to date and all the experiment annotations are of the highest quality, as confirmed by the highest MIAME score [8] assigned by ArrayExpress.

## Utility and discussion

It is recognised that inconsistent and low quality experimental annotation in public data repository significantly compromises the re-use of microarray data for meta-analysis [1,23]. MiMiR was designed to overcome this major limitation. Users can submit experimental information in an easy, fast and secure way via the web. The meta-data is collected and stored in MiMiR at the time of data generation rather than at the point of publication and submission to ArrayExpress or GEO, which can take up to several years. As a result, MiMiR captures more accurate and comprehensive experiment information than public repositories and most other microarray databases, and therefore provides rich experimental details often required for data mining and cross-experiment re-analysis. The experimental annotation process is efficiently performed by programmatically building the experiment structure from the submitted information and automatically populating over 60 percent of the required fields. This is recognised as a major advantage and other systems are looking at improving the performance and speed of sample annotation [25].

Data is centralised in MiMiR in a highly secure way enabling researchers to share data prior to publication: this is particularly useful for the national and international consortia that MiMiR supports. MiMiR is compliant with MAGE and uses MAGE-ML for data exchange with other MAGE databases (e.g. ArrayExpress and Resolver) rather than the simplified MAGE-TAB format [26].

MiMiR is fully integrated with the Rosetta Resolver analysis package and experimental information is automatically built in Resolver from annotations stored in MiMiR. Analysis of MiMiR data can also be done using the freely available EMAAS portal [20]. The EMAAS user base is growing very rapidly and the system is continuously being updated with latest analysis algorithms to support new chip types and applications.

It is well known that molecular signatures derived from microarray clinical studies can be unstable and highly dependant on the selection of patients used in the training set [27]. Michiels et al. for example, found that five of the seven largest published studies addressing cancer prognosis did not classify patients better than chance [28]. Good validation of prognostic or predictive gene expression profiles requires large patient cohort and the clinical part of MiMiR could be used as a platform to build centralised data sets for this purpose.

MiMiR stores raw unprocessed microarray data like in GEO and ArrayExpress in order to maximise the long term value of datasets and enable processing and re-analysis of data. However normalisation is necessary in order to mine data across different experiments and we are planning to develop a dynamic normalisation pipeline to allow such comparisons. We also envisage to develop standard analysis pipelines to generate lists of differentially expressed genes that will be made available for mining, querying and further analysis. Query and search functionalities will be implemented in the system to interrogate and retrieve datasets of interest for example by species, tissue or array type.

## Conclusion

MiMiR is a mature microarray data warehouse containing over 3000 arrays worth of data for mining and analysis and supports over 200 research groups, including two international consortia. MiMiR is not a new microarray public repository but it provides a secure environment for collection, capture, consistent annotation, visualisation and dissemination of data to our large user community and collaborators. The clinical part of MiMiR also represents a unique resource for clinicians and researchers to effectively share, mine and analyse clinical information and large scale molecular profiling data within an ethically approved environment. Analysis of MiMiR data is enabled through integration with commercial and freeware analysis packages and will be enhanced by additional normalisation and analysis pipelines. MiMiR is a powerful, scalable and flexible resource that can potentially be extended to new data modalities like next generation sequencing data for which similar ethical, social and clinical constrains apply and are beginning to be addressed by the research and clinical communities [29,30].

## Availability and requirements

MiMiR Online and the Online Annotation Tool can be accessed from the Microarray Centre-MiMiR User Centre web site . The code for the Curation and Annotation tools as well as the MAGE-ML export pipeline and the Data Mapping Tool can be made available on request. A comprehensive user manual for the Annotation and Curation tools is also available from the Microarray Centre web site. The tools have been optimised for Windows environment and, although untested, could be used with other operating systems.

## Authors' contributions

CT designed the software architecture and coordinated the software development. CT designed and wrote MiMiR Online and the MiMiR User Centre web site. AB and JD designed the Online Annotation Tool and CT implemented it. CaT, JD, AB, TB and LG gathered the requirements for the Curation and Annotation tools and MT, CT, SA and NC were involved in building the tools. SA designed and implemented the middleware layer and the security infrastructure. EB, AB, and CT worked on the QA reports and pheno-data extraction. TC coordinated the development of the clinical part of MiMiR, re-engineered the MAGE-ML export pipelines and put in place the ethical framework with TA. PS worked on clinical mapping concepts for designing the Data Mapping Tool. MT and SA worked on the deployment and maintenance of all the applications. NC and GB developed the integration and interface between EMAAS and MiMiR. KM tested the Online Annotation Tool and MiMiR Online. LG and TA guided and coordinated the execution of the project. LG wrote the manuscript. All authors contributed to scientific discussions and have read and approved the final manuscript.

## Supplementary Material

Additional File 1 Stages of experimental information collection using the Online Annotation Tool. Experiment and sample information are collected using a series of online forms starting with an overview of the experiment (stages 1–4), followed by detailed information pertaining to organisms, arrays and samples (stages 5–7). Specific details of protocols used (stages 8–9) and Quality Control parameters (stages 10–12) are also collected. There are two key decision stages (stages 5 and 9) which determine the fields presented to users in subsequent stages. Click here for file

Additional File 2 Example of context dependent stages in the Online Annotation Tool. Several stages in the data collection process are context dependent with specific compulsory fields being presented according to information provided in a previous step. For example, at stage 5 researchers define the type of array used which subsequently determines which corresponding labelling protocols and services are available and displayed in the dropdown at stage 9. The choice of one specific labelling protocol, in turn, determines the relevant Quality Control information to be collected at stage 10. Click here for file

Additional File 3 Curation and Annotation tools user guide. Click here for file

Additional File 4 Ethical framework for the supply of clinical data to the clinical part of MiMiR (cMiMiR) and subsequent data retrieval from researchers. Suppliers of microarray and clinical data, e.g. clinicians/researchers running a clinical trial with independent ethical approval, create and assign unique anonymisation keys (Level 4 pseudo-anonymised data) for each participating patient. All identifying information from the respective clinical record (e.g. name, address, date of birth) is removed before sending these records for storage into cMiMiR. A 'cMiMiR Supplier Agreement' between Suppliers and Custodians of cMiMiR covers the requirements and obligations of both parties regarding the import of clinical records into cMiMiR (Additional File 6). Researchers, designated "Subscribers", wishing to access data in cMiMiR for a particular project need to apply for Research Ethics Committee (REC) approval before they can be granted rights to access data (and be bound by the 'cMiMiR Subscriber Agreement', Additional File 7). Suppliers can collect and store additional information in cMiMiR for existing patients (e.g. treatment follow-up data) by updating the relevant cMiMiR record using the original anonymisation key. Trained annotators implement the updates to ensure consistency in annotation and quality and integrity of the data. Patients and volunteers involved in clinical projects have the right to withdraw their data from cMiMiR at any stage and without any justification. This is done by the Supplier who conducted the clinical trial and who holds the anonymisation key to the individual record. It is important to ensure that patients and volunteers participating in clinical trials are fully aware of the use of their anonymised clinical records for analysis by authorised researchers during the consent process. Clinical and genetic data derived from biological materials stored in licensed tissue banks are covered under standard consenting process. Prospective clinical trials explicitly consent all recruited participants using a 'cMiMiR consent form' (Additional File 8). For retrospective clinical microarray trials under present MREC guidelines, re-consent of participants is required if re-use and sharing of clinical and genetic information is not covered in the original trial consent protocol. Click here for file

Additional File 5 Example of clinical information supplied by a clinical researcher (first three columns) and how it is codified using the Data Mapping Tool prior to import into MiMiR. Each concept must be explicitly mapped to unambiguous, uniquely identified terms. The Unified Medical Language System (UMLS version2007AB) which provides a Metathesaurus of clinical terms is used to provide the identifiers that are persisted within MiMiR. Click here for file

Additional File 6 cMiMiR Supplier Agreement. Click here for file

Additional File 7 cMiMiR Subscriber Agreement. Click here for file

Additional File 8 cMiMiR Consent Form. Click here for file

Additional File 9 cMiMiR Data Mapping Tool user guide and practical example. Click here for file

## References

[B1] Larsson O, Sandberg R (2006). Lack of correct data format and comparability limits future integrative microarray research. Nat Biotechnol.

[B2] Stoeckert C, Parkinson H (2003). The MGED Ontology: a framework for describing functional genomics experiments. Comparitive and Functional Genomics.

[B3] Whetzel PL, Parkinson H, Causton HC, Fan L, Fostel J, Fragoso G, Game L, Heiskanen M, Morrison N, Rocca-Serra P (2006). The MGED Ontology: a resource for semantics-based description of microarray experiments. Bioinformatics.

[B4] Spellman PT, Miller M, Stewart J, Troup C, Sarkans U, Chervitz S, Bernhart D, Sherlock G, Ball C, Lepage M (2002). Design and implementation of microarray gene expression markup language (MAGE-ML). Genome Biol.

[B5] Brazma A, Hingamp P, Quackenbush J, Sherlock G, Spellman P, Stoeckert C, Aach J, Ansorge W, Ball CA, Causton HC (2001). Minimum information about a microarray experiment (MIAME)-toward standards for microarray data. Nat Genet.

[B6] Strauss E (2006). Arrays of hope. Cell.

[B7] Barrett T, Troup DB, Wilhite SE, Ledoux P, Rudnev D, Evangelista C, Kim IF, Soboleva A, Tomashevsky M, Edgar R (2007). NCBI GEO: mining tens of millions of expression profiles–database and tools update. Nucleic Acids Res.

[B8] Parkinson H, Kapushesky M, Shojatalab M, Abeygunawardena N, Coulson R, Farne A, Holloway E, Kolesnykov N, Lilja P, Lukk M (2007). ArrayExpress–a public database of microarray experiments and gene expression profiles. Nucleic Acids Res.

[B9] Smith CM, Finger JH, Hayamizu TF, McCright IJ, Eppig JT, Kadin JA, Richardson JE, Ringwald M (2007). The mouse Gene Expression Database (GXD): 2007 update. Nucleic Acids Res.

[B10] Saal LH, Troein C, Vallon-Christersson J, Gruvberger S, Borg A, Peterson C (2002). BioArray Software Environment (BASE): a platform for comprehensive management and analysis of microarray data. Genome Biol.

[B11] Gollub J, Ball CA, Binkley G, Demeter J, Finkelstein DB, Hebert JM, Hernandez-Boussard T, Jin H, Kaloper M, Matese JC (2003). The Stanford Microarray Database: data access and quality assessment tools. Nucleic Acids Res.

[B12] Mazzarelli JM, Brestelli J, Gorski RK, Liu J, Manduchi E, Pinney DF, Schug J, White P, Kaestner KH, Stoeckert CJ (2007). EPConDB: a web resource for gene expression related to pancreatic development, beta-cell function and diabetes. Nucleic Acids Res.

[B13] Pan F, Chiu CH, Pulapura S, Mehan MR, Nunez-Iglesias J, Zhang K, Kamath K, Waterman MS, Finch CE, Zhou XJ (2007). Gene Aging Nexus: a web database and data mining platform for microarray data on aging. Nucleic Acids Res.

[B14] Splendiani A, Brandizi M, Even G, Beretta O, Pavelka N, Pelizzola M, Mayhaus M, Foti M, Mauri G, Ricciardi-Castagnoli P (2007). The genopolis microarray database. BMC Bioinformatics.

[B15] Marzolf B, Deutsch EW, Moss P, Campbell D, Johnson MH, Galitski T (2006). SBEAMS-Microarray: database software supporting genomic expression analyses for systems biology. BMC Bioinformatics.

[B16] Demeter J, Beauheim C, Gollub J, Hernandez-Boussard T, Jin H, Maier D, Matese JC, Nitzberg M, Wymore F, Zachariah ZK (2007). The Stanford Microarray Database: implementation of new analysis tools and open source release of software. Nucleic Acids Res.

[B17] Ameur A, Yankovski V, Enroth S, Spjuth O, Komorowski J (2006). The LCB Data Warehouse. Bioinformatics.

[B18] Le Brigand K, Barbry P (2007). Mediante: a web-based microarray data manager. Bioinformatics.

[B19] Navarange M, Game L, Fowler D, Wadekar V, Banks H, Cooley N, Rahman F, Hinshelwood J, Broderick P, Causton HC (2005). MiMiR: a comprehensive solution for storage, annotation and exchange of microarray data. BMC Bioinformatics.

[B20] Barton G, Saleem A, Krznaric M, Abbott J, MJ S, Tiwari B, Aitman T, Game LJMS, Huang Y (2008). EMAAS: An extensible grid-based portal for microarray data analysis and management. BMC Bioinformatics.

[B21] The Chipping Forecast II (2002). Supplement to Nature Genetics.

[B22] Sherman BT, Huang da W, Tan Q, Guo Y, Bour S, Liu D, Stephens R, Baseler MW, Lane HC, Lempicki RA (2007). DAVID Knowledgebase: a gene-centered database integrating heterogeneous gene annotation resources to facilitate high-throughput gene functional analysis. BMC Bioinformatics.

[B23] Day A, Carlson MR, Dong J, O'Connor BD, Nelson SF (2007). Celsius: a community resource for Affymetrix microarray data. Genome Biol.

[B24] Safran M, Chalifa-Caspi V, Shmueli O, Olender T, Lapidot M, Rosen N, Shmoish M, Peter Y, Glusman G, Feldmesser E (2003). Human Gene-Centric Databases at the Weizmann Institute of Science: GeneCards, UDB, CroW 21 and HORDE. Nucleic Acids Res.

[B25] Draghici S, Tarca AL, Yu L, Ethier S, Romero R (2008). KUTE-BASE: storing, downloading and exporting MIAME-compliant microarray experiments in minutes rather than hours. Bioinformatics.

[B26] Rayner TF, Rocca-Serra P, Spellman PT, Causton HC, Farne A, Holloway E, Irizarry RA, Liu J, Maier DS, Miller M (2006). A simple spreadsheet-based, MIAME-supportive format for microarray data: MAGE-TAB. BMC Bioinformatics.

[B27] Abdullah-Sayani A, Bueno-de-Mesquita JM, Vijver MJ van de (2006). Technology Insight: tuning into the genetic orchestra using microarrays–limitations of DNA microarrays in clinical practice. Nat Clin Pract Oncol.

[B28] Michiels S, Koscielny S, Hill C (2005). Prediction of cancer outcome with microarrays: a multiple random validation strategy. Lancet.

[B29] McGuire AL, Cho MK, McGuire SE, Caulfield T (2007). Medicine. The future of personal genomics. Science.

[B30] McGuire AL, Caulfield T, Cho MK (2008). Research ethics and the challenge of whole-genome sequencing. Nat Rev Genet.

