# Supplementary material for: MiMiR – an integrated platform for microarray data sharing, mining and analysis
Source: BMC Bioinformatics. 2008 Sep 18;9:379. doi: 10.1186/1471-2105-9-379 (PMC2572073; doi:10.1186/1471-2105-9-379)
Supplement: Additional File 1 — Stages of experimental information collection using the Online Annotation Tool. Experiment and sample information are collected using a series of online forms starting with an overview of the experiment (stages 1–4), followed by detailed information pertaining to organisms, arrays and samples (stages 5–7). Specific details of protocols used (stages 8–9) and Quality Control parameters (stages 10–12) are also collected. There are two key decision stages (stages 5 and 9) which determine the fields presented to users in subsequent stages. [file 1471-2105-9-379-S1.pdf]

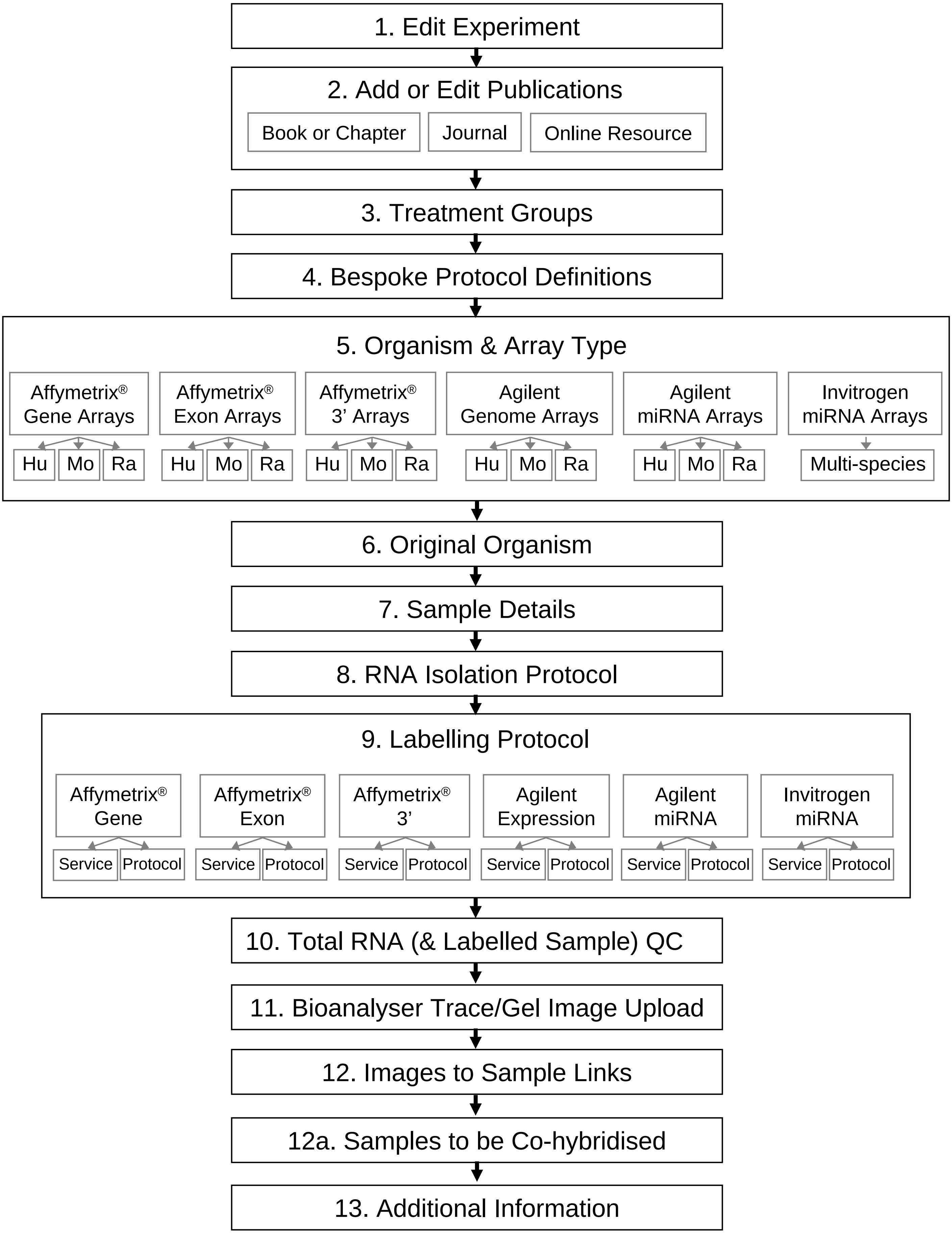
1. Edit Experiment
2. Add or Edit Publications
Book or Chapter
Journal
Online Resource
3. Treatment Groups
4. Bespoke Protocol Definitions
5. Organism & Array Type
Affymetrix® Gene Arrays
Hu
Mo
Ra
Affymetrix® Exon Arrays
Hu
Mo
Ra
Affymetrix® 3’ Arrays
Hu
Mo
Ra
Agilent Genome Arrays
Hu
Mo
Ra
Agilent miRNA Arrays
Hu
Mo
Ra
Invitrogen miRNA Arrays
Multi-species
6. Original Organism
7. Sample Details
8. RNA Isolation Protocol
9. Labelling Protocol
Affymetrix® Gene
Service
Protocol
Affymetrix® Exon
Service
Protocol
Affymetrix® 3’
Service
Protocol
Agilent Expression
Service
Protocol
Agilent miRNA
Service
Protocol
Invitrogen miRNA
Service
Protocol
10. Total RNA (& Labelled Sample) QC
11. Bioanalyser Trace/Gel Image Upload
12. Images to Sample Links
12a. Samples to be Co-hybridised
13. Additional Information
