# Supplementary material for: MiMiR – an integrated platform for microarray data sharing, mining and analysis
Source: BMC Bioinformatics. 2008 Sep 18;9:379. doi: 10.1186/1471-2105-9-379 (PMC2572073; doi:10.1186/1471-2105-9-379)
Supplement: Additional File 2 — Example of context dependent stages in the Online Annotation Tool. Several stages in the data collection process are context dependent with specific compulsory fields being presented according to information provided in a previous step. For example, at stage 5 researchers define the type of array used which subsequently determines which corresponding labelling protocols and services are available and displayed in the dropdown at stage 9. The choice of one specific labelling protocol, in turn, determines the relevant Quality Control information to be collected at stage 10. [file 1471-2105-9-379-S2.pdf]

Organism and Array Type

Stage 5 of 13

Organism *: Homo sapiens

If other organism please state

Array Type *: Please Select

- Please Select
- Human Exon 1.0 ST Array
- Human Gene 1.0 ST Array
- Human Genome U133A 2.0 Array
- Human Genome U133 Plus 2.0 Array
- Mouse Gene 1.0 ST Array
- Mouse Exon 1.0 ST Array
- Mouse Genome 430 2.0 Array
- Mouse Genome 430A 2.0 Array

If other please state

Continue

Labelling Protocol

Stage 9 of 13

Labelling Protocol *: Please Select

- Please Select
- Whole Transcript 100ng Labelling Protocol
- Whole Transcript 100ng Labelling Service
- Whole Transcript 1ug Labelling Service
- Whole Transcript 1ug Labelling Protocol

Total RNA and cDNA Quality Control

Stage 10 of 13

Labelling Protocol: Whole Transcript 100ng Labelling Protocol

Sample Name *	Total RNA concentration (μg/μl) *	Total RNA Purity A260/A280 *	cDNA concentration (ng/μl) *	cDNA Purity A260/A280 *	cDNA volume eluted (μl) *
Patient 1 Normal	0.283	1.92	463.96	1.98	26
Patient 2 Normal	0.319	1.98	428.86	2.01	26
Patient 1 Cancer	0.276	2.09	422.52	1.99	26
Patient 2 Cancer	0.262	2.03	451.62	1.98	27

Labelling Protocol: Whole Transcript 1ug Labelling Protocol

Sample Name *	Total RNA concentration (μg/μl) *	Total RNA Purity A260/A280 *	Reduced RNA concentration (ng/μl) *	Reduced RNA Purity A260/A280 *	cDNA concentration (ng/μl) *	cDNA Purity A260/A280 *	cDNA volume eluted (μl) *
Patient 1 Normal	0.283	1.92	56.92	2.01	447.13	1.97	26
Patient 2 Normal	0.319	1.98	59.35	2.06	446.74	2.03	26
Patient 1 Cancer	0.276	2.09	62.21	1.99	427.67	2.01	27
Patient 2 Cancer	0.262	2.03	66.92	2.04	428.96	1.99	25
