# Supplementary material for: MiMiR – an integrated platform for microarray data sharing, mining and analysis
Source: BMC Bioinformatics. 2008 Sep 18;9:379. doi: 10.1186/1471-2105-9-379 (PMC2572073; doi:10.1186/1471-2105-9-379)
Supplement: Additional File 3 — Curation and Annotation tools user guide. [file 1471-2105-9-379-S3.doc]

[System requirement and installation of the Curation and Annotation tools 3](#__RefHeading___Toc200514282)

[• System requirements 3](#__RefHeading___Toc200514283)

[• Installation 3](#__RefHeading___Toc200514284)

[• Login/password 3](#__RefHeading___Toc200514285)

[Part 1: Curation Tool 3](#__RefHeading___Toc200514286)

[1. Report file from the Online Annotation Tool 3](#__RefHeading___Toc200514287)

[2. Selecting an experiment to be curated 3](#__RefHeading___Toc200514288)

[3. Experiment Window Summary 4](#__RefHeading___Toc200514289)

[4. Experiment Details module 5](#__RefHeading___Toc200514290)

[5. Users module 6](#__RefHeading___Toc200514291)

[a Existing Users: 7](#__RefHeading___Toc200514292)

[b New Users: 7](#__RefHeading___Toc200514293)

[6. Experiment Components module 8](#__RefHeading___Toc200514294)

[a Experiment Design tab: 8](#__RefHeading___Toc200514295)

[b Experiment Resources tab: 9](#__RefHeading___Toc200514296)

[c Experiment Factors tab: 9](#__RefHeading___Toc200514297)

[d Experiment Users tab: 10](#__RefHeading___Toc200514298)

[7. Publications module 11](#__RefHeading___Toc200514299)

[8. Compound module 11](#__RefHeading___Toc200514300)

[a Component Compounds 11](#__RefHeading___Toc200514301)

[b Composite Compounds 12](#__RefHeading___Toc200514302)

[9. Protocols Module 13](#__RefHeading___Toc200514303)

[a Bespoke protocols 13](#__RefHeading___Toc200514304)

[b Total RNA extraction protocols 14](#__RefHeading___Toc200514305)

[c Labelling protocols 14](#__RefHeading___Toc200514306)

[10. Biomaterials module 15](#__RefHeading___Toc200514307)

[Part 2: Annotation Tool 17](#__RefHeading___Toc200514308)

[1. Selecting an experiment to be annotated 17](#__RefHeading___Toc200514309)

[2. Experiment Window Summary 17](#__RefHeading___Toc200514310)

[3. General Functions 19](#__RefHeading___Toc200514311)

[• Saving to the database 19](#__RefHeading___Toc200514312)

[• Creating new protocols/compounds 19](#__RefHeading___Toc200514313)

[• Viewing protocols/compounds 20](#__RefHeading___Toc200514314)

[4. Experiment Details 20](#__RefHeading___Toc200514315)

[a Design tab: 20](#__RefHeading___Toc200514316)

[b Repository tab: 21](#__RefHeading___Toc200514317)

[c Factors tab: 21](#__RefHeading___Toc200514318)

[d References tab: 21](#__RefHeading___Toc200514319)

[5. Experiment Graph View 22](#__RefHeading___Toc200514320)

[• Annotation Tool Views: 22](#__RefHeading___Toc200514321)

[• Using the Graph view to represent pooling 23](#__RefHeading___Toc200514322)

[• Using the Graph view to represent splitting 25](#__RefHeading___Toc200514323)

[6. People 26](#__RefHeading___Toc200514324)

[7. Biosources/Biosamples 26](#__RefHeading___Toc200514325)

[• Information populated by the Curation Tool: 26](#__RefHeading___Toc200514326)

[8. Protocols 30](#__RefHeading___Toc200514327)

[9. Aliquots 30](#__RefHeading___Toc200514328)

[10. Hybs, Arrays and Scans 31](#__RefHeading___Toc200514329)

[• Table View Modules: 33](#__RefHeading___Toc200514330)

[• Annotation Checking Procedure 33](#__RefHeading___Toc200514331)

**System requirement and installation of the Curation and Annotation tools**

- System requirements

Operating system: Windows XP or UNIX with minimum of 512MB RAM memory.

Java version: J2SE1.5 and above.

- Installation

Each tool is packaged into a single jar file. The jar files are located in a common folder on the network and can be invoked using java runtime environment.

- Login/password

A username and password are required to log in to both the Curation Tool and the Annotation Tool. The administrator will provide new annotators with a username and password.

**Part 1: Curation Tool**

1. **Report file from the Online Annotation Tool**

Users are required to submit their experimental information via the **Online Annotation Tool** before their samples are hybridised by Microarray Centre staff. An Excel report file containing the details supplied by the user is automatically generated and is used as a reference throughout the curation and annotation process.

Log into the Microarray Centre intranet website, select MiMiR Web Forms ‘Get Annotation Report for User Submitted Experiment’ and enter the relevant experiment ID. This will email you the corresponding annotation report file, along with the bioanalyser trace QC files uploaded into the Online Annotation Tool by the user. The files will be compressed into a zip file and attached to the email.

On submission of an experiment via the Online Annotation Tool the curator should receive an automated email. This will contain a notification if a new organism or chip type is used which is not already entered into the MIMIR database. This email should be checked by the curator and, if necessary, a new organism and chip type record should be created in the database before curation starts.

1. **Selecting an experiment to be curated**

Log into the Curation Tool and a window displaying the list of experiments to be curated will appear. Select the appropriate experiment to be curated (Fig. 1).


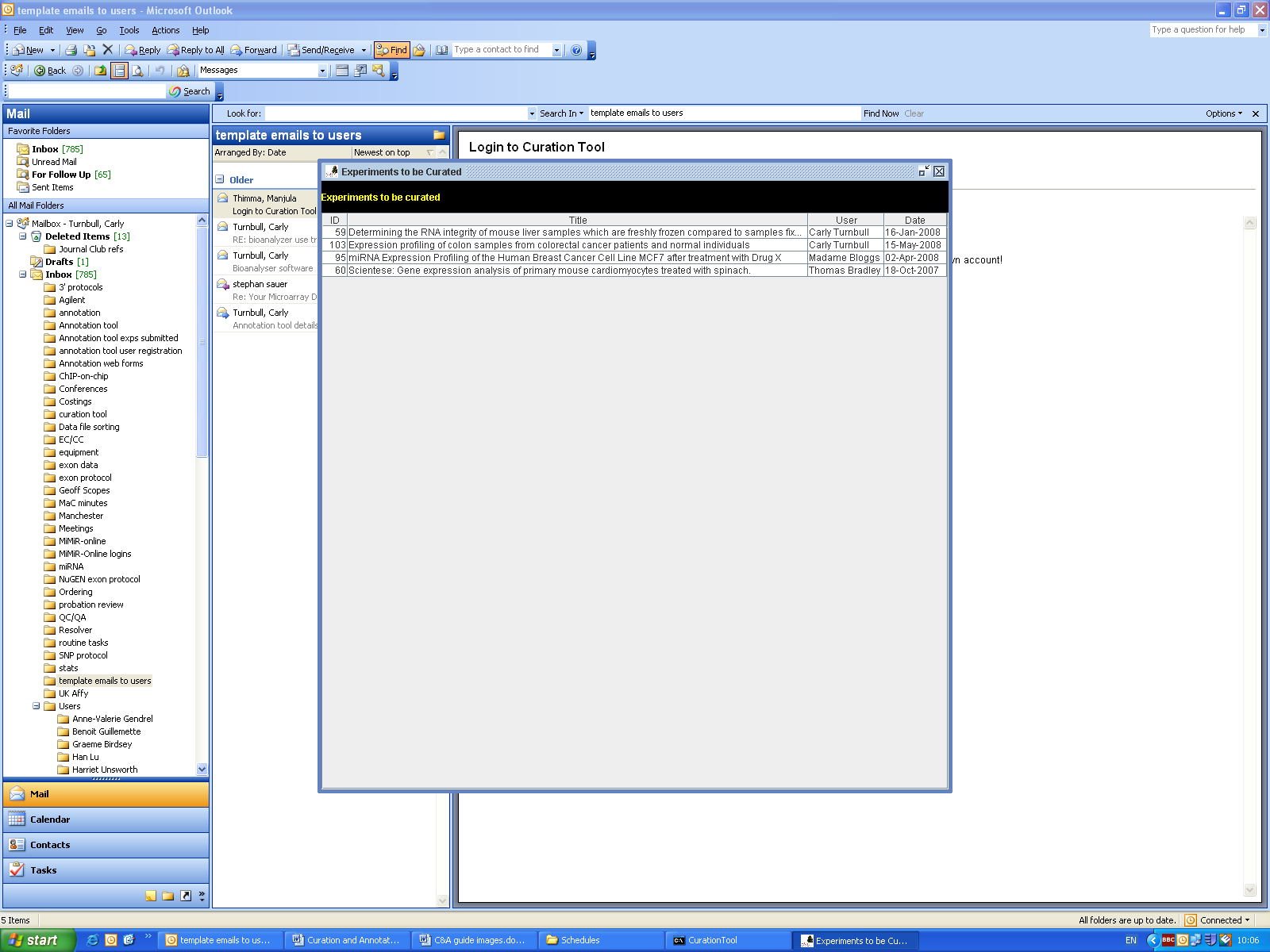


Fig. 1 Screenshot of the ‘Experiments to be curated’ window.

This window will stay open allowing the curator to access more than one experiment at once. The Curation Tool is designed so that an experiment is curated in one sitting and this should take no more than an hour.

The experiment selected is then built in the Curation Tool. The submitted information is read and the majority of the fields in the tool are automatically populated. A progress bar and description give an indication of when this is near completion.

1. **Experiment Window Summary**

Once the experiment has been built an experiment window will open (Fig 2). The left-hand panel of the experiment window displays the various modules of the Curation Tool (Fig. 2). The experiment has been given an ID number (top of the tree) and the relevant user is listed.


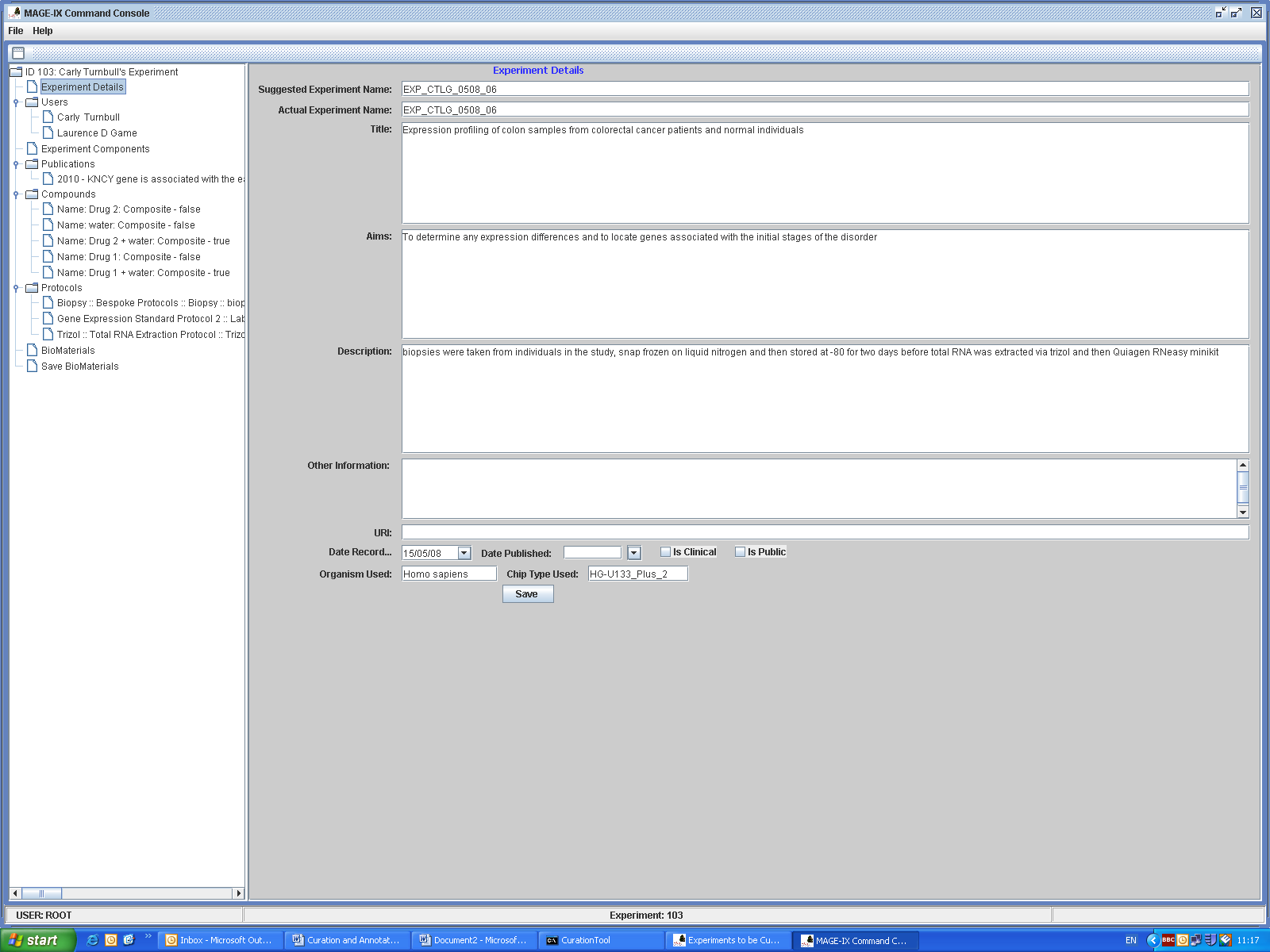


Fig. 2 Close-up of the left-hand panel in the experiment window.

The Curation Tool modules comprise:

- Experiment Details

This module allows the curator to see the details of the design of the experiment and the aims, and gives a good overview of the experiment.

- Users

This shows each user record and highlights which users are new and which already exist in the MIMIR database

- Experiment Components

This module contains information on the experimental design, factors and users linked to the experiment. In the Users tab individuals can be linked to this experiment and be assigned roles.

- Publications

This module gives details of any publications taken into account when the experiment was designed, or any work that the current study is based on.

- Compounds

This module describes the compounds that the biomaterials were treated with during the experiment.

- Protocols

This contains the protocols used during this experiment, along with their descriptions.

- Biomaterials

This describes the biological material used for the experiment. Biosources are defined as the organisms used in the experiment (e.g. a cancer patient or a mouse model) and the biosamples are the samples derived from the biosources that are treated or investigated for the purpose of the experiment (e.g. colon tissue biopsy from a patient).

Each module is described in more detail in the following pages.

1. **Experiment Details module**

The Curation Tool assigns an experiment name based on the information already stored in the MIMIR database (Fig. 3).


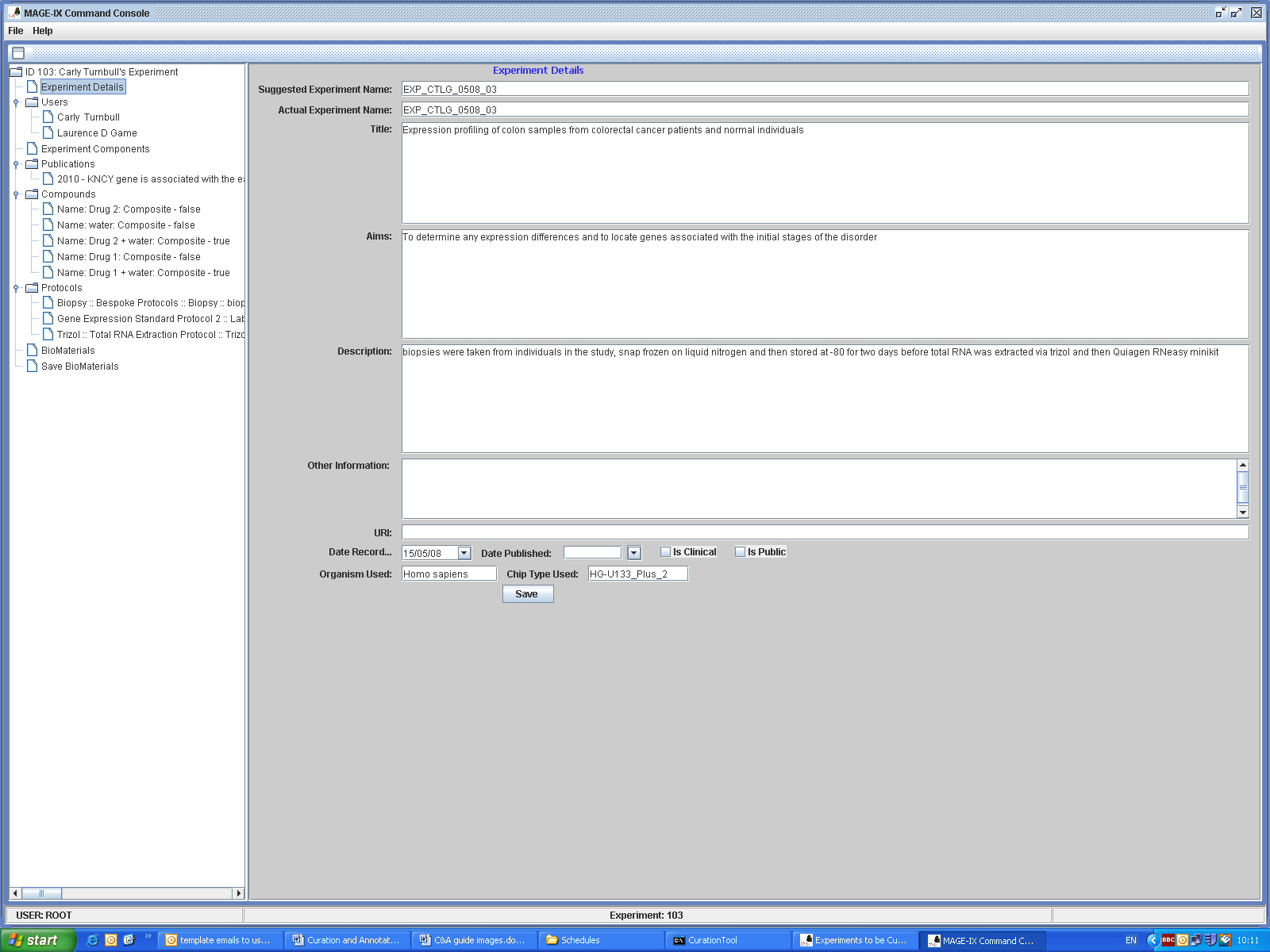


Fig. 3 Screenshot of the Experiment Details window.

Experiment names follow this convention:

- prefix EXP
- 2 initials of the user followed by the 2 initials of the group head
- four digits are the month and year in which the samples were received
- two digits denote the experiment number for that user irrespective of the date

For example EXP_CTLG_0508_03

User: Carly Turnbull Samples received: 13/05/08

Group Head: Laurence Game Experiment Number: 03

The experiment name is automatically generated by the Curation Tool and populated in the Suggested Experiment Name field. Below this field is the Actual Experiment Name field where the curator can change the experiment name if necessary.

The Title and the Description boxes can be edited to follow a standardised way of describing the experiment. The details in the Aims and Other Information boxes are used to give the curator a better idea of the experimental design, in case this is not clear from the title and description, but these fields are not recorded in the database. A URI box is available to record a relevant web page.

The Date Recorded is automatically populated with the date the curation was started and can be amended if necessary. There is an option to enter the date of publication of the study and whether it is public or not. This information may be entered if the experiment comes from a collaborator and was run some time ago, but will not be relevant for the majority of experiments. This information can also be entered through the Annotation Tool. Experiments involving patient samples can be flagged as clinical by ticking the ‘Is Clinical’ box.

The Organism Used and the Chip Type Used are automatically populated from the Online Annotation Tool and this can be double-checked against the arrays received from the user before the hybridisation is performed. This information is not currently populated in the Annotation Tool, but will be utilised with the Laboratory Data Mapping Tool which is under development.

Once the curator is happy that the details on this page are correct they can save it; this will create a new experiment in the MIMIR database. This step is necessary in order to curate any other modules in the tool. Once the experiment module has been saved it will be locked for editing and any further changes can only be made downstream in the Annotation Tool.

1. **Users module**

The information provided during registration for the Online Annotation Tool is displayed here. This module displays user records and whether they are present in the database, these records will be linked to the experiment later, through the Users tab of the Experiment Components module

1. Existing Users:

Users who already exist in the MIMIR database (i.e. those who have run previous experiments with the Microarray Centre) will be recognised by the tool and coloured in yellow. The experiments each user has access to and the roles assigned to them for each of these experiments will be displayed at the bottom of the window (Fig. 4a). The contact details are displayed but cannot be edited at this stage.


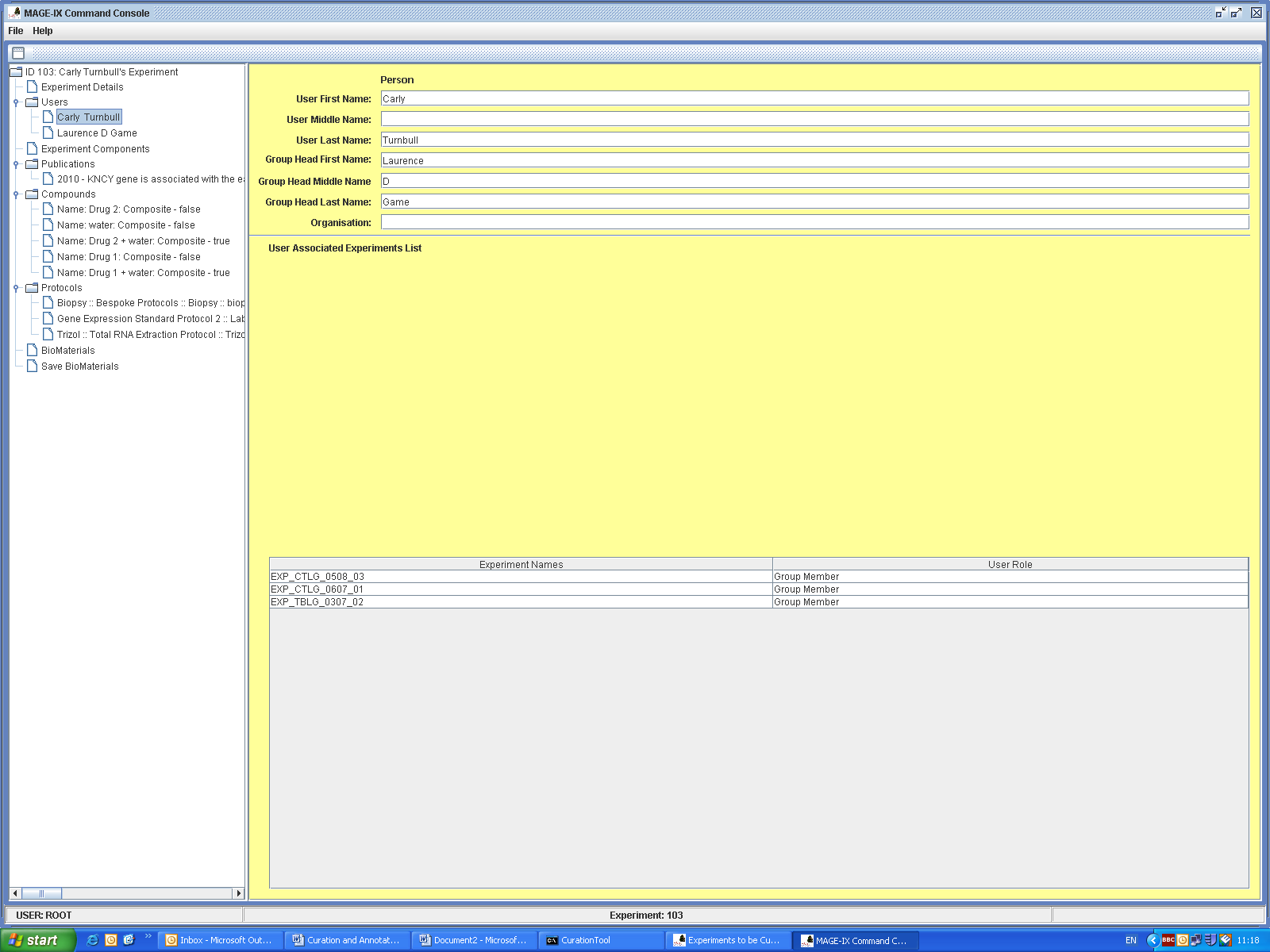


Fig. 4 (a) Screenshot of Users module in the experiment window showing the record for an existing user.

1. New Users:

If a user does not currently exist in MiMiR, the page will appear in blue with a ‘New Person’ flag at the top of the window (Fig. 4b).

To check whether a new user already exists in MiMiR, go to the Experiment Components module. Under the Users tab, inspect the dropdown list for any existing record for this user. If the user is present in the list then a new record is not needed. If there is no record for this user, go back to the Users module and save the new user record. The user contact details and affiliation should, if necessary, be edited before saving the new record.

It is also possible to create a record for any other new user at this point. This can be done by placing the mouse over the Users module in the left-hand panel, right-clicking and selecting New User. All new users should have records created before moving to the next module.


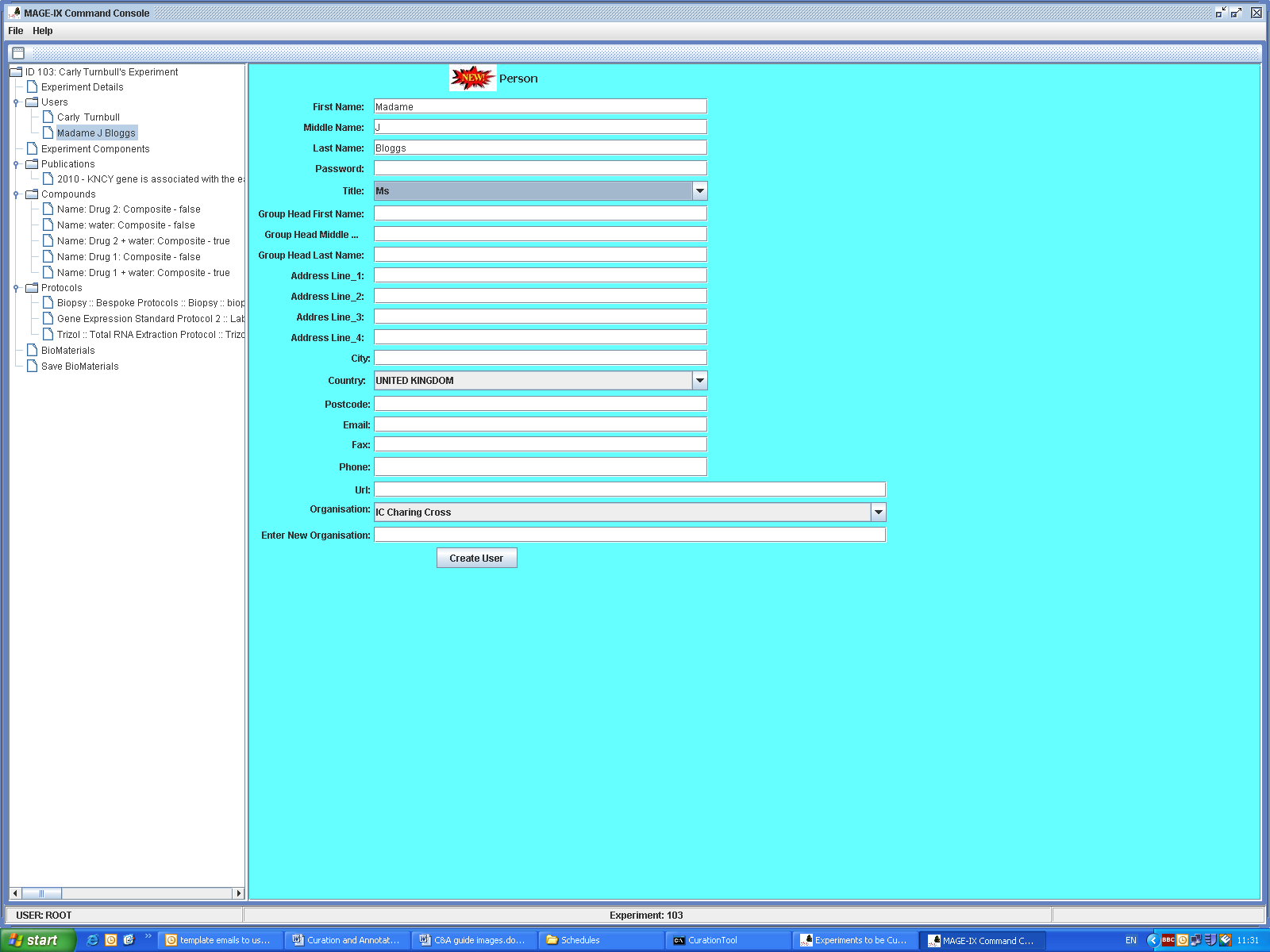
Fig. 4b Screenshot of Users module showing the record for a new user.

1. **Experiment Components module**

This module contains the Experiment Design, Experiment Factors and Experiment Users tabs.

1. Experiment Design tab:

This tab contains details of the aims of the experiment that are described using the MGED ontology (MO) (Fig. 6). Clicking on the Ontology button at the top of the page opens up the MGED Ontology Viewer (MOV) displaying the ontology tree (Fig. 7). The most suitable MO term is selected by double-clicking the appropriate entity in the tree. The ExperimentalDesignType branch is pre-selected in the viewer to facilitate the choice of the appropriate term to be assigned. The MO: prefix is assigned to all terms selected from the MGED Ontology Viewer.


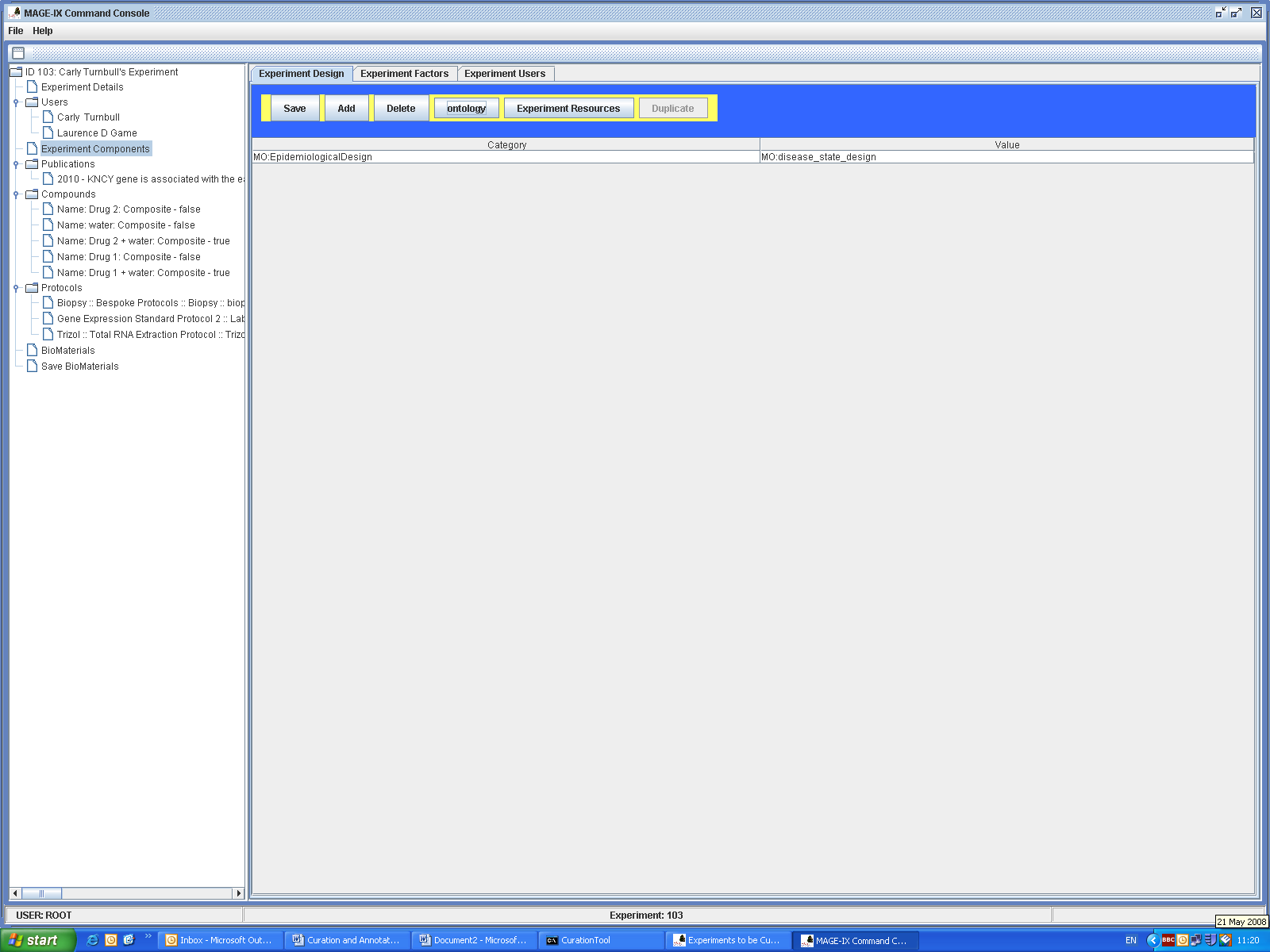


Fig. 6 Screenshot of the Experiment Design tab in the Experiment Component module.


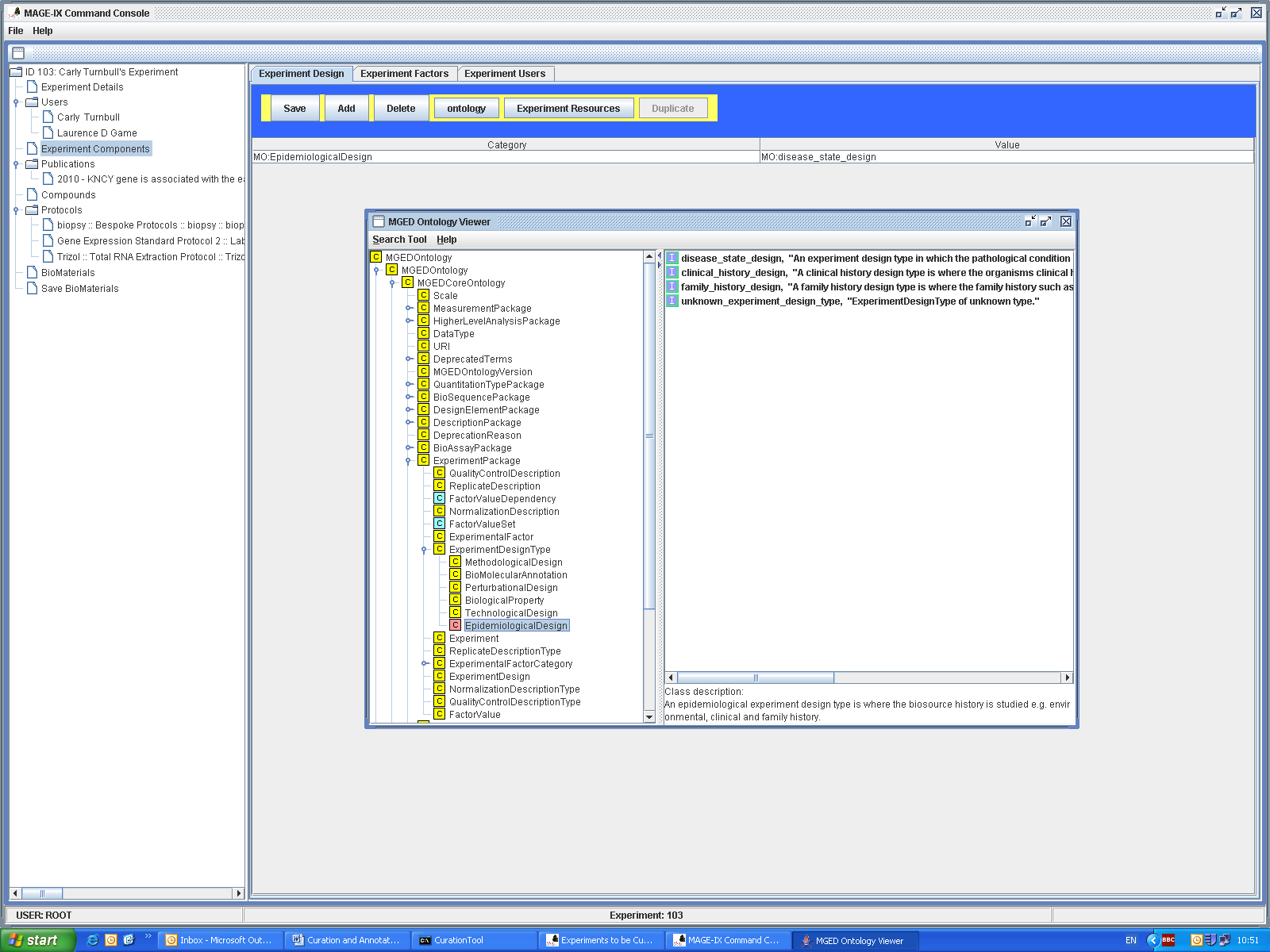


Fig. 7 The MGED Ontology Viewer.

Double-clicking on any term in the ontology tree will populate a field with the selected ontology entry. A selected term from the right-hand panel of the tree will be inserted in the FactorValue box of the ontology entry, and the FactorCategory field will also be automatically populated with the correct term. When there are no terms in the right-hand panel of the ontology tree then the term selected by the curator will populate the FactorCategory box, while the FactorValue box will be left blank.

Note that once the experiment design type has been selected and saved in the MIMIR database, it can no longer be edited during the curation process. An experiment can be assigned more than one design type, but all design types have to be assigned before saving.

1. Experiment Resources tab:

An Experiment Resources tab is available in both the Experiment Design and the Experiment Factors tabs. These tabs provide information as originally entered by the user in the Online Annotation Tool and help curators to select the most appropriate experimental design and factor MO terms. It details the treatment groups, aims, description and any compounds used, as well as the protocols specific to this experiment (the bespoke protocols).


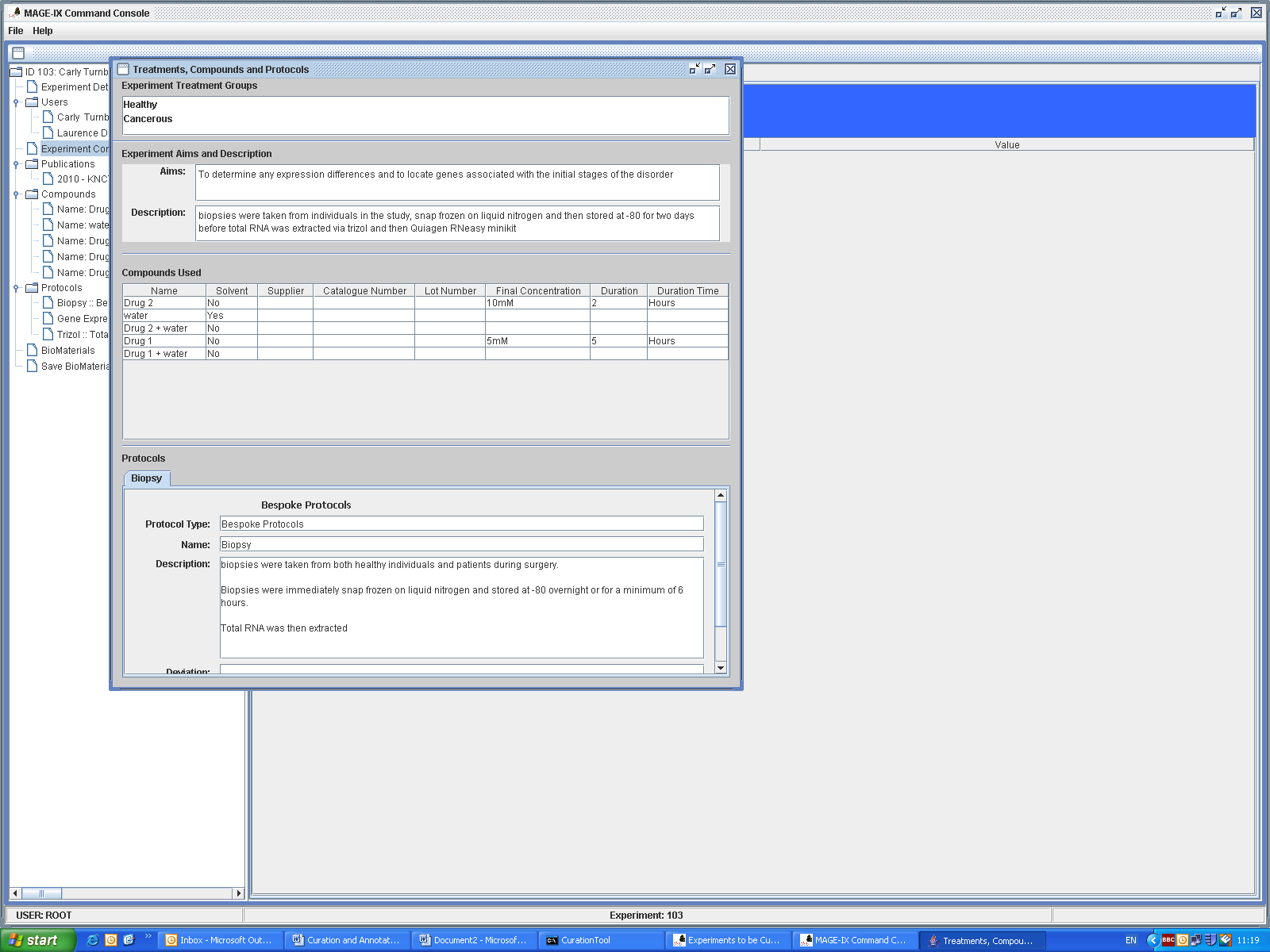


Fig. 8 Close-up of Experiment Resources window.

1. Experiment Factors tab:

This tab contains details of the factors, or variables, of the experiment that are described using MGED ontology terms (Fig. 9). In the MOV, the ExperimentalFactorCategory branch is automatically displayed and terms from this part of the tree can be assigned. NCI Metathesaurus terms and corresponding Accession IDs can be entered when an appropriate MO term is not present. This information should be entered into the FactorValue field, with the Accession ID entered into the corresponding Accession ID field. NCI terms are assigned the prefix NCI: and the spelling should match exactly the one listed in the NCI Metathesaurus browser (http://ncimeta.nci.nih.gov/MetaServlet/)

Each row corresponds to one factor. The FactorName field is a free-text field that is populated in a standardised way by trained annotators to ensure consistency in annotation. Note that once the factors have been entered and the information is saved, it can no longer be edited during the rest of the curation process. All factors should therefore be assigned to the experiment before saving.

A number of factor groups can be assigned, depending upon the aims of the experiment. The factor groups are added in brackets after the FactorCategory term to distinguish between the factor groups (see example below).

**FactorCategory FactorValue FactorName**

MO:ComplexAction(CompoundY) MO:compound_treatment CompoundY_0

MO:ComplexAction(CompoundY) MO:compound_treatment CompoundY_10

MO:ComplexAction(CompoundX) MO:compound_treatment CompoundX_0

MO:ComplexAction(CompoundX) MO:compound_treatment CompoundX_5

This distinction between similar factor categories is also necessary for the creation of an ‘Experiment Definition’ if the information is exported to Rosetta Resolver.


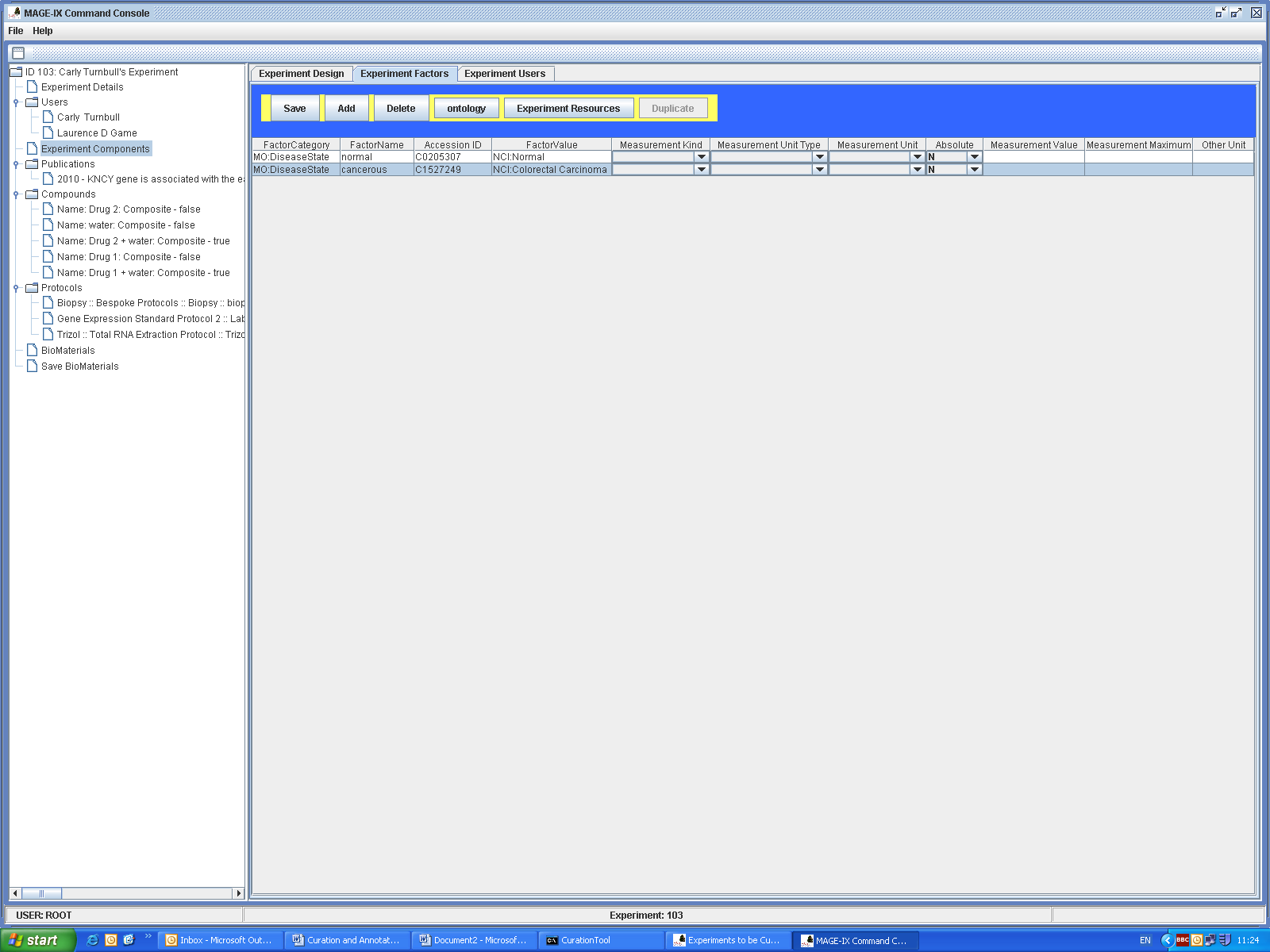


Fig. 9 Screenshot of the Experiment Factors tab of the Experiment Components module.

1. Experiment Users tab:

The user records present in the Users module will be displayed in this tab. Any new or existing user can be selected from the drop-down list, be added to this experiment and their roles can be assigned (Fig. 10). Additional existing users can be linked to the experiment at this stage. This can be done by clicking on the ‘new’ button to add a new record and then selecting the appropriate name from the drop-down list. Once saved, the fields become locked for editing and any further changes can be done using the Annotation Tool.


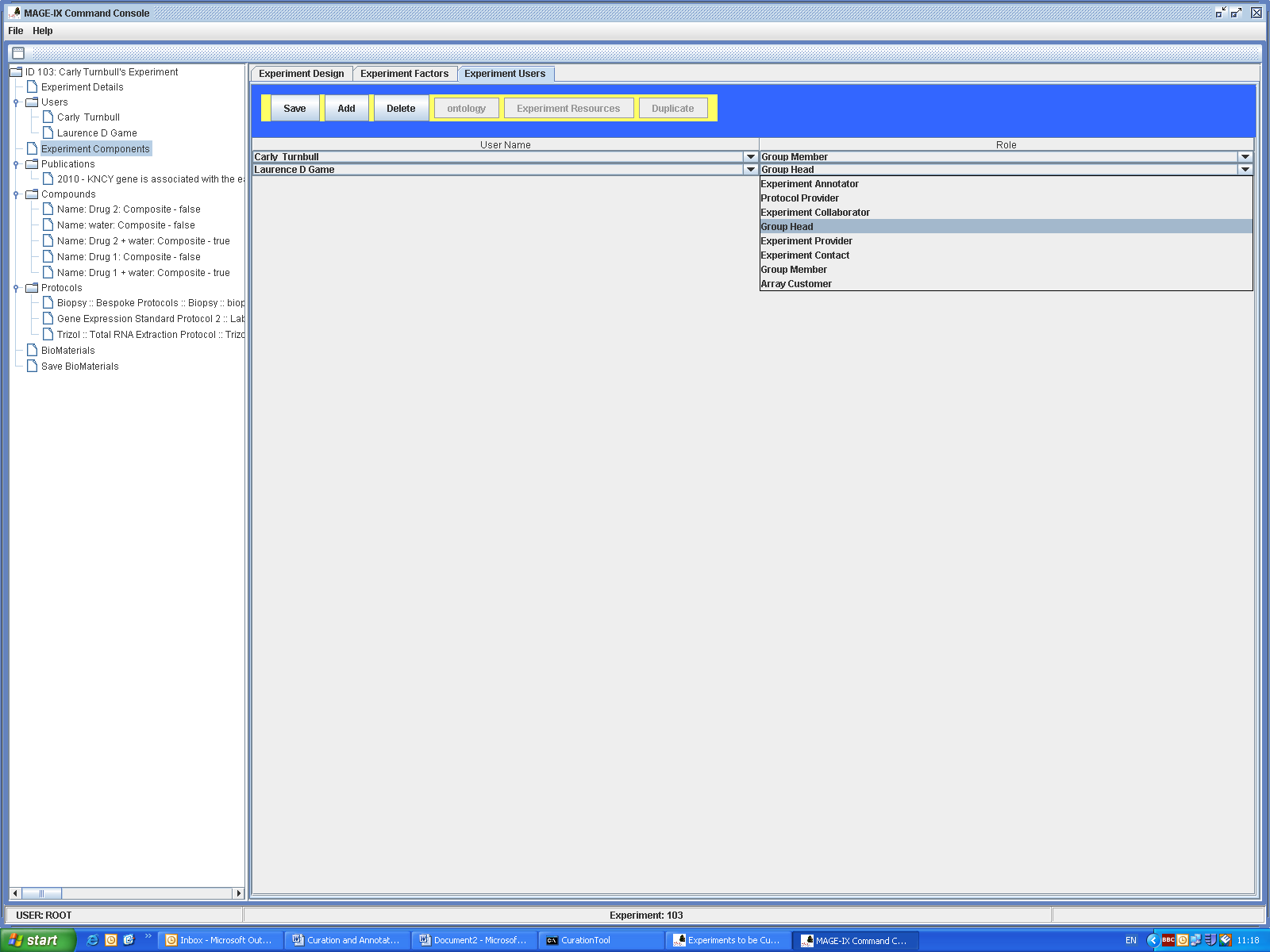


Fig. 10 Screenshot of the Experiment Users tab in the Experiment Components module.

1. **Publications module**

Publications referenced by the user in the Online Annotation Tool are automatically populated in the Curation Tool. The Entrez PubMed ID is displayed and the abstract can be accessed via the ‘View Abstract’ button. All fields can be edited if necessary. New or additional publications can be entered by selecting the Publications module title in the left-hand panel, right-clicking and selecting ‘new publication’. It is also possible to delete publications.

The type of publication (online resources, books or book chapters or journal articles) and the reference format are determined by the type of publication selected by the user in the Online Annotation Tool.


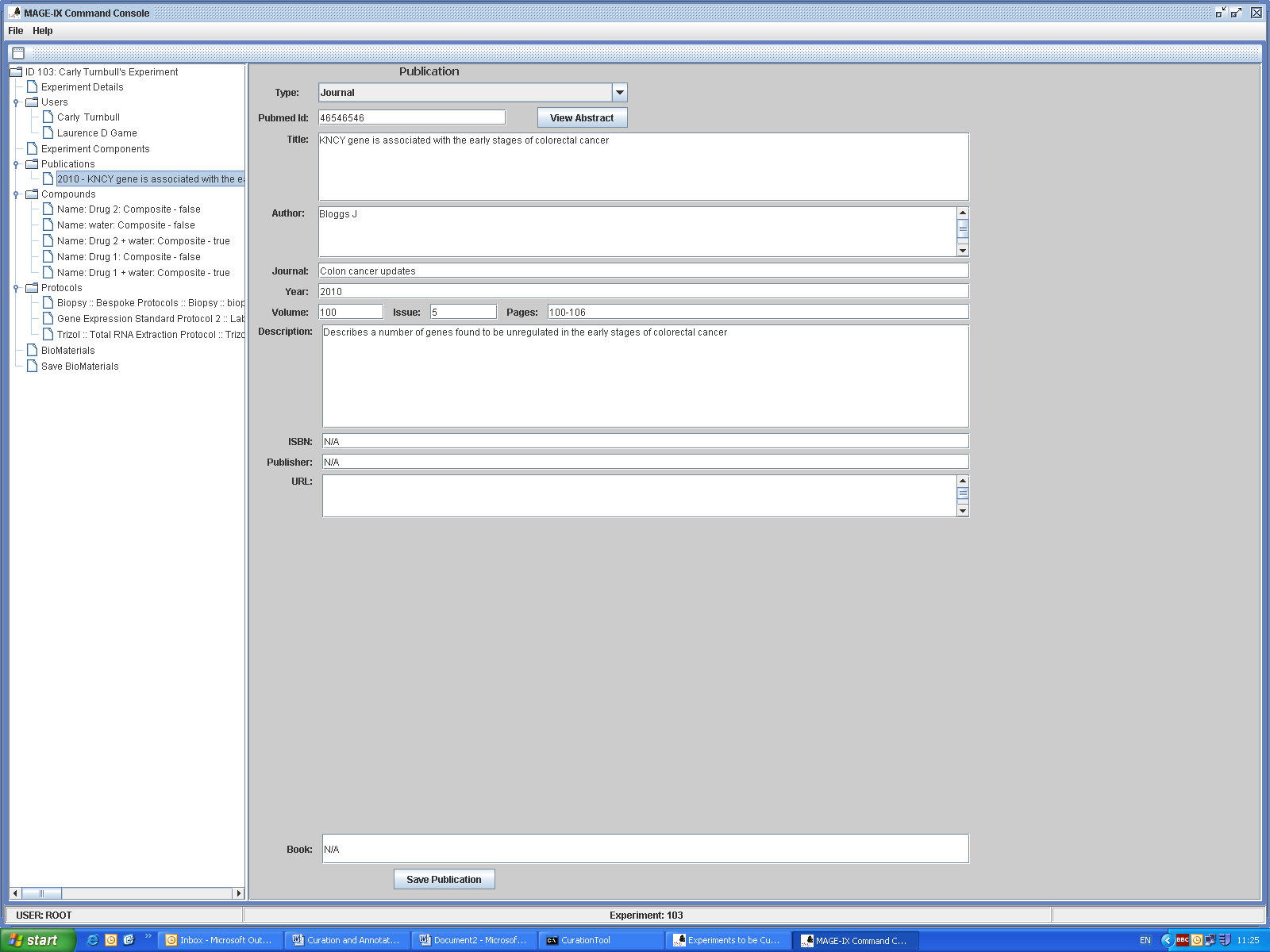


Fig. 11 Screenshot of the Publications module.

1. **Compound module**
2. Component Compounds

These are the individual components that make up the final compound, or composite compound, with which the biosamples are treated. The Curation Tool automatically searches for similar existing compounds before a new compound is created. The curator can also manually search through all of the existing compounds to look for a match (Fig. 12). If no record is found, a new one can be created. Some curation is often required at this stage to follow naming conventions rather than using names provided by users.

The new and existing compound records are colour-coded: existing compounds are selected using the red “old compound” button, while new compounds are selected using the grey button.


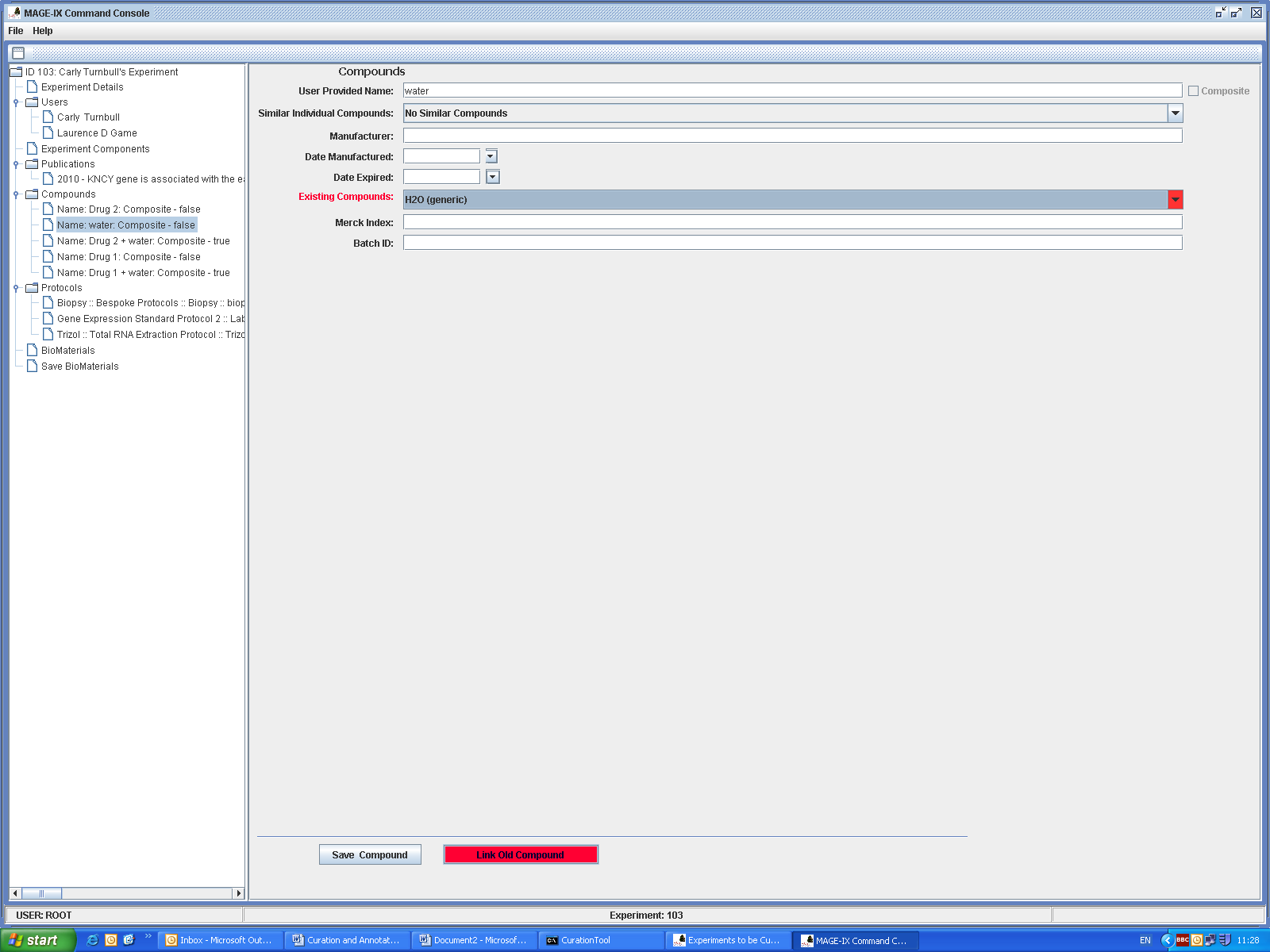

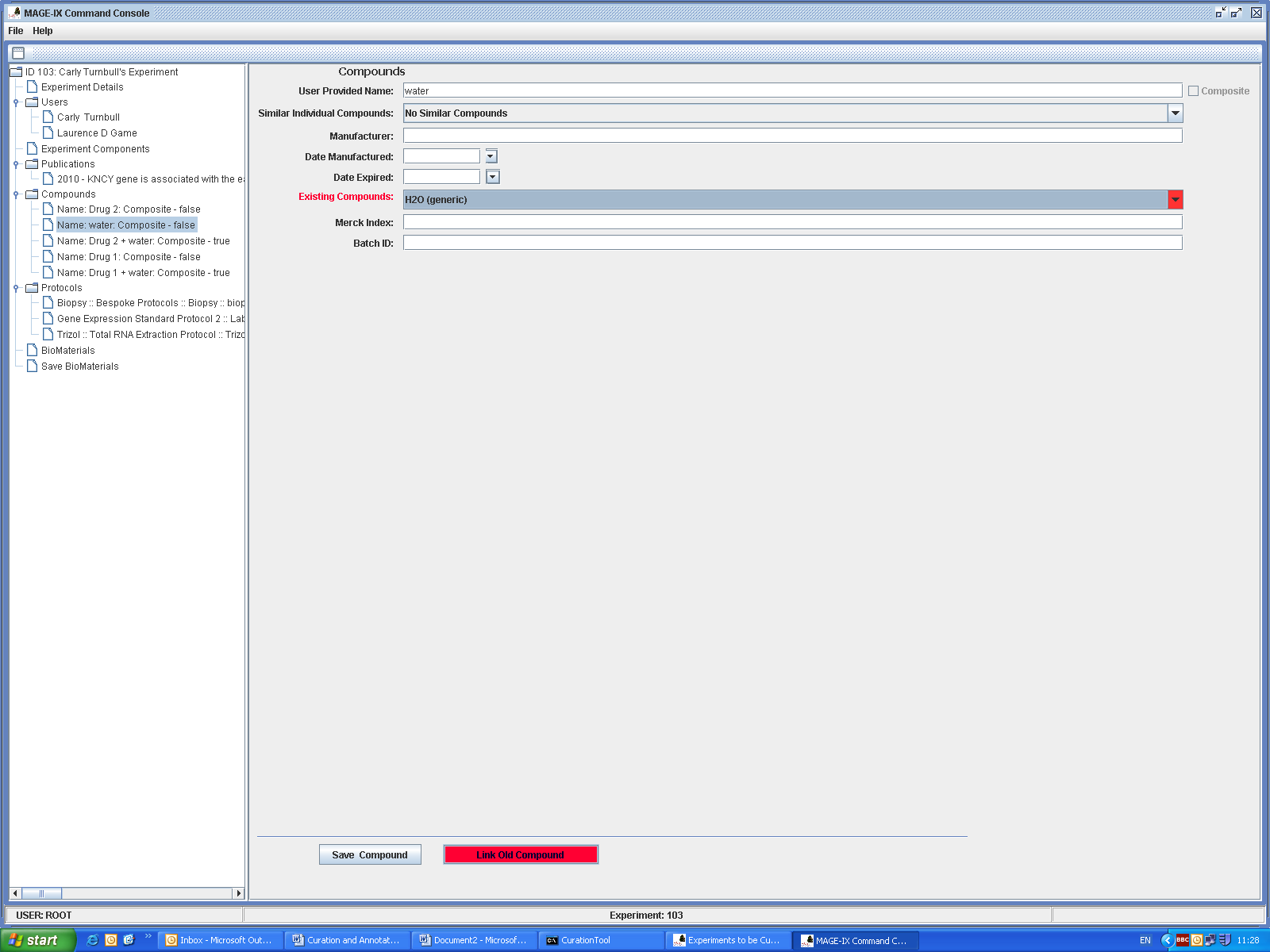

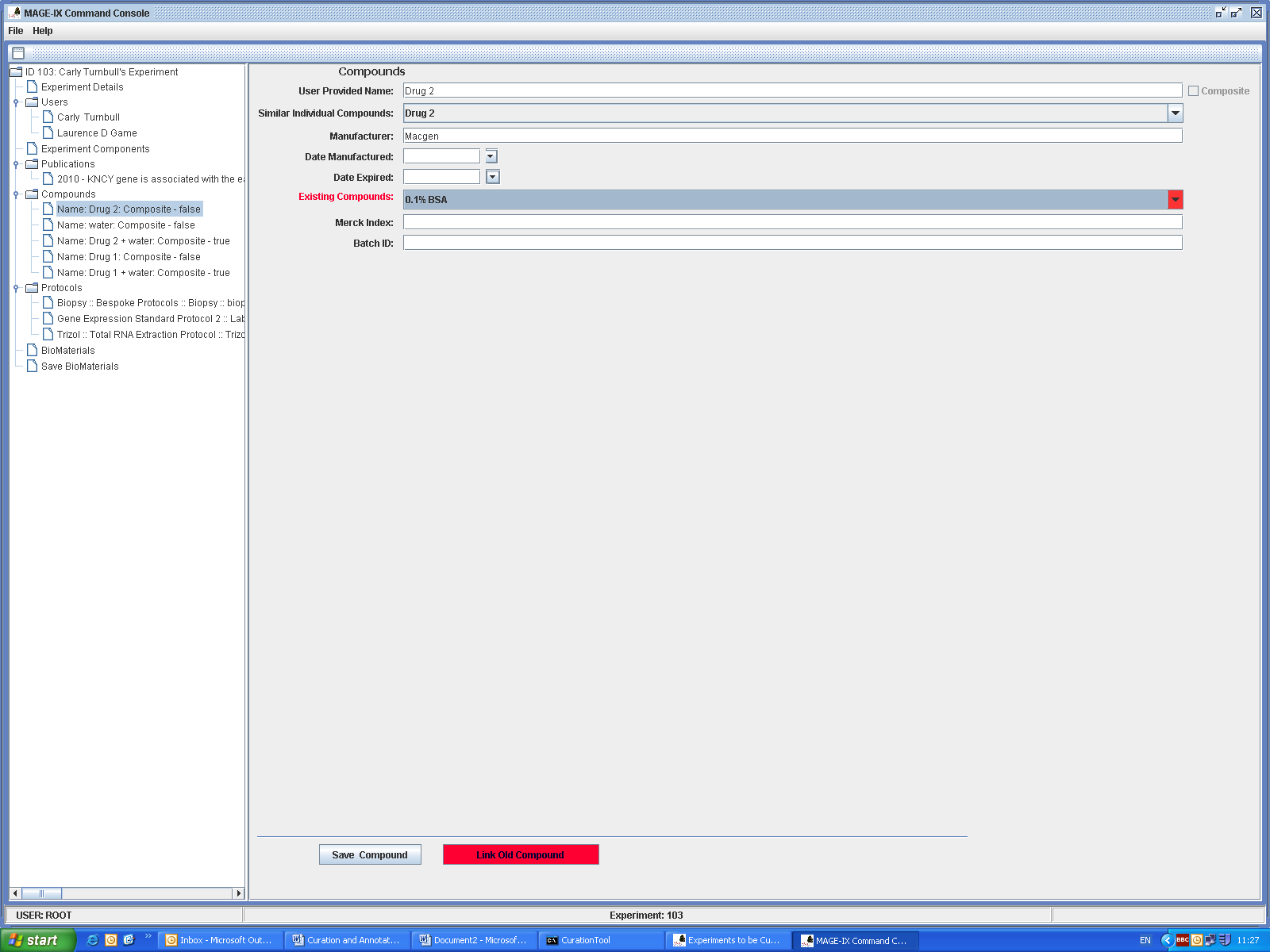

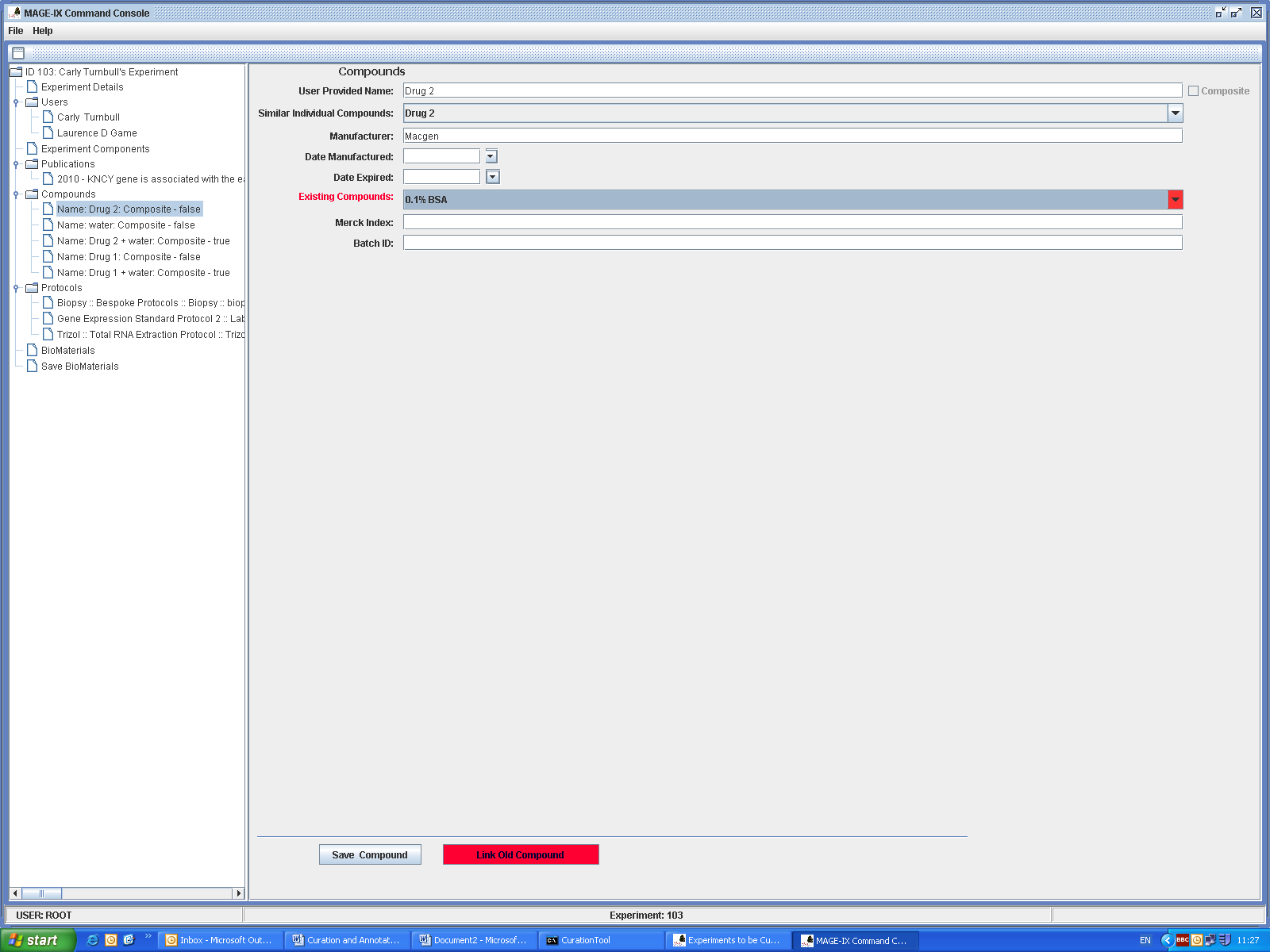


Fig. 12 Screenshots showing the two Component Compounds.

All the component compounds necessary to make a composite compound have to be created and saved before the composite compound record can be curated.

1. Composite Compounds

The names for the composite compounds are recorded by listing the first compound “+”the second compound. The component compounds should be automatically listed in the bottom of the right-hand panel (Fig. 13). Other compounds can be added or deleted at this stage.

Any measurement details recorded here should be for the component compounds rather than the composite compounds. For example: Drug 2, at a 50µM concentration, was added to 5µl of H_2_O to give a final concentration of 5µM. The measurements of the compounds are listed here but the final concentration should not be entered here (Fig. 13). Generally it is better to give details of volumes rather than concentrations to avoid any confusion.

Again the curator has the option of saving the new composite compound or linking the experiment to an existing composite. Some curation is often required at this stage to follow naming conventions. Saving this page will save the composite compounds, along with the links to the component compounds. If new compound records are needed they can be added by selecting the Compounds module title in the left-hand panel, right-clicking and selecting the appropriate type of compound.


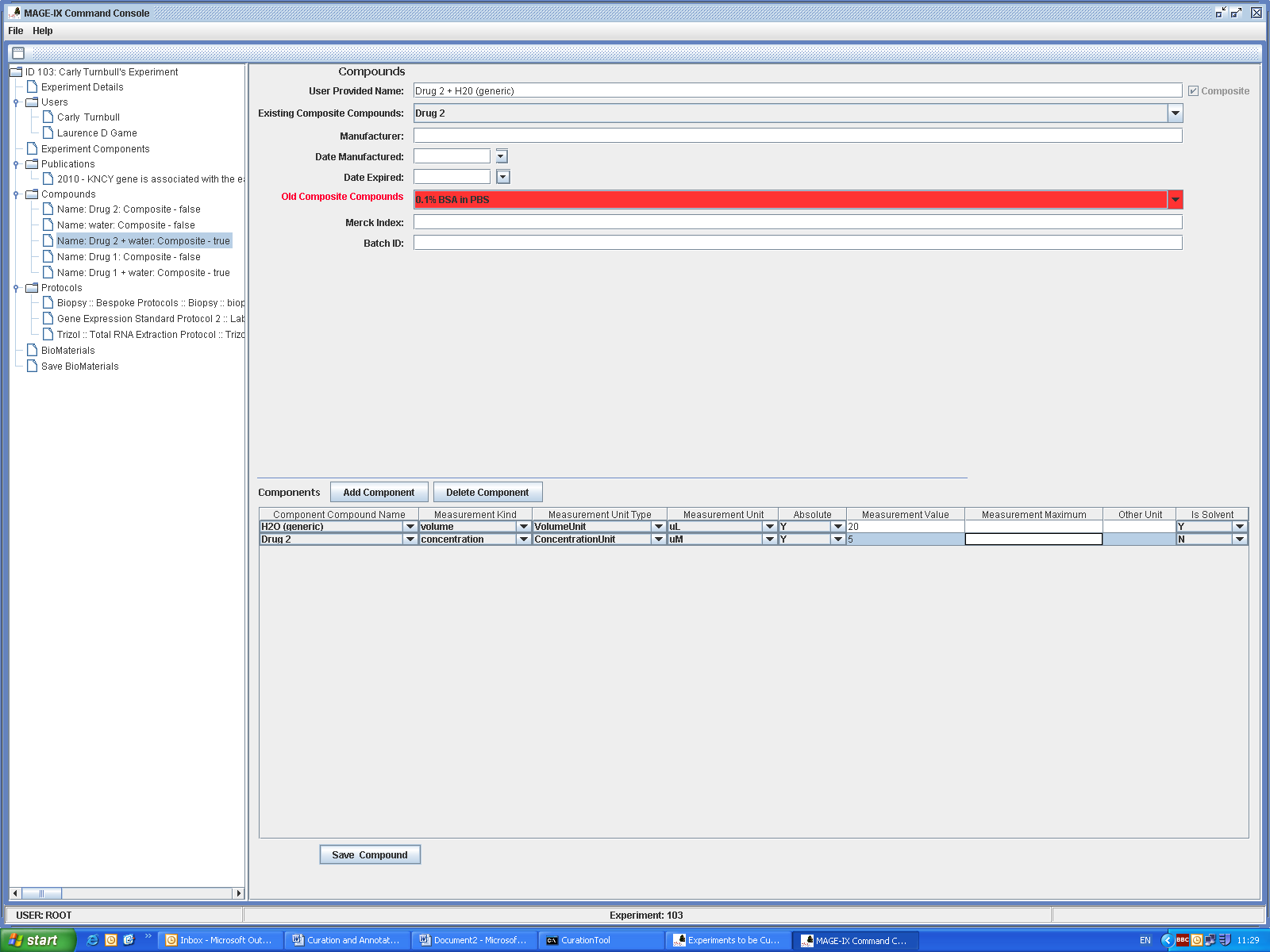


Fig. 13 Screenshot showing a Composite Compound record.

1. **Protocols Module**

This module is split into 3 sections (Bespoke, Total RNA extraction and Labelling protocols) depending on where the protocol details were entered in the Online Annotation Tool. Each section will display the relevant protocol which can be saved or amended in a similar way. There is the option to create new protocols or to link existing protocols (protocols which already exist in the MiMiR database) to the experiment. Each protocol is automatically assigned a provider which can be a company (e.g. for a commercial kit) or an individual (e.g. provided by a user). All Microarray Centre protocols should have the Microarray Centre listed as the provider.

The Stage (e.g. treatment, total RNA, labelled extract) is the form that the sample is in when a treatment has been completed, and this is automatically generated by the Curation Tool. If the user has deviated from any of the protocols during the experimental design, this will be listed in the Protocol Deviations section. These deviations would apply to all of the samples in the experiment, so this information, once saved, will be automatically linked to the application record for this protocol in all the relevant biosamples. If a protocol deviation has only occurred for one, or a small number of samples then this information can be added to the protocol application record using the Annotation Tool on a sample-by-sample basis. In this case the curator would remove the deviation information in the Curation Tool before saving the protocol record.

1. Bespoke protocols

Bespoke protocols describe treatment steps prior to total RNA extraction and tend to be specific to each experiment. When entering protocols in the Curation Tool the curator has two options; create a new protocol for the user or select an existing protocol and link it to the experiment at this stage (Fig. 14). On opening the bespoke protocol page, the curator can search for existing protocols using the user and group head initials followed by an asterisk (Fig. 14). Alternatively the curator can manually view all existing records by entering an asterisk in the search field. The curator can select an existing protocol from the drop-down list and check the description. For new protocols the title and description often need to be edited for consistency.

The Curation Tool automatically adds the user and group head initials before the protocol title to make the unique name of the new protocol. As with most of the other modules in the Curation Tool, existing protocol descriptions are coloured in red, the same as the ‘link old protocol’ button, to distinguish old and new protocols.


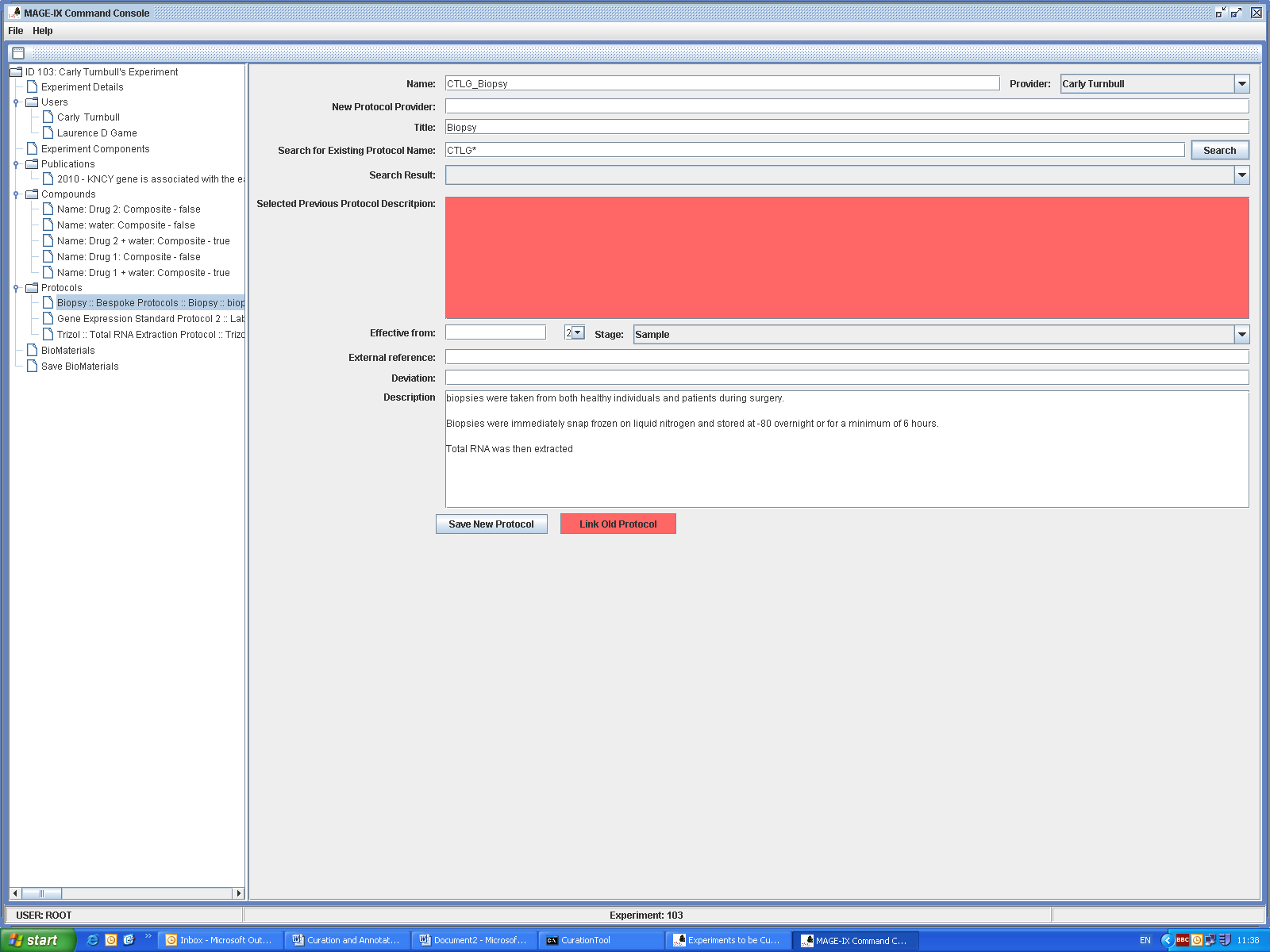


Fig. 14 Screenshot showing a Bespoke Protocol from the Protocols module.

1. Total RNA extraction protocols

The extraction protocols page is split into the top section for extraction protocols and the bottom section for the cleanup protocols (Fig. 15).

Only one protocol can be assigned to each treatment step except for the total RNA extraction. Extraction protocols are entered by the user as free text and curators can select the appropriate existing protocol from a drop-down list. As with the bespoke protocols one can create new protocols or link existing protocols, but the majority of protocols used will already exist in the database. New protocols will be given the prefix ‘user and group head initials’ to make it specific. Descriptions for new protocols are requested from users and this information is currently added by the data warehousing team.

Similarly, cleanup protocols are entered and can be curated in the bottom section. The RNeasy mini kit is the only clean up protocol listed so far.

The extraction protocols page will be subject to changes to accommodate new microarray applications. The extraction and cleanup protocol lists are designed to be dynamic and new protocols can be added to the existing lists.

Deviations from existing protocols can be captured in the Online Annotation Tool and are added to the corresponding protocol application record in the Curation Tool for both the extraction and cleanup protocols. The details are checked and assigned accordingly.


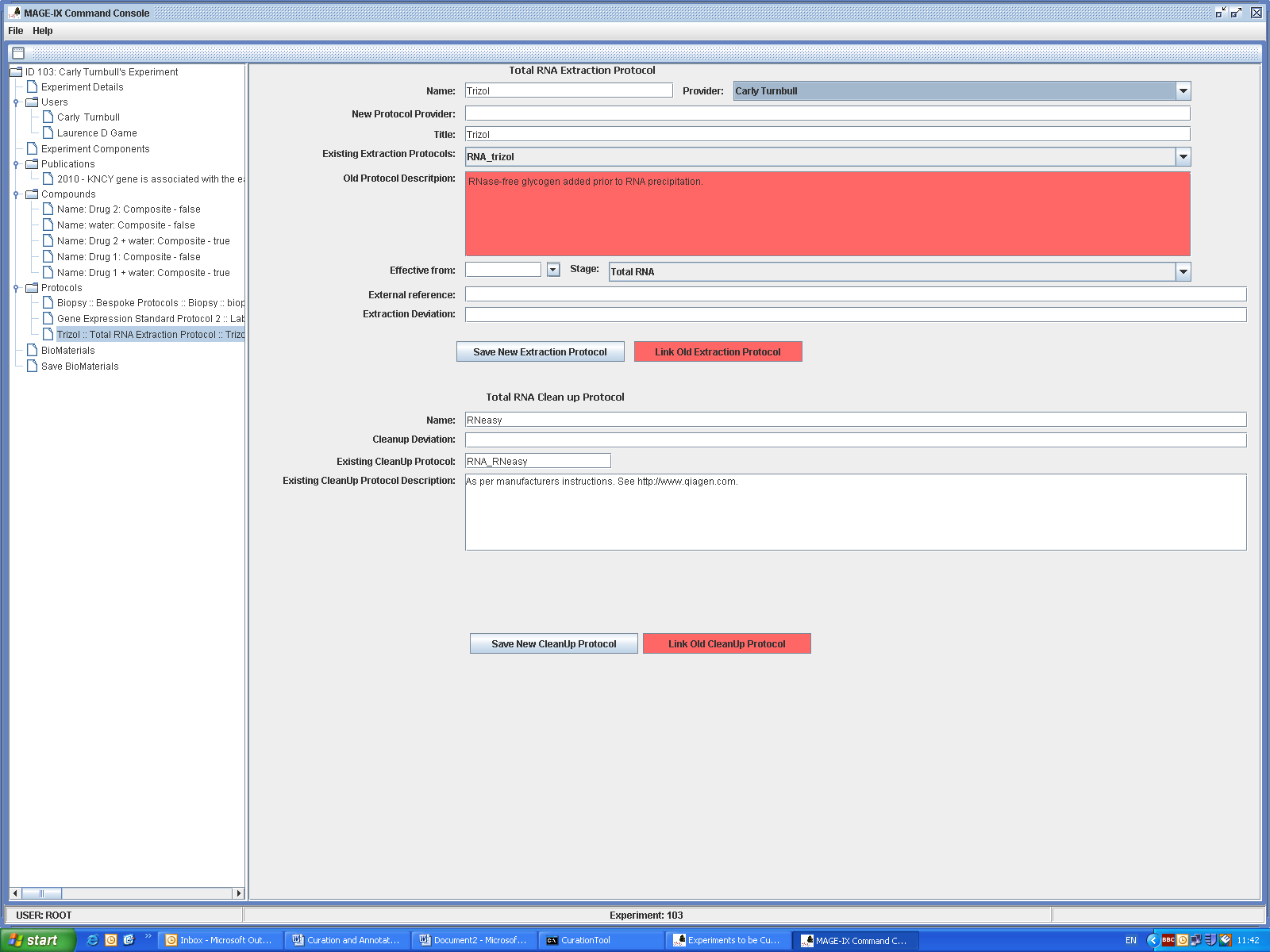


Fig. 15 Screenshot showing an Extraction and a Cleanup Protocol from the Protocols module.

1. Labelling protocols

Existing Microarray Centre labelling protocols have records in the database and the protocol selected by the user is displayed (Fig. 16). A number of downstream treatment steps and biosamples are created by the Curation Tool based on the labelling protocol and the array type selected by the user. For example, the Whole Transcript 100ng Labelling Protocol can be used with Gene 1.0 ST or Exon 1.0 ST arrays. The tool is designed to take this into account as it will recognise the array type and will build the hybridisation step, attaching the appropriate hybridisation cocktail protocol.


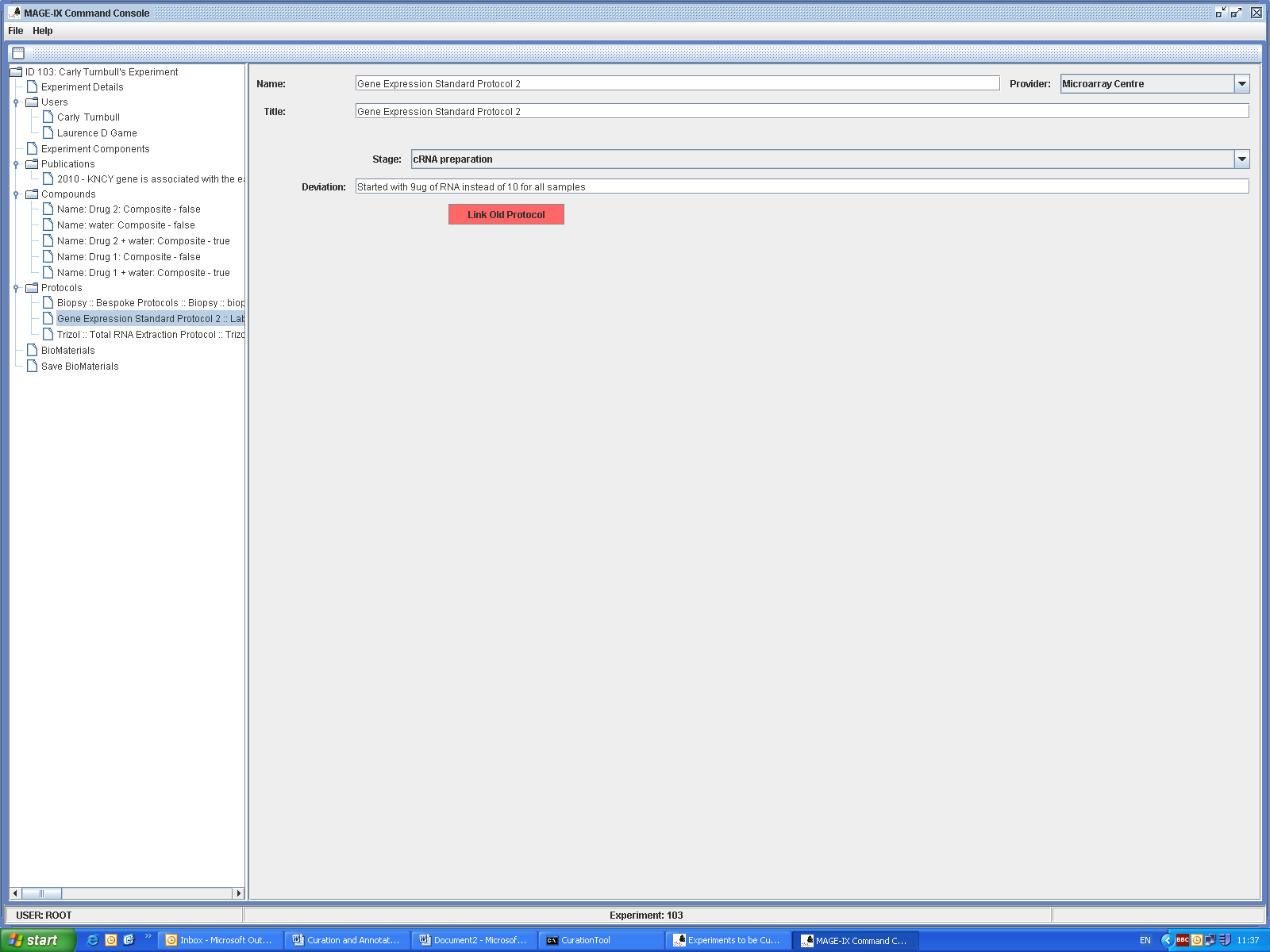


Fig. 16 Screenshot showing a Labelling Protocol from the Protocols module.

1. **Biomaterials module**

Based on the information given for each protocol, the Curation Tool automatically generates a graphical view of the biomaterials for the experiment (Fig. 17). In this graphical view nodes represent biomaterials and arcs represent treatment steps. The biosources (with a prefix BS_) are the organisms included in the experiment. The biosamples (with a prefix BSM_) are the samples derived from the organisms and that are treated as part of the experiment. Nodes are colour coded to represent the different stages of sample preparation (e.g. biosources are coloured orange, total RNA biosamples are coloured gold, labelled cRNA biosamples are coloured lilac etc).

It is possible to select nodes/arcs and display the corresponding information but these records are locked for editing in this tool. Biosource and Biosample names are automatically generated using the convention BS(or BSM)_user initials Group head initials_nn where nn are sequential unique numbers automatically generated (Fig. 18).


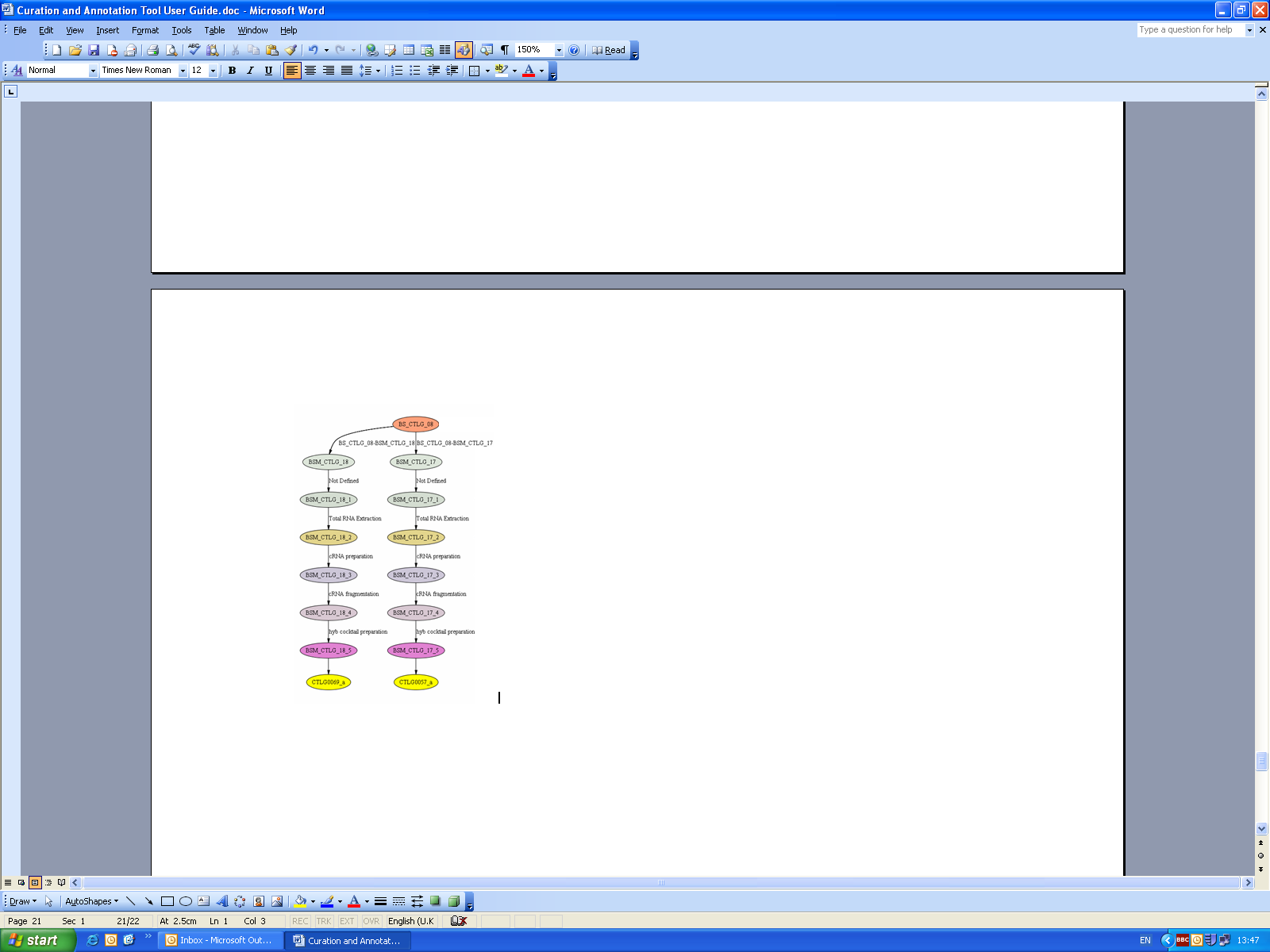


Fig 17. Graphical view of an experiment in the Curation Tool

In the Annotation Tool, the user selected: U133Plus 2.0 arrays and Gene Expression Standard Protocol 2.

- The tool leaves this step undefined and it is annotated later.
- The total RNA extraction step is created and the protocols are assigned when saved.
- Downstream steps are defined by the labelling method selected.


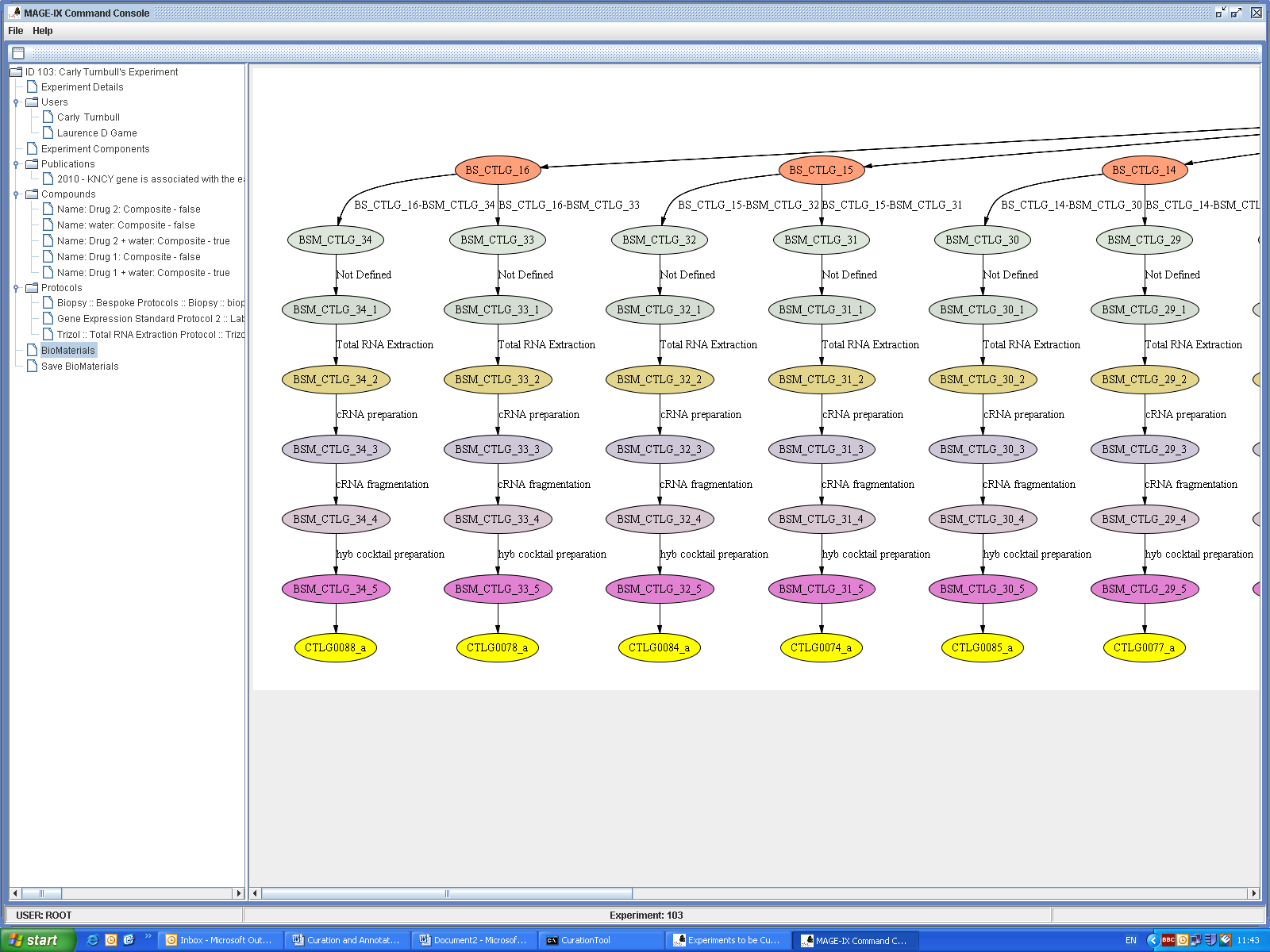


Fig. 18 Example of the biomaterials and treatment steps automatically created by the Curation Tool.

Information collected in the Online Annotation Tool is automatically linked to the relevant nodes/arcs in the graphical view of the Curation Tool (Fig. 18). Treatment steps listed as ‘Not Defined’ are specific to the experiment and will be annotated later in the Annotation Tool. Bespoke protocols are automatically created, but need to be linked to the appropriate treatment step in the Annotation Tool. Experiments involving compound treatment will have an additional compound treatment step after the undefined treatment step. The total RNA extraction step is built by the tool and the relevant protocols are linked appropriately.

All samples are assigned an aliquot number before processing. Like the other biomaterials, the aliquots are assigned a name derived from the four letter user/group head ID. This name is the four initials followed by the sequential sample number. The Cutation Tool automatically creates the Aliquot IDs and assigns them to the samples in a random order so that samples of the same treatment group are not processed together, avoiding any processing bias.

E.g. If there are 68 CTLG aliquots already present in the database then the Curation Tool will assign CTLG0069_a to the next aliquot to be created. The biosample names are created in the order they were entered into the Online Annotation Tool, so these numbers usually do not correspond with the numbers at the end of the aliquot ID. The _a indicates that this is the first aliquot created by the Microarray Centre. If the sample failed and was re-fragmented and re-hybridised then this would be classed as a new aliquot and would be called CTLG0069_b.

Once the curator has checked that the experiment graphical view is correctly built, they select the “save biomaterials” button to proceed to further annotation using the Annotation Tool.

**Part 2: Annotation Tool**

1. **Selecting an experiment to be annotated**


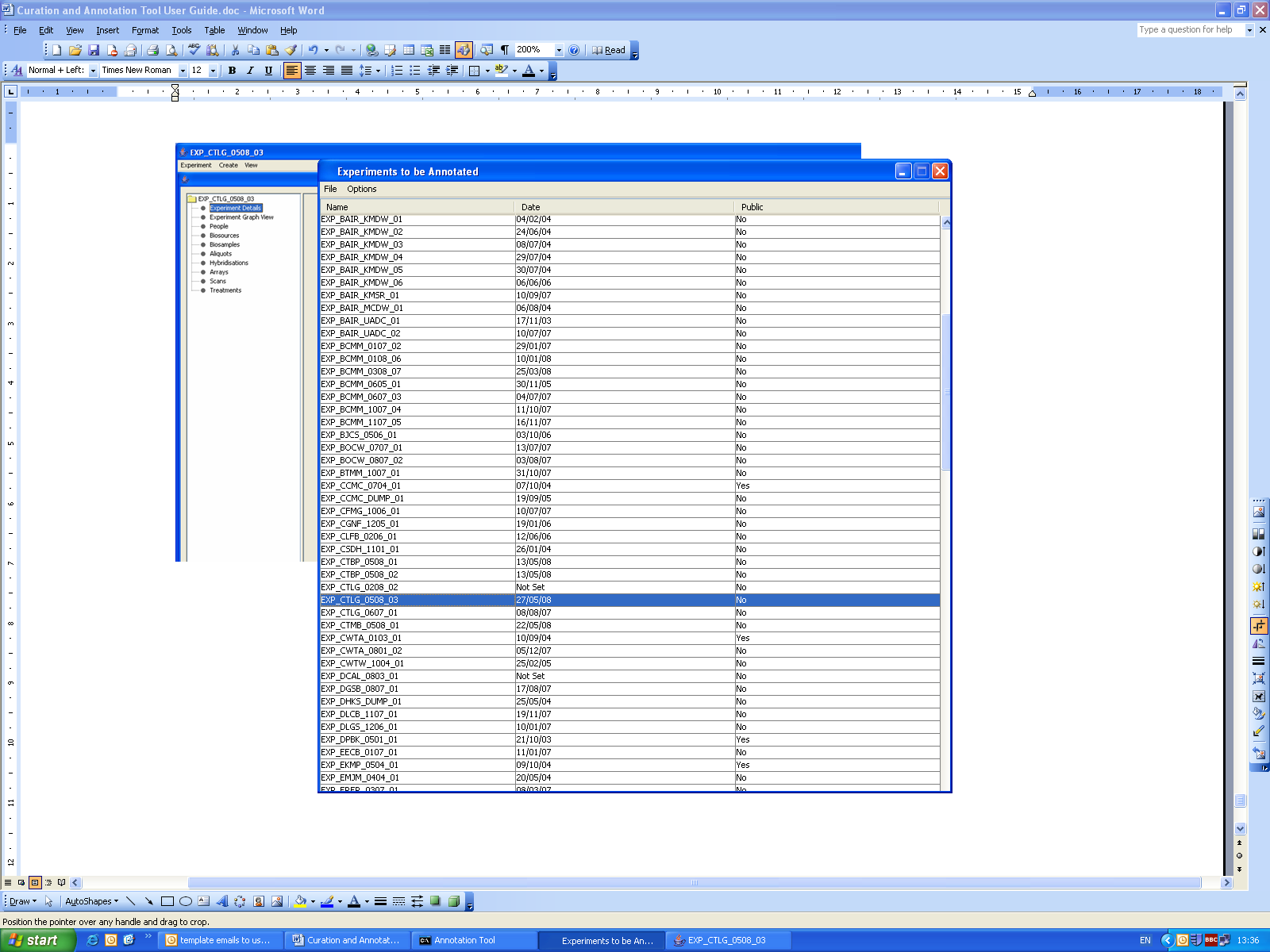
Log into the Annotation Tool and a window displaying the list of experiments to be annotated will appear. Select the experiment with the correct experiment name (Fig. 1)

Fig. 1 Screenshot of the ‘Experiments to be Annotated’ window showing the experiment name, the date of completion of curation, and the publication status.

- This window will stay open allowing access to more than one experiment at once. The Annotation Tool is designed so that an experiment can be annotated over a period of time and can be saved and returned to later.
- By selecting ‘Options’ from the top right-hand menu and selecting ‘New Experiment’ it is possible to create a new, blank experiment. This might be necessary for experiments not submitted via the Online Annotation Tool or for non-standard experiments.

1. **Experiment Window Summary**

Like the Curation Tool, an Experiment window appears on selection of an experiment. The left-hand panel of the experiment window displays the various modules of the Curation Tool (Fig. 2). The name of the experiment assigned during curation is displayed in the top left-hand corner of the screen.


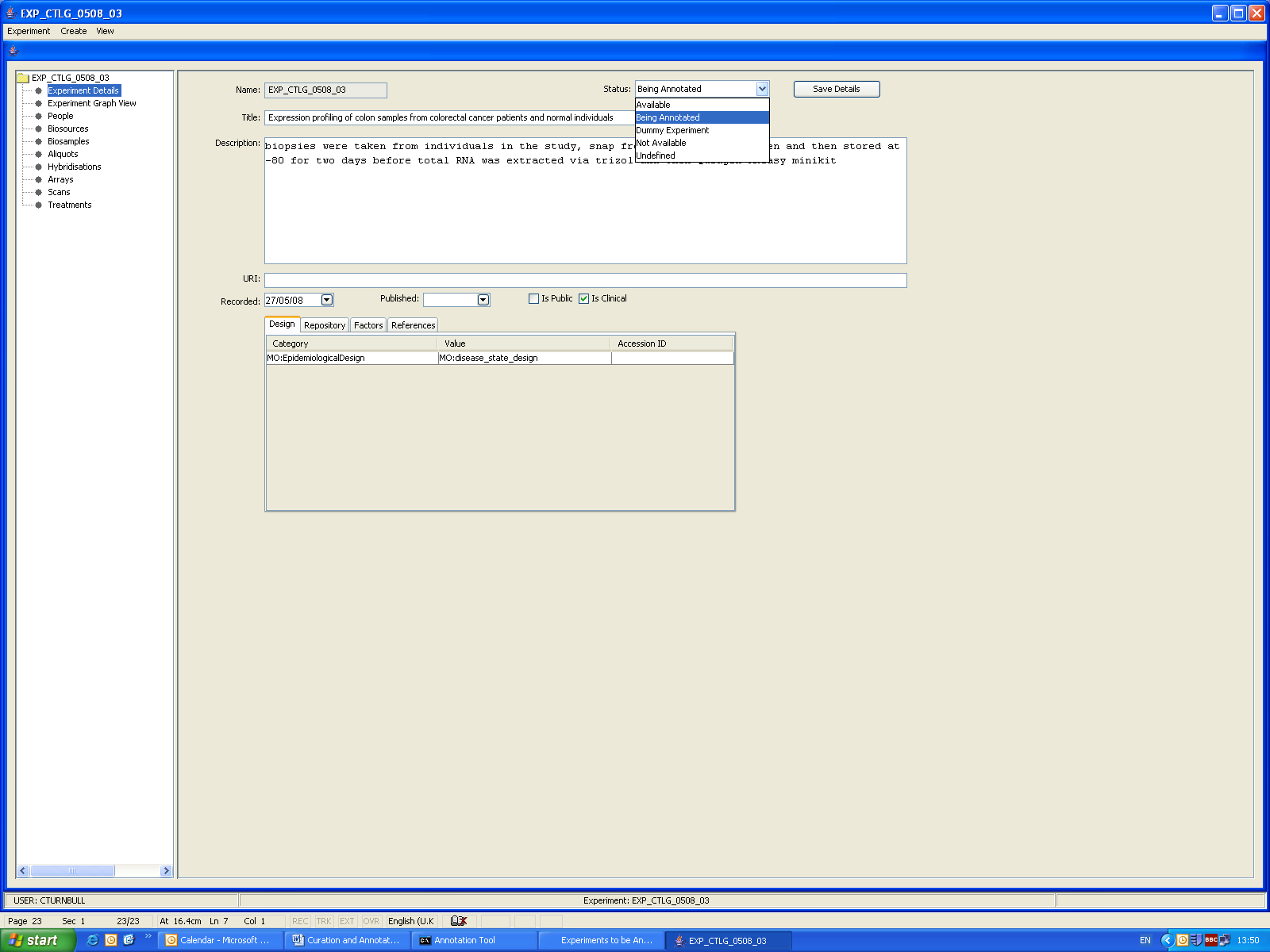


Fig. 2 Close-up of left-hand panel of Experiment window in the Annotation Tool.

The modules are:

- Experiment Details

A condensed view of the experiment details entered in the curation tool

- Experiment Graph View

A graphical representation of the biomaterials and treatments created in the Curation Tool

- People

The users linked to this experiment and the roles they have been assigned

- Biosources

The organisms used in this experiment

- Biosamples

The biomaterials treated or investigated during this experiment

- Aliquots

Details of the aliquots hybridised to the arrays

- Hybridisations

Details of the hybridisations

- Arrays

Details of the arrays hybridised

- Scans

Details of the scans and the data files

- Treatments

Details of the biosamples and how they are treated at each stage of the experiment

1. **General Functions**

- Saving to the database

The annotator should regularly save any changes to the database and reload the experiment to ensure that no information is lost. This can be done by clicking on the experiment menu from the top left corner of the experiment window and selecting ‘Save experiment to MDSA and reload’. Under this menu there are a number of other options such as reloading the experiment, refreshing the graph or saving the experiment to the database without reloading it in the annotation tool. The tool will first save the experiment to the database and then reload the graph.

Pop-up boxes are used to view information in a number of modules. When these boxes are edited they should be saved before being closed. The ‘save’ buttons save the data to the local computer temporarily, and the whole experiment needs to be saved to the MiMiR database before exiting.

- Creating new protocols/compounds

A ‘New compound’ or ‘New protocol’ can be created by selecting them from the Create menu at the top left of the Experiment window. For both compounds and protocols a new record will appear and all the details of these records should be entered before saving. For compound records there is the option to link component compounds, if the compound is a composite compound. All component compounds should be created before the composite compound.

- Viewing protocols/compounds

Check the existing protocols and compounds before creating new ones. This can be done by selecting the appropriate option from the View menu in the top left of the Experiment window.

1. **Experiment Details**

The first module in the Annotation Tool is Experiment Details. It contains a condensed view of the information taken from the Curation Tool as well as a status drop-down box, which shows the current annotation status of the selected experiment (Fig. 3).


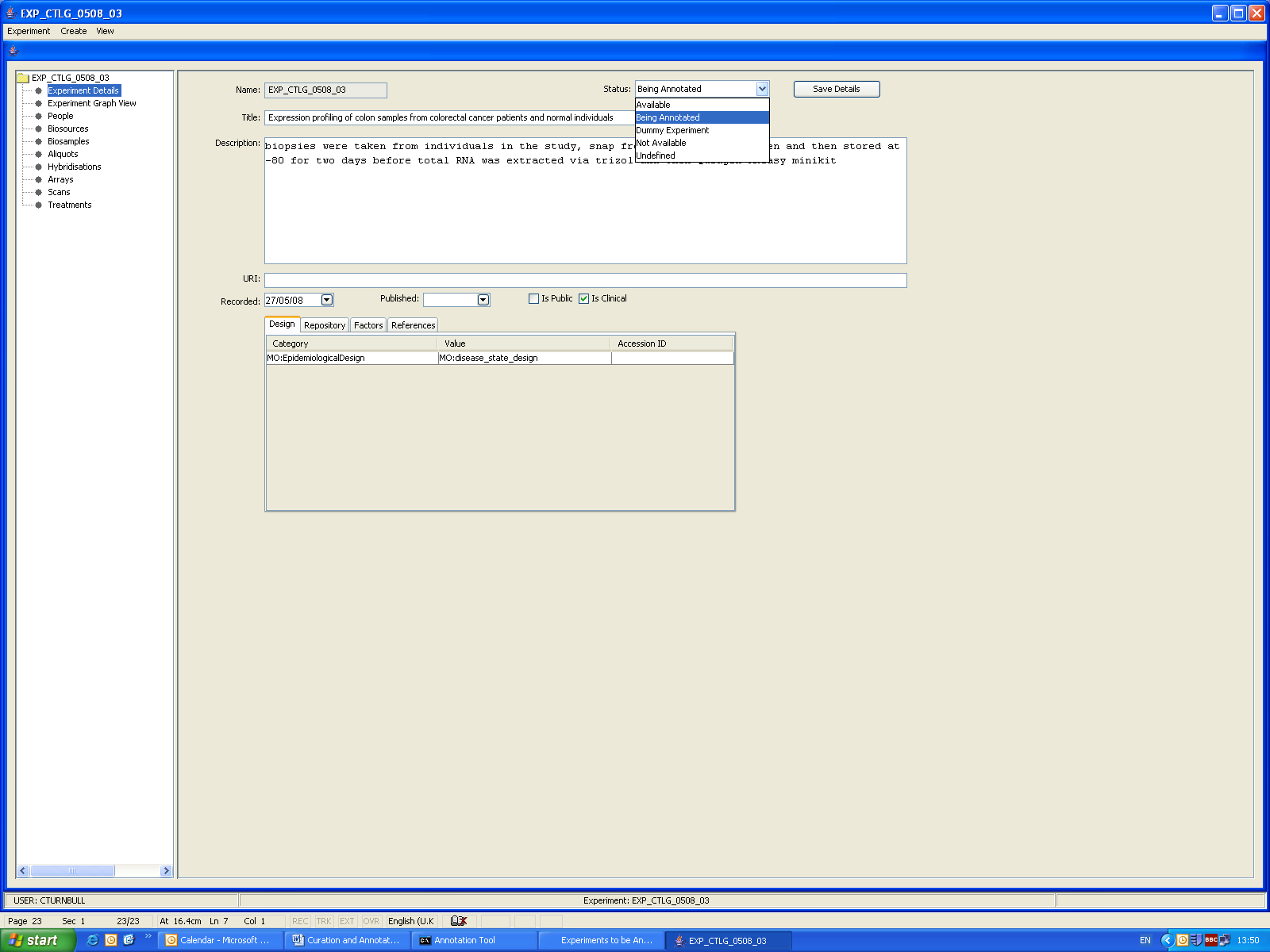


Fig. 3 Screenshot of the Experiment Details window

Opening an experiment in the Annotation Tool for the first time changes the status to ‘Being Annotated’.

The majority of the information on this page should already be populated. There are a number of tabs displayed in the middle of the right-hand panel of this page:

1. Design tab:

To edit the design type select the ontology entry, right-click and select ‘Edit’. It is also possible to select ‘Delete’ from the same menu (Fig. 3). To add a new entry, right-click on the column headers and select ‘Add’. The MGED Ontology Viewer will open and a new ontology term can be selected. As with the Curation Tool the ontology tree is limited so that only terms from the correct branch can be selected.

1. Repository tab:

This contains details of when the experimental information has been sent to ArrayExpress for publication (Fig. 4). This box will usually be empty during the annotation stage, but can be edited later. When adding a record the Array Express Accession ID should be entered, along with the date submitted and the date released.

1. Factors tab:

The factors are divided into factor groups. In the Factors tab it is not currently possible to view the factor values, accession IDs or measurements, but this functionality is being added. In the meantime, these can be altered through the back end by the data warehousing team. Right-clicking on the existing factor group name allows you to ‘add new factor group’ or to add new factor’. New factor groups should be created first, and the relevant factors should then be assigned to these groups (Fig. 3).

1. References tab:

This displays the publications entered in the Curation Tool (Fig. 3). As with the Design tab it is possible to edit or delete a reference by right-clicking on the record; a new record can be created by right-clicking on the ontology column header and selecting ‘Add’. Based on the information gathered from the Online Annotation Tool, there are a number of record templates available depending on the type of record selected. This information may have been curated, but references can still be edited or added in the Annotation Tool.


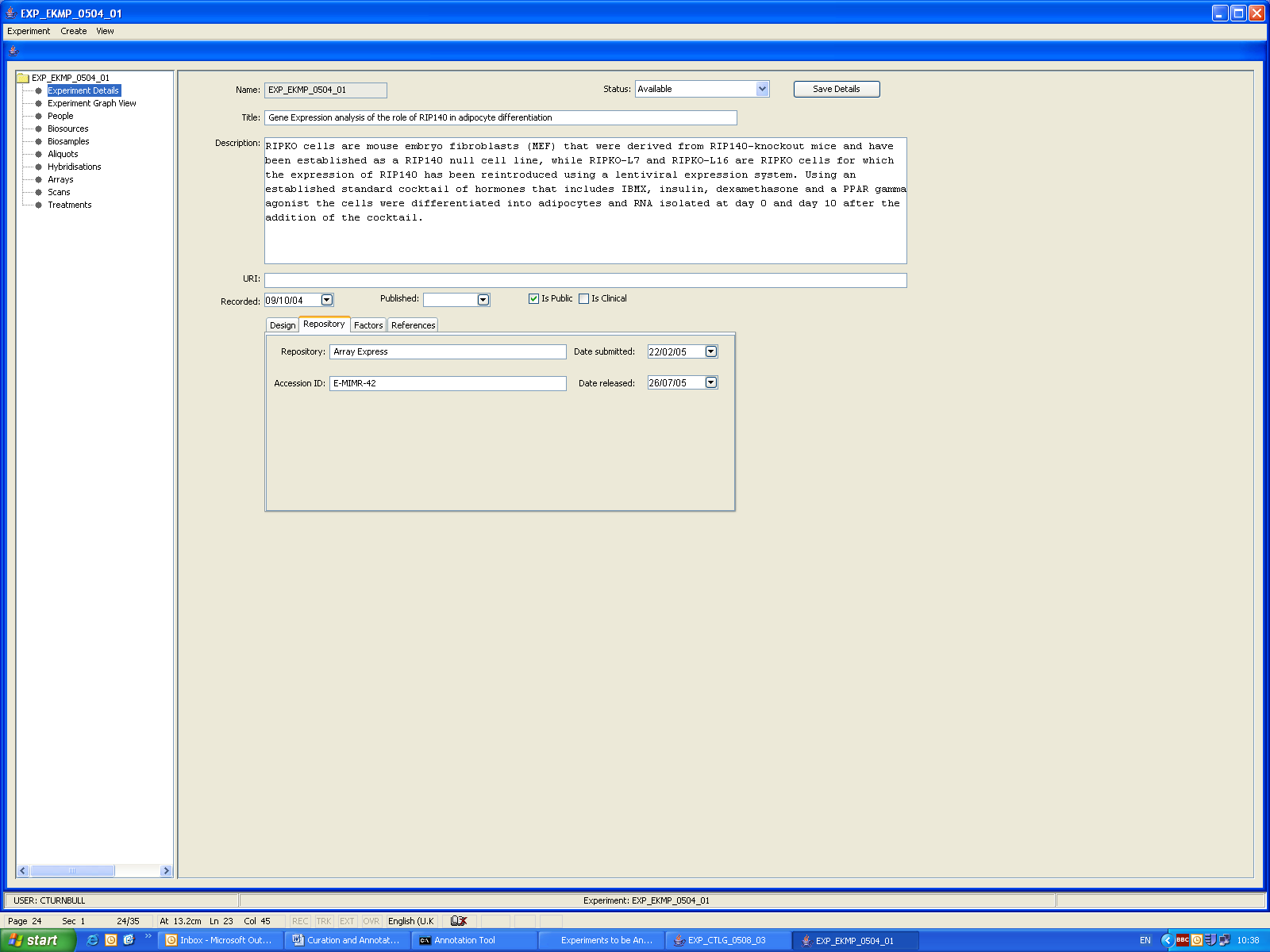


Fig. 4 Screenshot showing experiment with Repository tab populated

In the Experiment Details module of the Annotation Tool there is a ‘Save Details’ button in the top right corner of the right-hand panel; this should be selected before moving on to the next module.

1. **Experiment Graph View**
   - Annotation Tool Views:

In the Annotation Tool the data is displayed in two different formats; graph and table views. In the Experiment Graph View module the details of the experiment are displayed in a graphical form. The other modules display the data in a table view, each showing a stage of the experiment which can be viewed so that the details for all biosamples are available together for comparison.

This module displays a graphical representation of the biomaterials, as generated by the Curation Tool (Fig. 5).


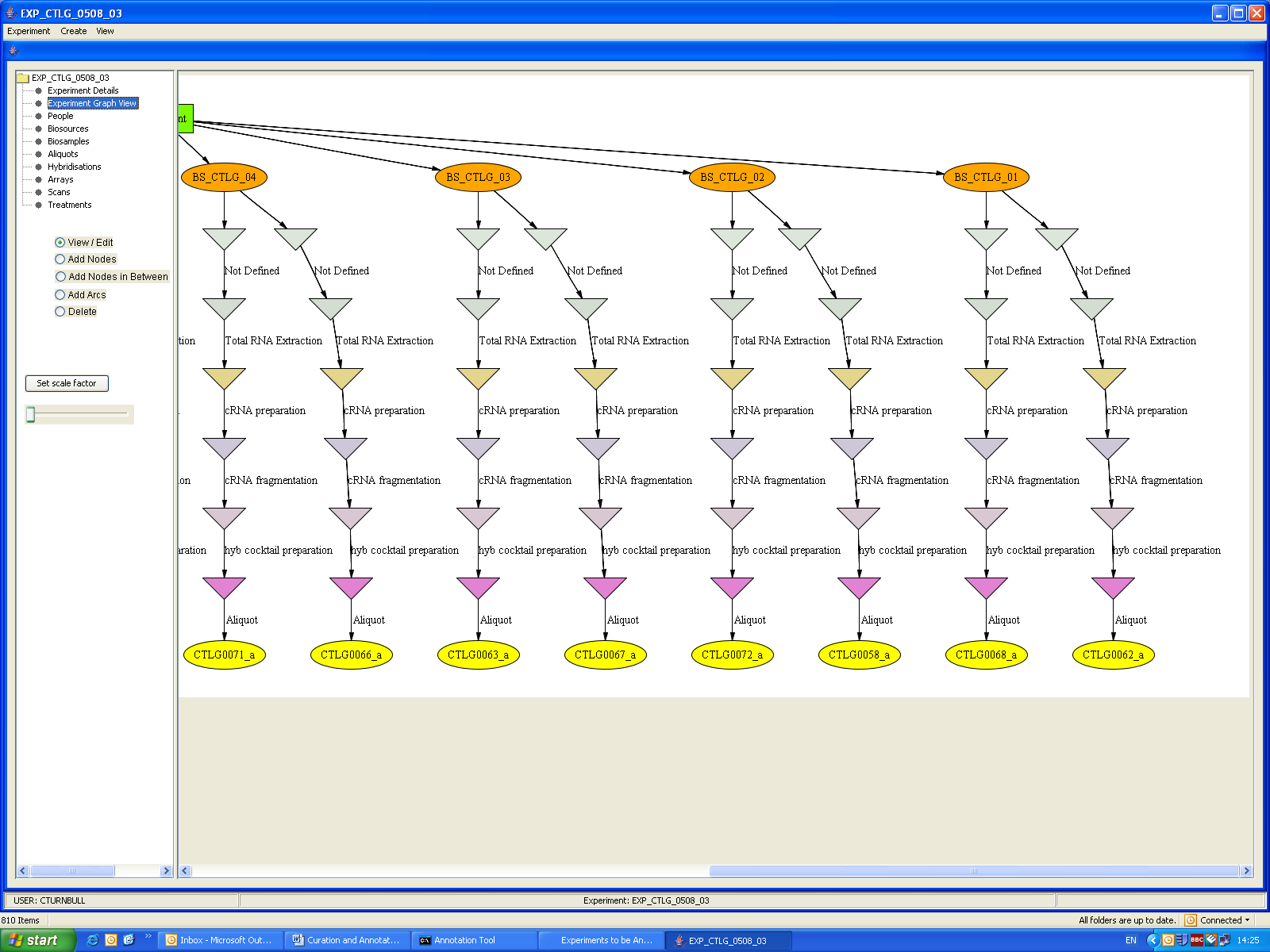


Fig. 5 Screenshot showing the Experiment Graph view.

In this view a node represents a biomaterial and an arc represents a treatment step. These objects are displayed in the same way as in the Curation Tool but are editable.

NOTE: The Curation Tool generates the biosamples in the same order as the user has entered the samples in the Online Annotation Tool.

A pop-up window appears when either a node or an arc is selected (under the View/Edit function). Anything which is changed in these pop-up boxes must be saved before the box is closed (this saves to the local computer memory).

There are a number of different functions available to alter the graph in the middle of the left-hand panel in this module:

- View/Edit: to select biomaterials and treatment steps and either view or edit the information entered in these objects.
- Add Nodes: to add biomaterials, for example in the case of splitting.
- Add Nodes in Between: when an extra treatment stage is added, both a node and an arc are inserted so that none of the information which has already been entered is lost.
- Add Arcs: to add a treatment step. This might be used for splitting or pooling or when biomaterials are manually added to an experiment.
- Delete: when a node or arc needs to be deleted, for example when samples are pooled and the downstream steps for some of the biosamples are not needed.

NOTE: The Annotation Tool sets limits on the graph so that pooling and splitting have to be described separately from other treatment steps. When splitting or pooling occur, a new biosample and treatment step should be added to show where the splitting or pooling has occurred.

New treatment

New Biosample

- Using the Graph view to represent pooling

As pooling can occur at any stage of the experiment and a large number of samples can be pooled, this has to be manually annotated. Using the various functions in the graphical view, the graphs can be amended to best represent how pooling was done.

**Fig. 6a** First, an extra node and arc need to be added to one of the branches which are to be pooled.

Alter the function to ‘Add Nodes in Between’ then select the node above where the new node is to be inserted. The tool will ask whether the existing treatment should be linked to the parent node (the existing one) or the child node (the one which is being created).

You can click ‘Yes’ to link it to the parent and click ‘No’ to link it to the child. In this case click ‘No’. The new biosample and treatment will be blank.

**Fig. 6b** The biosamples downstream of the biosample to be pooled need to be deleted. Select the ‘Delete’ function and then click on each arc and node to be deleted.

**Fig. 6c** To link the node from one branch to the downstream node in another branch select the ‘Add’ arcs function and then click on the node where the arc should start. If the node has been selected correctly then it should be outlined in red. Next select the downstream node in the next branch. The Annotation Tool will recognise that this is a pooling step and will give this arc the treatment name ‘pooling’. This treatment step will not be editable.

**Fig. 6d** The final step is to change the function to ‘View/Edit’ and to add an ontology entry and biosample type to the new node.

- Using the Graph view to represent splitting

As splitting can occur at any stage of the experiment and a large number of samples can be split, this has to be manually annotated. Using the various functions in the graphical view, the graphs can be amended to best represent how splitting was done.

**Fig. 7a** First, an extra node and arc need to be added to the branch which is to be split. Alter the function to ‘Add Nodes in Between’ then select the node above where the new node is to be inserted. The tool will ask whether the existing treatment should be linked to the parent node (the existing one) or the child node (the one which is being created). You can click ‘Yes’ to link it to the parent and click ‘No’ to link it to the child. In this case click ‘No’. The new biosample and treatment will be blank.


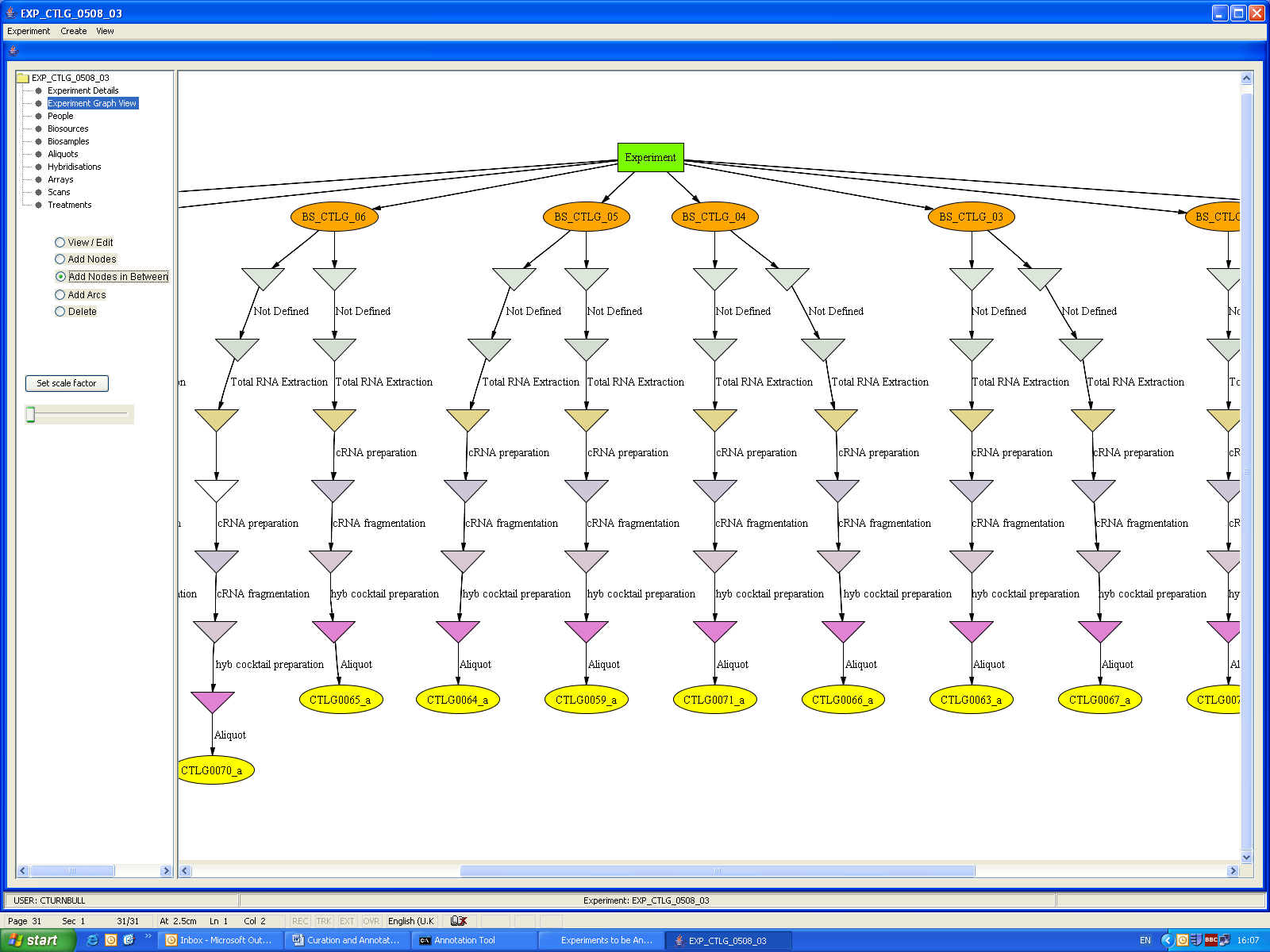

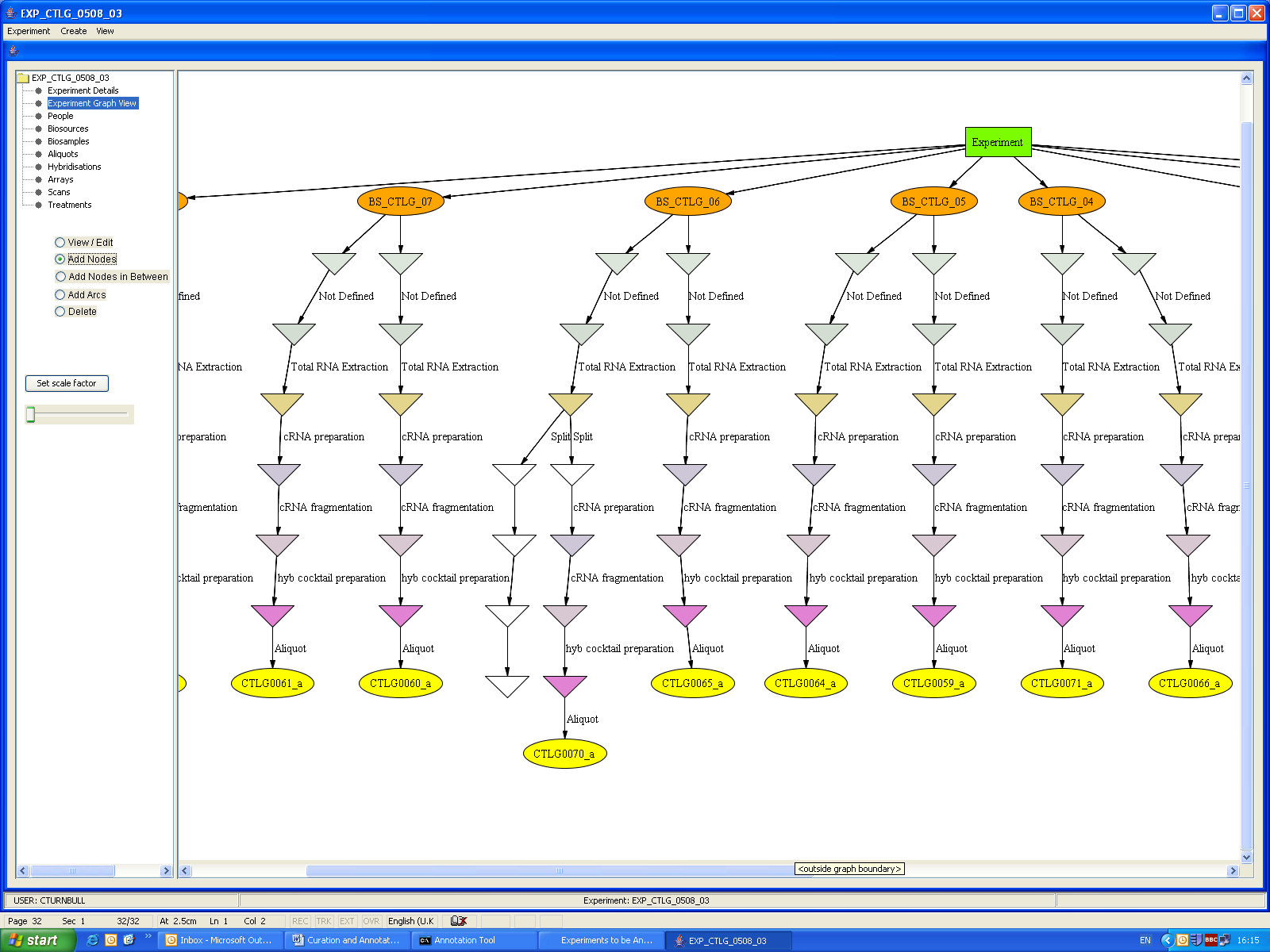


**Fig. 7b** The next step is to add a new node. To do this select the ‘Add Node’ function and select the node above the node you have just created. Change the function to ‘View/Edit’. Open up the matching biosample in the original branch and using this as a guide, fill in the biosample type, ontology terms and any other details. Repeat this until the new branch has the same number of nodes and arcs as the original branch.


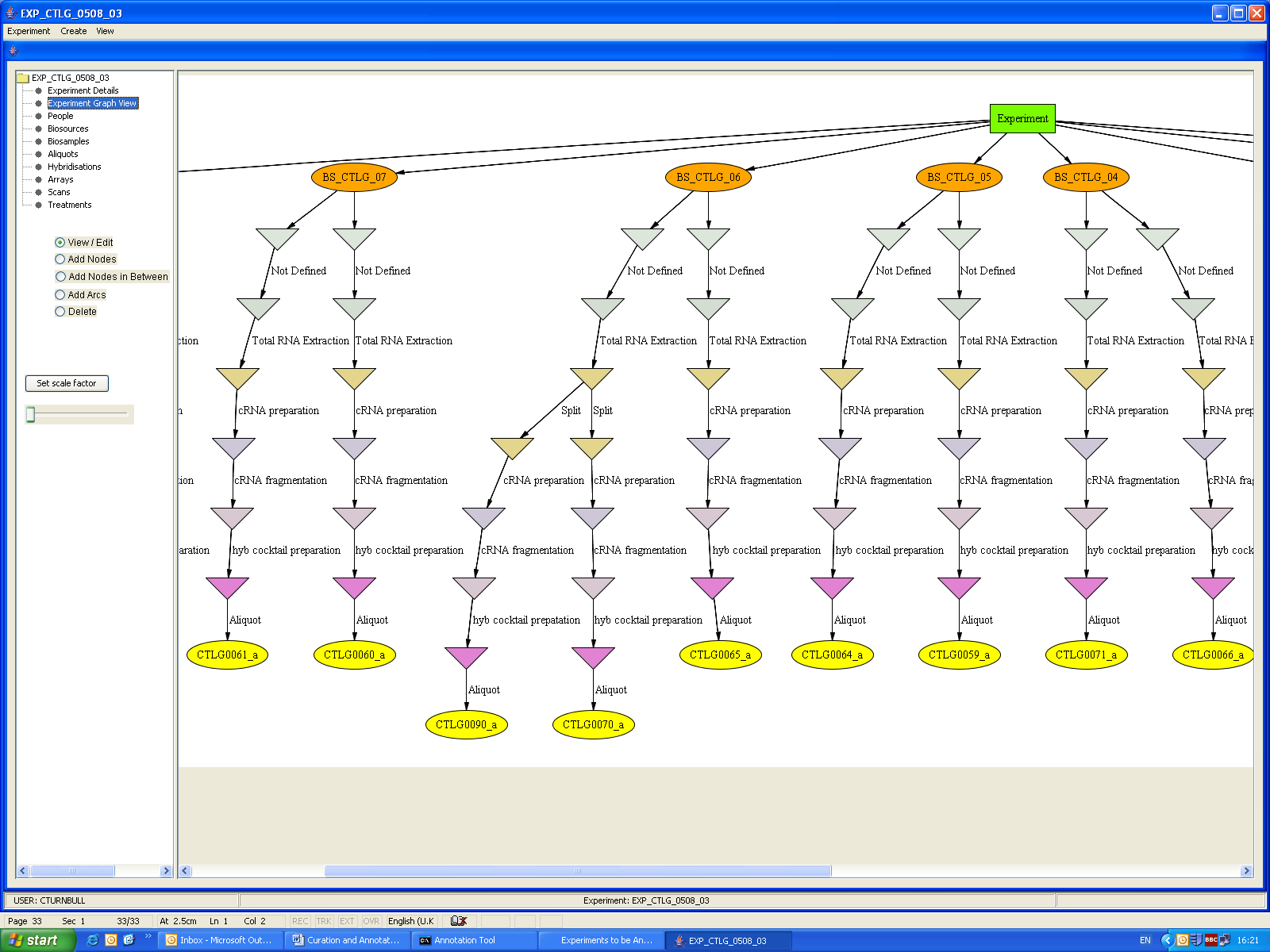


**Fig. 7c** Before creating a new node for the aliquot, make sure that the experiment has been saved to the database and reloaded. It is important that the node before the aliquot in the branch has the biomaterial type Hyb Cocktail; otherwise the Annotation Tool will not recognise that this is an aliquot.

1. **People**

The users assigned to the experiment and their roles can be viewed through the People module. Contact details can be displayed and checked by double-clinking on each user record. A user can be deleted or edited by right-clicking on that record and new users can be added by right-clicking on the header at the top of the right-hand panel.

1. **Biosources/Biosamples**

- Information populated by the Curation Tool:

Based on the labelling method selected by the user, the Curation Tool builds a number of biosamples and treatments. If there is no compound treatment in the experiment then the first step will be an undefined treatment. If there is a compound treatment in the experiment then the Curation Tool will build an undefined treatment step, followed by a compound treatment step. In untreated samples in a compound treatment experiment only an undefined treatment will be built, but samples which are treated with a control substance will still have a compound treatment step.

For the compound treatment steps details of the compound usage needs to be added for each biosample. Select the Compound Application tab in the Compound Treatment step. Add a new entry and select the appropriate composite compound from the list. Here the details of the final concentration of this compound should be added.


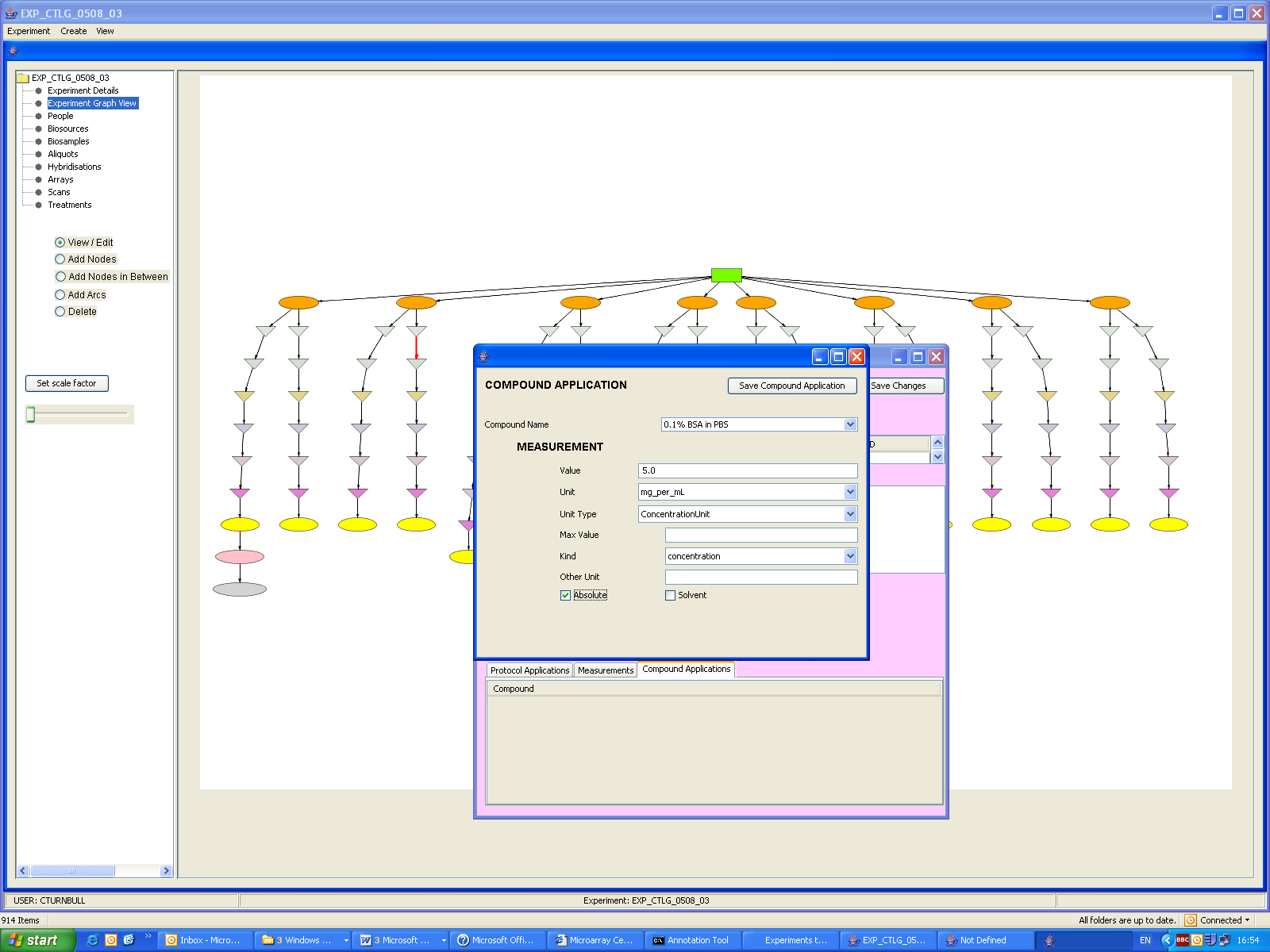


Fig. 8 Example of Compound Application pop-up box

From the total RNA stage onwards the Curation Tool builds a particular series of steps depending on the labelling protocol selected. The Curation Tool will recognise the array type used and tie this in will the labelling protocol to select all of the protocols to be assigned at each step. The Curation Tool automatically assigns the biosample types, the treatment steps and the associated ontology terms, and the protocols linked to these steps.

The first node in the graph represents the biosource. Click on the biosource to bring up a pop-up box. The majority of the fields in this pop-up will already have been populated by the Curation Tool. For each biosource the unique name defined by the user is populated in the Source Name box. The organism type selected by the user is automatically selected from the options in the Organism box.


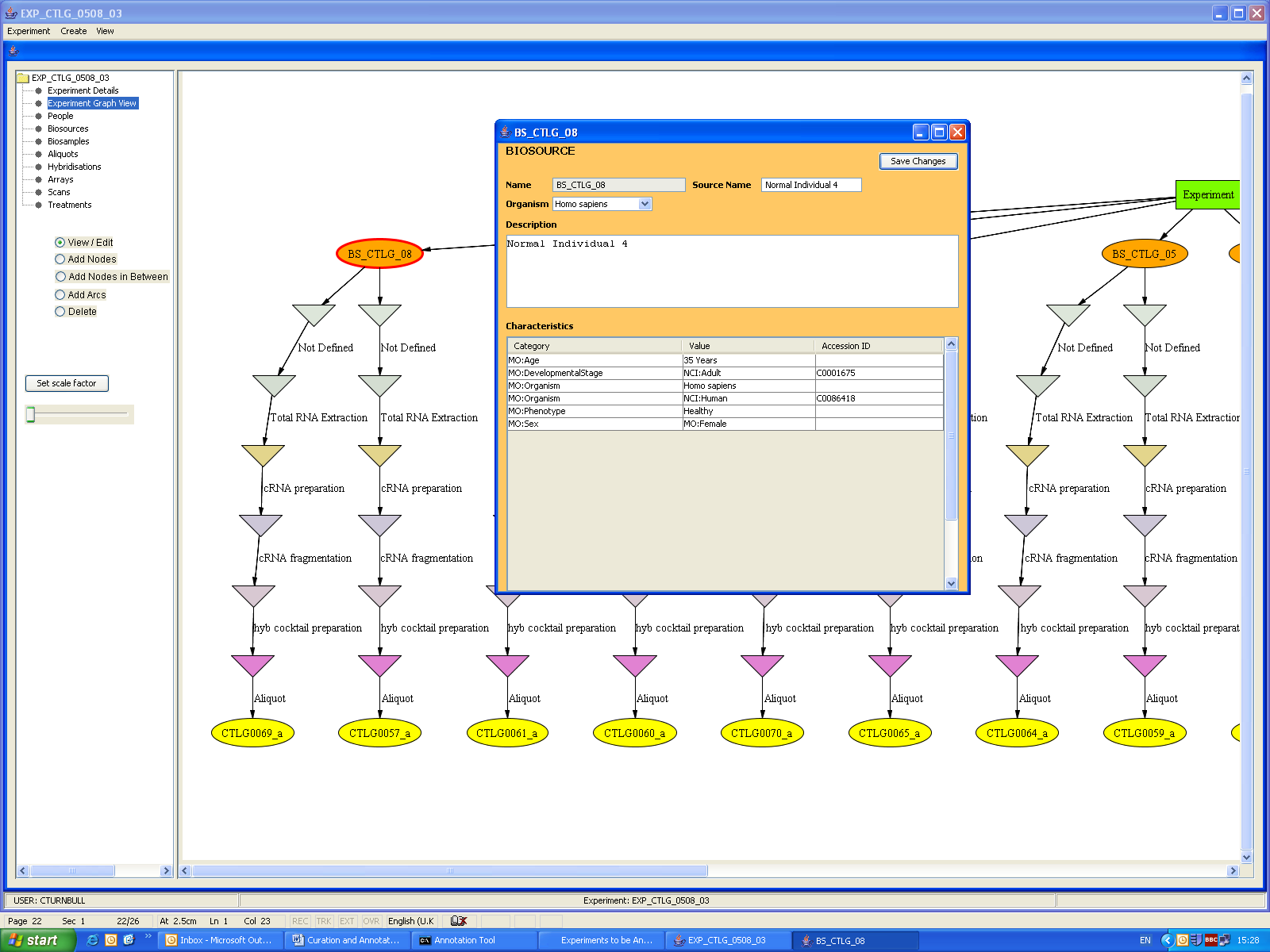


Fig. 9 Screenshot of the Graph view showing the biosource details.

In the Online Annotation Tool the user has a number of options to enter the relevant information for their biosources. The Curation Tool populates the ontology terms according to the boxes the user has entered data into. The terms are populated with either MO: or NCI: terms (with the relevant accession ID) by the Curation Tool. These terms should be checked by the annotator; the most relevant ontology terms kept and the others deleted.


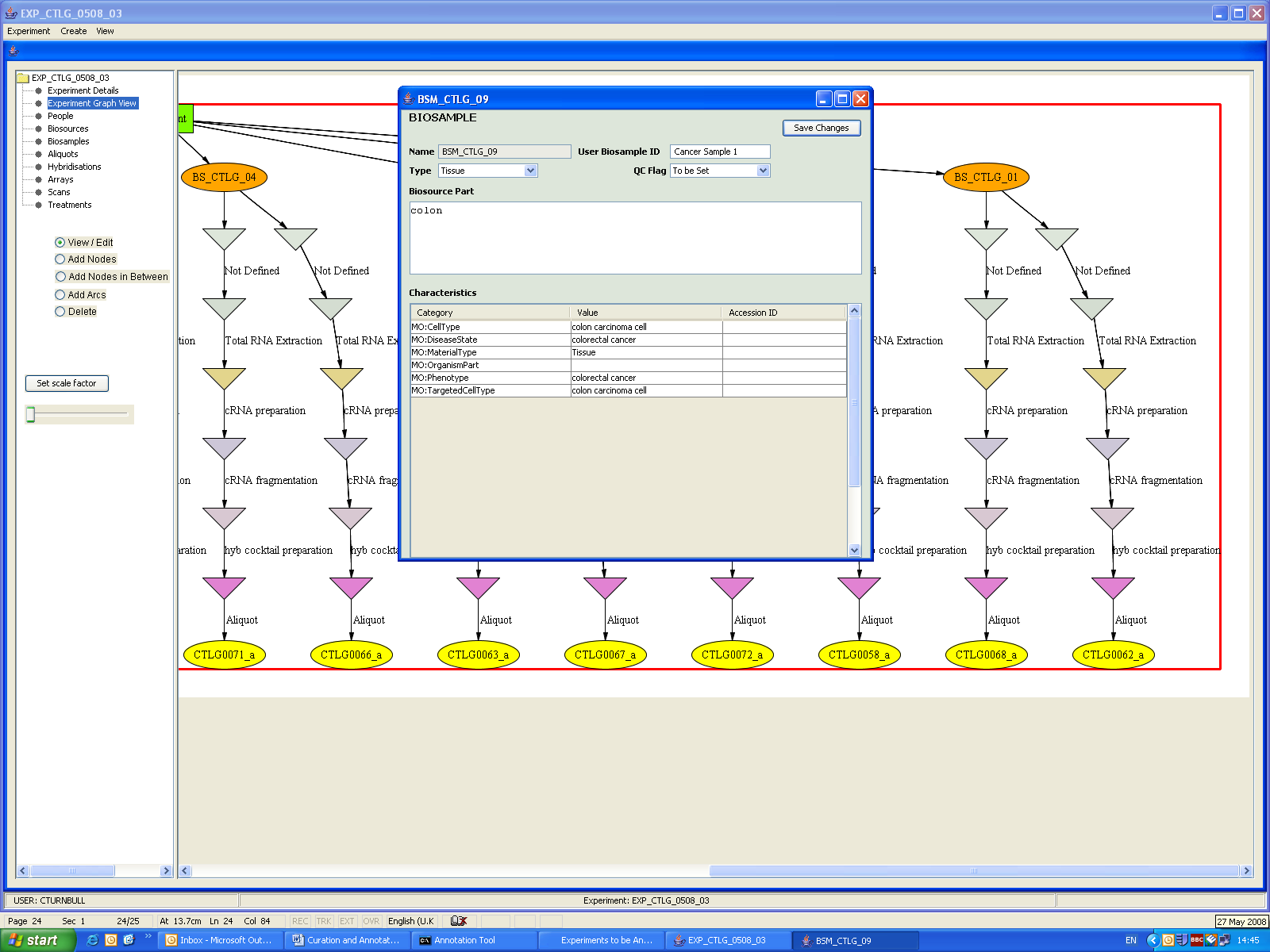


Fig. 10 Screenshot of graph view showing details for the initial biosample.

In the Online Annotation Tool the user has a number of options to enter the relevant information for their biomaterials. The tool populates the ontology terms according to the boxes the user has entered data into. These should be checked; the most relevant ontology terms kept and the others deleted. For example in Figure 9 the user has entered colon carcinoma cell in the targeted cell type/cell type box. The tool recognises this and populates two records one for each of the options. As there was no selection carried out in the experiment then we can say that the cell type is the correct term and the targeted cell type option can be deleted.

As with the biosource node, the type is automatically populated by the Curation Tool. This is based on the sample type the user has selected and may not be appropriate for this stage. This should be checked by the annotator. The user sample ID displayed here corresponds to the unique name entered by the user.

All of the details entered by the user specific to the biosamples are entered into the first biosample in the graph. Some of this information may apply to a biosample further down the graph, in this case remove the record from the ontology entries in the first biosample and enter it into the correct biosample record.

The Curation Tool takes the QC values entered by the user and creates measurement entries in the appropriate treatment step. The correct values for each of the measurements are populated automatically.

For some of the labelling options in the Online Annotation Tool there are two versions; labelling protocol and labelling service. The labelling protocol will be selected when the user has labelled the samples and the labelling service will be selected when the Microarray Centre Team have carried out the labelling. When a labelling protocol option has been selected all of the QC values are automatically entered by the Curation Tool. When a labelling service option has been selected the user will only know the QC values for the total RNA so the QC values for the downstream steps will not be entered. The Curation Tool will create measurement entries at all of the appropriate treatments steps and the values will be entered manually by the annotator.

This process is extendible to all new microarray applications so that experiment graphs can be automatically created for all experiments entered into the tool in the future.

Based on both the QC measurements entered and the bioanalyser traces for each sample (the traces are included with the experiment report in the zip file) a QC flag should be assigned to each sample and entered in the appropriate biomaterial node. See ‘Quality Control of Expression Samples’ file for details of the parameters required for different QC flags.


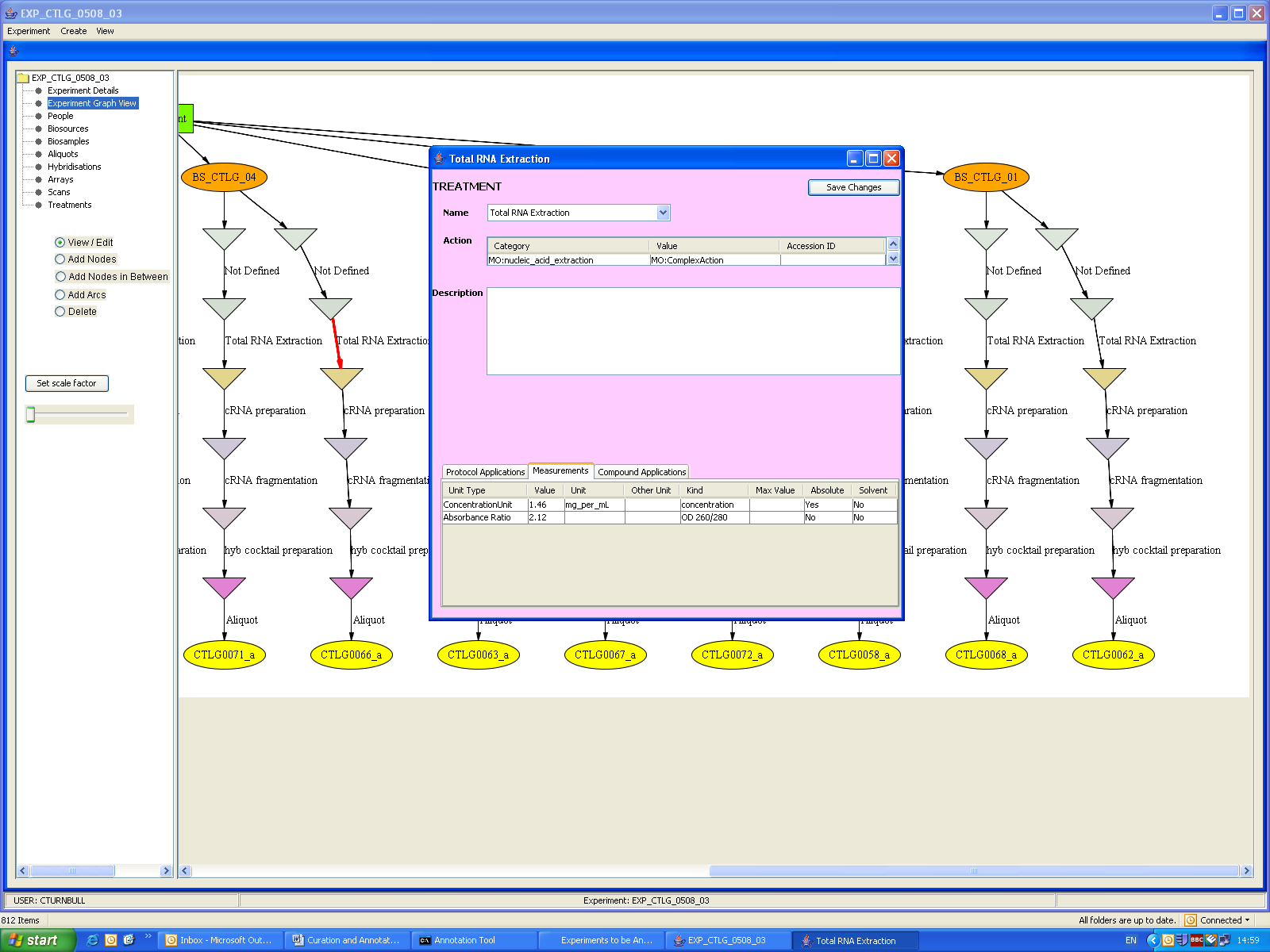


Fig. 11 Screenshot of total RNA treatment step

1. **Protocols**

Bespoke protocols have been created in the Curation Tool; these need to be attached to the appropriate step. Only one protocol can be assigned to each step, so if there is more that one bespoke protocol for a step then a new protocol needs to be created merging the existing bespoke protocols (see Section C for details on creating new protocols). To assign a protocol open up the pop-up box for the correct treatment, right-click on the column header and select ‘Add’. Scroll down the list to select the appropriate protocol.

Any protocol deviations can be added by selecting the appropriate protocol record, right clicking and selecting ‘Edit’.

1. **Aliquots**

All samples are assigned an aliquot number before processing. Like the other biomaterials, the aliquots are assigned a name derived from the four letter user/group head ID. This name is the four initials followed by the sequential sample number.

E.g. If there are 68 CTLG aliquots already present in the database then the Curation Tool will assign CTLG0069_a to the next aliquot to be created. The biosamples names are created in the order they were entered into the Online Annotation Tool, so this numbers usually do not correspond with the numbers at the end of the aliquot ID. The _a indicates that this is the first aliquot created by the Microarray Centre. If the sample failed and was re-fragmented and re-hybridised then this would be classed as a new aliquot and would be called CTLG0069_b.

When manually creating an aliquot the Annotation Tool will recognise that a node is being linked to a Hyb Cocktail and the four letter user/group head ID will automatically be populated, add the correct number to the end of the aliquot ID before saving.

1. **Hybs, Arrays and Scans**

The Curation Tool does not build any information for the Hybridisations, Arrays and Scans. This must be manually entered by the annotator as the naming of these depends upon who has carried out the hybridisation and what the date is.


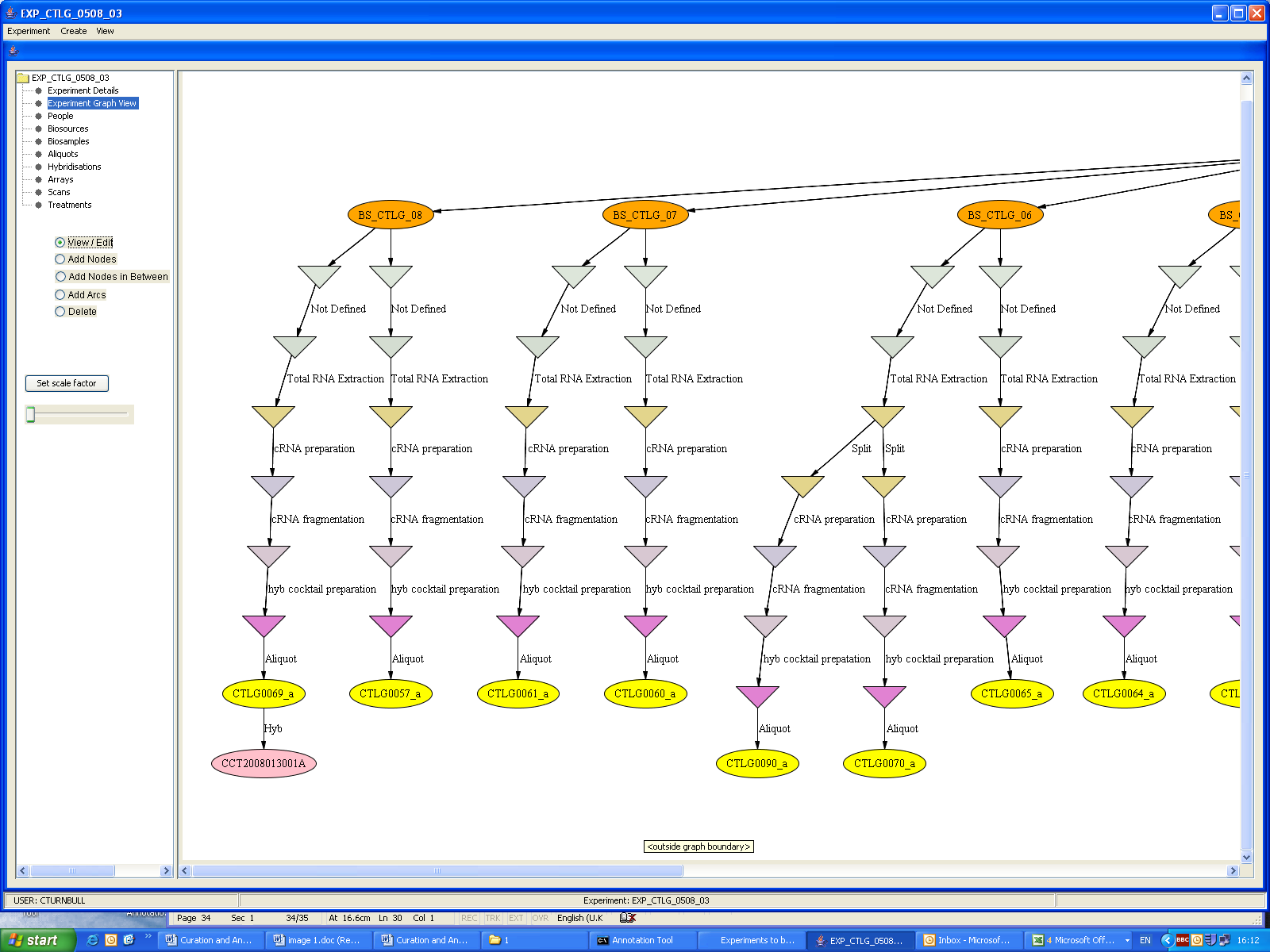


Fig. 12 Screenshot showing branch of graph with hybridisation step arc and array node added.

To add a hybridisation, select ‘Add Node’ and click on the appropriate aliquot. The Annotation Tool will recognise that a hybridisation is being created and will enter today’s date in the correct format for the hybridisation name. In Fig. 12 the following hybridisation ID has been assigned; CCT2008013001A. The first three letters correspond to the initials of the person carrying out the hybridisation. The subsequent numbers correspond to the date, the 30^th^ of January 2008, in YYYYMMDD format.

The last two numbers represent the order the samples are processed on the day of hybridisation and this should be entered so that these final two digits correspond to the order of the aliquot IDs. E.g. if CTLG0069_a is the first sample processed on that day and aliquot CTLG0070_a is the second then hybridisation CCT2008013001A will correspond to the to CTLG0069_a and CCT2008013002A will correspond to CTLG0070_a. The last letter is either A, B or C depending on the type of whole genome array used, or T for a Test3 array.

When the hybridisation has been saved the pop-up box can be opened by clicking on the Hyb Treatment and all of the details of the hybridisation should be entered. The factors assigned to the samples should be linked at this stage of the annotation by selecting the correct option from the drop-down list of factors. These factor names correspond to those assigned by the user and these names may also have been curated. The details specific to the array should also be entered at this point.

(B)

(A)


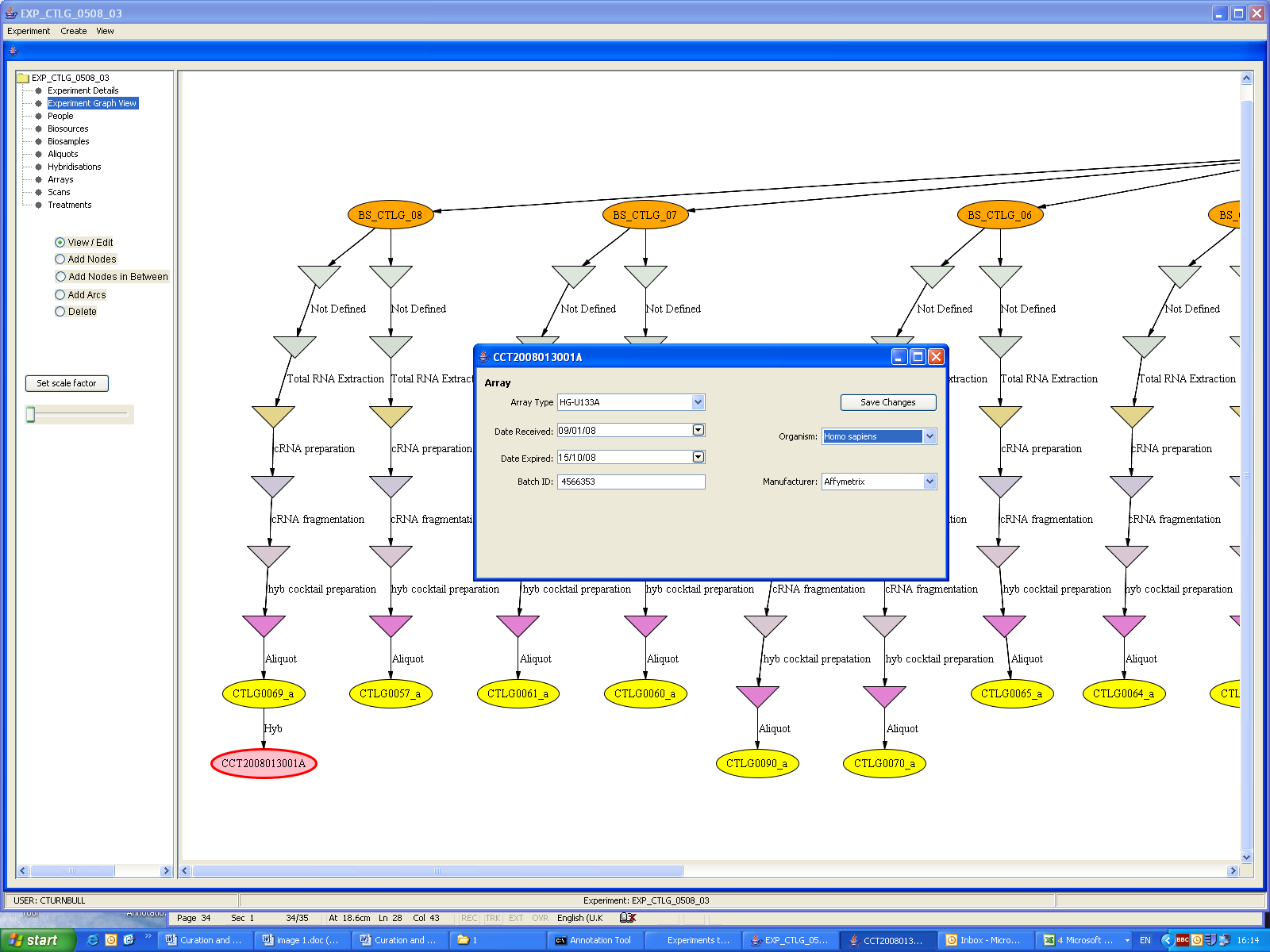


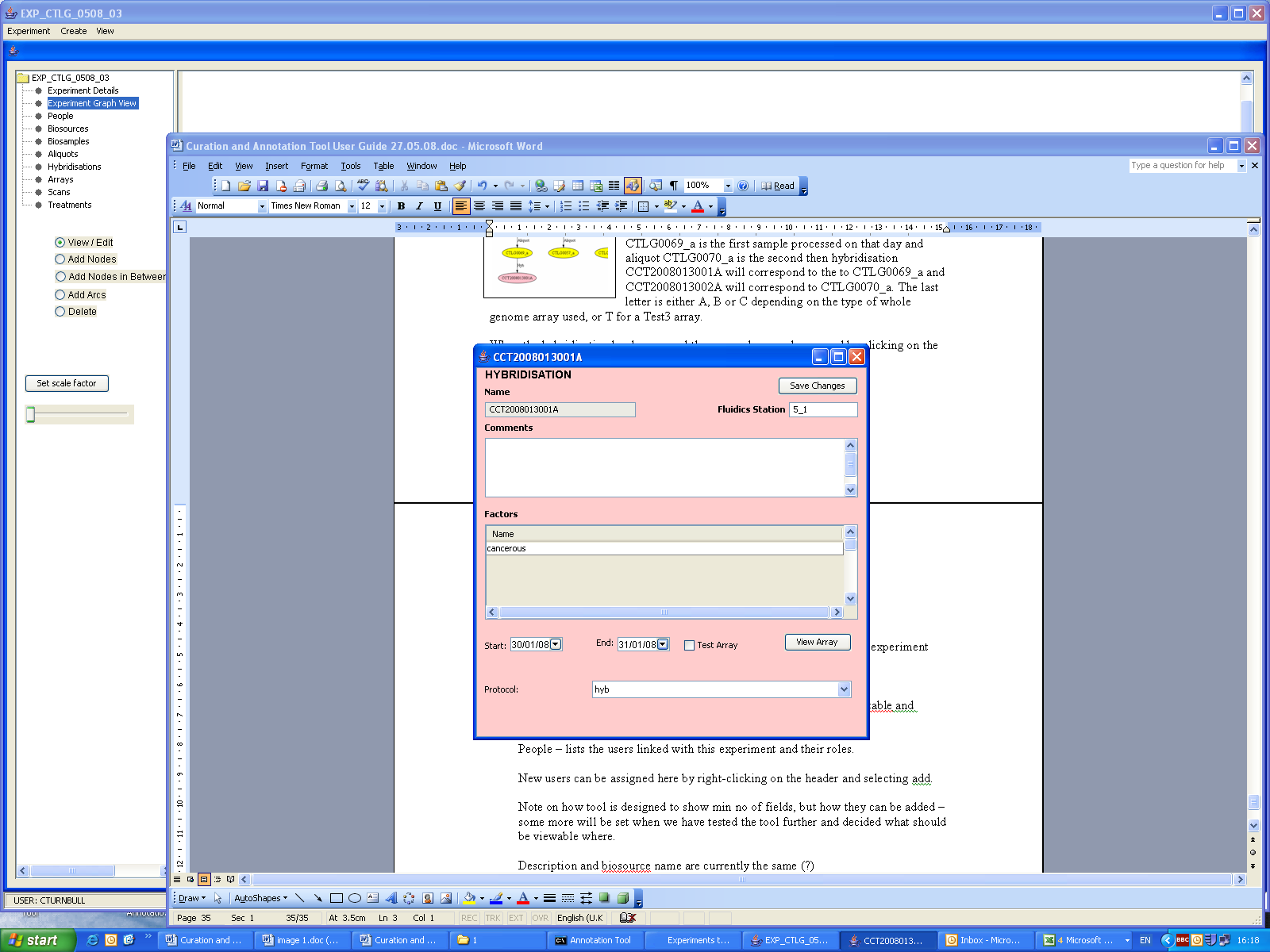


Fig. 13 (A) Example of Hybridisation Step pop-up box, (B) Example of Array pop-up box.

The final stage of the annotation is to create the scan nodes. These are the same as the hybridisation name, except that they have an aa suffix to indicate that they are scans. The aa indicates that these arrays have been scanned after they have been washed with an antibody for biotin and stained with SAPE again.


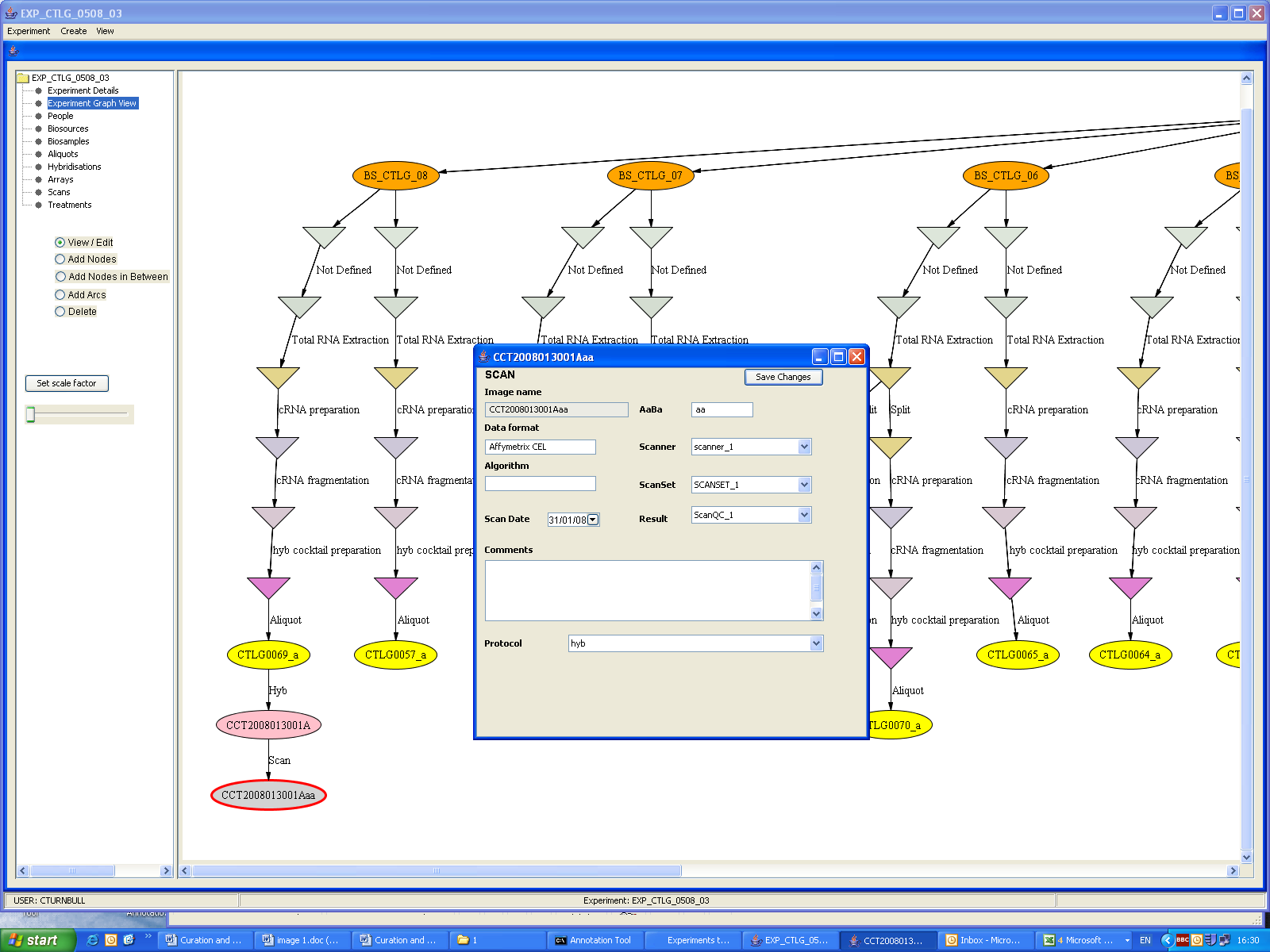


Fig. 14 Example of Scan pop-up box

In this module the details of the scanner settings and the quality of the scan are recorded. A QC flag should be assigned based on how well the array data performs for a variety of quality control parameters looking at RawQ, Background, %P and the signal and 3’ to 5’ values for a number of housekeeping and control genes. See ‘Quality Control of Expression Samples’ file for details of the parameters required for different QC flags.

When all of the required nodes and arcs have been added to the graphical view the experiment should be saved and reloaded.

The annotation of the nodes and treatments related to the hybridisation process is currently manual, but this will become automated on introduction of the third tool; the Laboratory Data Mapping Tool, which will interact with both the Annotation Tool and Affymetrix Command Console.

- Table View Modules:

All of the modules downstream of the People module are modules which display the experimental information as tables. These tables divide the details up so that a section of the information can be viewed at once. An example of this is that the biosources and all of the details entered into these records can be viewed in the Biosources module.

For the Arrays module all of the details provided for each array can be viewed in one place. This means that the data can be accessed without opening up a different pop-up box for each array.

The Annotation Tool is currently designed to display the minimum number of fields in these table views, but extra fields can be added by right-clicking on the header and selecting add fields. The data can be sorted alphabetically by a particular column by clicking on the header of the column.

If the details are found to be incorrect in one of the tables, double click on one of the records to open and edit it (these records are the same as those accessed through the graph view).

- Annotation Checking Procedure

Once annotation has been completed, the experiment will be independently checked by another member of the Microarray Centre Team for consistency and accuracy. Once this has been done, change the status to ‘available’ and inform the data warehousing team that annotation is complete so that the experiment can be made available to the user via MiMiR online.
