# Supplementary figures and images for: MiMiR – an integrated platform for microarray data sharing, mining and analysis
Source: BMC Bioinformatics. 2008 Sep 18;9:379. doi: 10.1186/1471-2105-9-379 (PMC2572073; doi:10.1186/1471-2105-9-379)

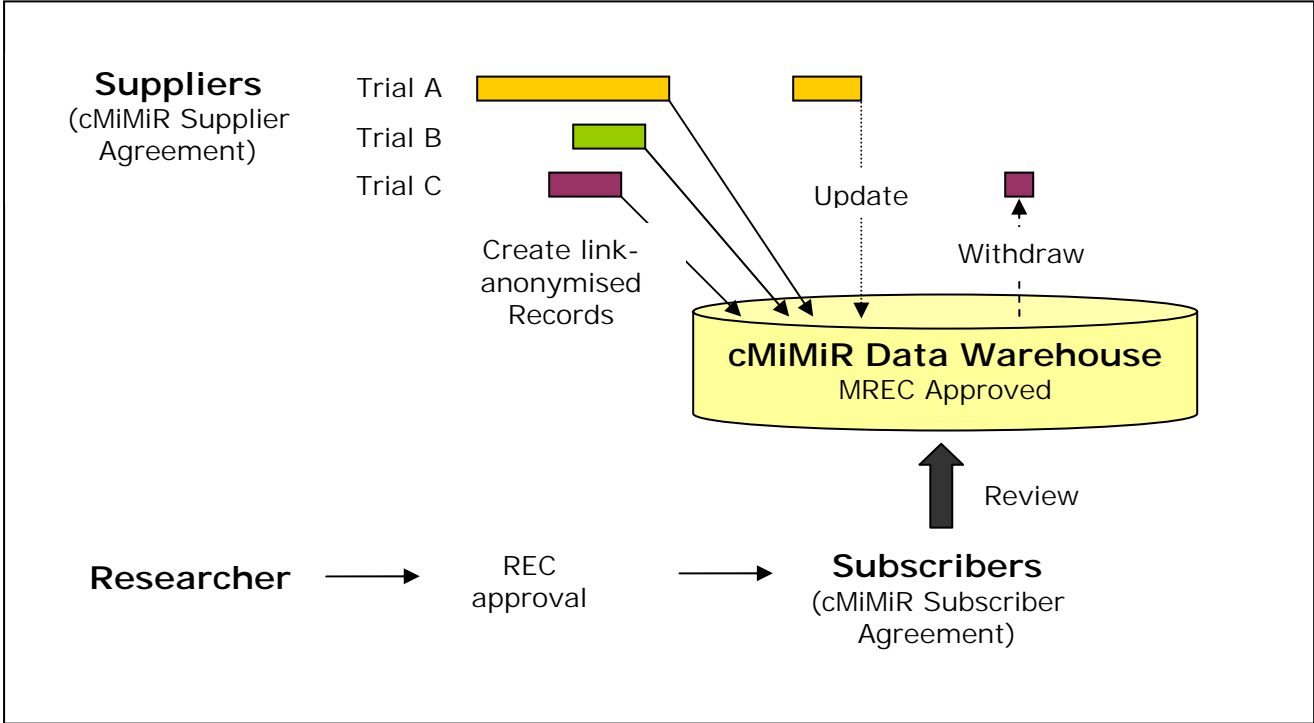

Supplement: Additional File 4 — Ethical framework for the supply of clinical data to the clinical part of MiMiR (cMiMiR) and subsequent data retrieval from researchers. Suppliers of microarray and clinical data, e.g. clinicians/researchers running a clinical trial with independent ethical approval, create and assign unique anonymisation keys (Level 4 pseudo-anonymised data) for each participating patient. All identifying information from the respective clinical record (e.g. name, address, date of birth) is removed before sending these records for storage into cMiMiR. A 'cMiMiR Supplier Agreement' between Suppliers and Custodians of cMiMiR covers the requirements and obligations of both parties regarding the import of clinical records into cMiMiR (Additional File 6). Researchers, designated "Subscribers", wishing to access data in cMiMiR for a particular project need to apply for Research Ethics Committee (REC) approval before they can be granted rights to access data (and be bound by the 'cMiMiR Subscriber Agreement', Additional File 7). Suppliers can collect and store additional information in cMiMiR for existing patients (e.g. treatment follow-up data) by updating the relevant cMiMiR record using the original anonymisation key. Trained annotators implement the updates to ensure consistency in annotation and quality and integrity of the data. Patients and volunteers involved in clinical projects have the right to withdraw their data from cMiMiR at any stage and without any justification. This is done by the Supplier who conducted the clinical trial and who holds the anonymisation key to the individual record. It is important to ensure that patients and volunteers participating in clinical trials are fully aware of the use of their anonymised clinical records for analysis by authorised researchers during the consent process. Clinical and genetic data derived from biological materials stored in licensed tissue banks are covered under standard consenting process. Prospective clinical trials explicit [file 1471-2105-9-379-S4.pdf]
