# Supplementary material for: MiMiR – an integrated platform for microarray data sharing, mining and analysis
Source: BMC Bioinformatics. 2008 Sep 18;9:379. doi: 10.1186/1471-2105-9-379 (PMC2572073; doi:10.1186/1471-2105-9-379)
Supplement: Additional File 5 — Example of clinical information supplied by a clinical researcher (first three columns) and how it is codified using the Data Mapping Tool prior to import into MiMiR. Each concept must be explicitly mapped to unambiguous, uniquely identified terms. The Unified Medical Language System (UMLS version2007AB) which provides a Metathesaurus of clinical terms is used to provide the identifiers that are persisted within MiMiR. [file 1471-2105-9-379-S5.doc]

Supplementary Table 1

| Clinical data from Supplier database/Patient Management System | | | Codified data using the Data Mapping Tool for input into cMiMiR |
| --- | --- | --- | --- |
| **Field Type** | **Field Name** | **Value** | **Encoded Values** |
| Numeric | Age | 47 years | **Field Details:** Age [C0001779]  **Value:** 47  **Units:** Precision – year [C1561544]  **Type:** Time [C0040223] |
| Class | Sex | M | **Field Details:** Gender [C0079399]  **Value:** Male Gender [C0024554] |
| Class | Disease Progression | Indolent | **Field Details:** Disease progression [C0242656]  **Value:** Indolent [CL054831] |
| Class | Disease Type | CML | **Field Details:** Disease [C0012634]  **Value:** Myeloid Leukemia, Chronic [C0023473] |
