# Supplementary material for: MiMiR – an integrated platform for microarray data sharing, mining and analysis
Source: BMC Bioinformatics. 2008 Sep 18;9:379. doi: 10.1186/1471-2105-9-379 (PMC2572073; doi:10.1186/1471-2105-9-379)
Supplement: Additional File 6 — cMiMiR Supplier Agreement. [file 1471-2105-9-379-S6.doc]

| cMiMiR Supplier Agreement | |
| --- | --- |
| **Title of Project** | The Development of a Data Warehouse for the Collection of Clinical Microarray Gene Expression Information |
| **Principal Investigators** | Timothy Aitman, Professor of Clinical and Molecular Genetics.  Dr. Laurence Game, Head of Microarray Centre. |
| **Contact Details** | Microarray Centre  Medical Research Council Clinical Sciences Centre  Hammersmith Hospital  Du Cane Road  London W12 0NN  Tel: 020 8383 8336  Fax: 020 8383 8557  Website: <http://www.csc.mrc.ac.uk/> |

This agreement is between Clinical Trials Investigators (the **Suppliers**) and the **Custodians** of the cMiMiR database. It covers the export of clinical trial data into the cMiMiR database. Once data has been deposited into the cMiMiR database it can be made available to other scientists (the **Subscribers**) through a formal registration process.

| General Statement | **Neither** Custodians **nor the** Suppliers **make any representation or warranties whatsoever in respect of the data.**  **Although the cMiMiR database has been prepared using reasonable standards, and while there are no indications or reasons to believe that there exist inaccuracies or defects in the cMiMiR database,** Custodians **of the cMiMiR database make no representation or warranties of any kind, including without limitation warranties of merchantability or fitness for a particular purpose.**  Custodians **and** Suppliers **shall not warrant the accuracy of information contained within any cMiMiR database and all responsibility pertaining to the use thereof under this licence is hereby assumed by the** Subscribers **accessing the cMiMiR database.**  Custodians **shall not be liable for loss of the data being accessed.**  Custodians **and** Suppliers **shall not warrant the accuracy and timeliness of the cMiMiR database.**  Custodians **of the cMiMiR database shall not be liable to** Suppliers **or any third party for any incidental, special or consequential loss or damage stemming from omitted or inaccurate data. The** Subscribers **assume the sole responsibility for all use of and agree to indemnify and hold** Custodians **of the cMiMiR database and its** Suppliers **harmless from any liability or claim of any person arising from the** Subscriber’s **use of the cMiMiR database.**  **The** Suppliers **acknowledge that** Custodians **of the cMiMiR database do not guarantee or warrant the correctness, completeness, currency, merchantability or fitness of purpose of the information. The** Suppliers **also acknowledge that every decision, of some degree or another, represents an assumption of risk. The** Suppliers**, therefore, agree that** Custodians **of the cMiMiR database shall not be liable to the** Suppliers **for any loss or injury arising out of, or caused, in whole or in part by** Custodians **of the cMiMiR database’s negligent acts or omissions in procuring, compiling, collecting, interpreting, reporting, communicating or delivering information. The** Suppliers **agree that** Custodians **of the cMiMiR database will not be liable for any consequential damages.**  Custodians **and** Suppliers **accept no responsibility whatsoever expressed or implied, with respect to the information contained in the cMiMiR database.**  Private data **is defined as data which has been deposited within the Data Warehouse by a** Supplier **which is not available for** Subscriber **access, unless by direct agreement with the** Supplier**.**  Public data **is defined as data which has been deposited within the Data Warehouse by a** Supplier **which is available to be viewed by and transferred to a** Subscriber **of the Data Warehouse, without the need for a direct agreement between the** Supplier **and the** Subscriber**.**  **The** Suppliers **understand that data which has been provided to the Data Warehouse remains** private **until such time as the** Suppliers **instruct the** Custodians **to make this data** public**.**  **A** Scientific Advisory Committee **is defined as a local committee of scientist who are appointed to act for the** Custodians **to assess the scientific merit of any application by individuals to become** Subscribers **to the Data Warehouse.**  **Any potential** Subscriber **access will be controlled by the** Custodians**, dependent on approval by both the** Scientific Advisory Committee **and a** Research Ethics Committee**.** |
| --- | --- |
| *Anonymisation* | ***The information proved to*** *Custodians* ***of the cMiMiR database has been anonymised before export to the cMiMiR database. No names, addresses or any identifying data of the patient or family member of the patient have been included within the exported data to the cMiMiR database.***  ***The key which allows for the identification of individual patients is stored securely, independent of the*** *Custodians* ***of the cMiMiR database. This key will be retained securely for a period of at least 15 years after the conclusion of the original clinical trial. During this time the*** *Suppliers* ***undertake to maintain contact with the*** *Custodians****.***  ***The*** *Suppliers* ***undertake not to provide any information to the*** *Custodians* ***of cMiMiR as to the identity of the individual patient whose data, in whole or in part, is exported to the cMiMiR database.*** |
| *Consent* | ***Data which is provided to the cMiMiR database from the*** *Suppliers* ***is from patients who have either:***  ***(a) given informed consent for the cMiMiR project;***  ***(b) given informed consent to a COREC approved Research Tissue Bank service (allowing for third party use of link-anonymised, genetic and clinical data);***  ***(c) been unable to provide informed consent. In this case, this must be considered by the*** *Suppliers’* ***original approving*** *Research* *Ethics Committee* ***before being ratified by*** *the Eastern Multicentre Research**Ethics Committee* ***on a case by case basis.***  ***Only after one of these three routes has been followed the*** *Custodians* ***of cMiMiR are able to accept this data for storage.*** |
| *Ethical Approval* | ***The*** *Suppliers* ***have*** *Research* *Ethics Committee* ***approval, or any other body deemed by law, regulation or custom, to allow the submission of clinical and gene expression data into the cMiMiR database and a copy of the approval is available on request.*** |
| *Security* | ***The*** *Suppliers* ***acknowledges that the original patient data which has been supplied to the data warehouse is held securely by the*** *Suppliers* ***and that all reasonable steps have been taken to safeguard this data and maintain confidentiality. The*** *Suppliers* ***carry indemnity in the effect of any breach of confidentiality.*** |
| *Data Protection Act, 1998* | ***A*** *Data Controller* ***is defined as the person (individual or organisation) who decides the purposes for which the information will be held, organised, adapted, altered and disclosed.***  *Data Controllers* ***supplying the warehouse with data have specific statutory obligations to ensure that the data is processed (i.e. held, adapted, altered and/or disclosed) in accordance with the data subject's rights and that they have arrangements in place to ensure that there is no unauthorised or unlawful processing or accidental loss, destruction or damage during transfer from the original holder to the data warehouse.  In practice, this means that the*** *Data Controllers* ***will enter into an appropriate*** *Data Processing Agreement* ***with the****Custodians* ***of the warehouse.***  ***The*** *Suppliers* ***acknowledge that they are acting as*** *Data Controllers* ***as they are in possession of the identifiers to link the data to the individuals to whom the information relates.*** |
