# Supplementary material for: MiMiR – an integrated platform for microarray data sharing, mining and analysis
Source: BMC Bioinformatics. 2008 Sep 18;9:379. doi: 10.1186/1471-2105-9-379 (PMC2572073; doi:10.1186/1471-2105-9-379)
Supplement: Additional File 7 — cMiMiR Subscriber Agreement. [file 1471-2105-9-379-S7.doc]

| cMiMiR Subscriber Agreement | |
| --- | --- |
| **Title of Project** | The Development of a Data Warehouse for the Collection of Clinical Microarray Gene Expression Information |
| **Principal Investigators** | Timothy Aitman, Professor of Clinical and Molecular Genetics.  Dr. Laurence Game, Head of Microarray Centre. |
| **Contact Details** | Microarray Centre, Medical Research Council  Clinical Science Centre  Hammersmith Hospital  Du Cane Road  London W12 0NN  Tel: 020 8383 8336  Fax: 020 8383 8557  Website: <http://www.csc.mrc.ac.uk/> |

This agreement is between clinical researchers wishing to examine the contents of the cMiMiR database (the **Subscribers**) and the **Custodians** of the cMiMiR clinical microarray data warehouse. It covers the review of clinical trial data held within the cMiMiR database. This data has been deposited within the cMiMiR database by Clinical Trials Investigators (the **Suppliers**) under the supervision of the **Custodians** of cMiMiR.

In order for a **Subscriber** to be granted access to the cMiMiR database it is necessary for a formal request to be provided to the **Custodians** to request this access, with a description of the intention of use. Access to the cMiMiR database will only be granted once:

1. the scientific merit has been assessed by the cMiMiR **Scientific Advisory Committee** and deemed to be of sufficient scientific merit; and
2. the ethical aspects of the proposed research have been agreed to by a registered **Research Ethics Committee**; and
3. the overseeing **Multi-Centre Ethics Committee** has ratified the researcher’s application to become a cMiMiR **Subscriber**.

Only after all three of the above-mentioned steps have been successfully executed will it be possible for an applicant to be granted access to the cMiMiR database.

**Private data** is defined as data which has been deposited within the Data Warehouse by a **Supplier** which is not available for **Subscriber** access, unless by direct agreement with the **Supplier**.

**Public data** is defined as data which has been deposited within the Data Warehouse by a **Supplier** which is available to be viewed by and transferred to a **Subscriber** of the Data Warehouse, without the need for a direct agreement between the **Supplier** and the **Subscriber**.

The **Subscribers** understand that data which has been provided to the Data Warehouse remains **Private** until such time as the **Suppliers** have instructed the **Custodians** to make this data **Public**.

| General Statement | ***Neither*** *Custodians* ***of the cMiMiR database nor any data*** *Supplier* ***makes any representation or warranties whatsoever in respect of the data.*** |
| --- | --- |
| *Quality of database* | **Although the cMiMiR database has been prepared using reasonable standards, and while there are no indications or reasons to believe that there exist inaccuracies or defects in the cMiMiR database,** Custodians **of the cMiMiR database and its** Suppliers **make no representation or warranties of any kind, including without limitation warranties of merchantability or fitness for a particular purpose.** |
| *Data Quality* | **Neither** Custodians **of the cMiMiR database nor its** Suppliers **warrant the accuracy of information contained within any cMiMiR database and all responsibility pertaining to the use thereof under this licence is hereby assumed by the party accessing (the** Subscriber**) the cMiMiR database.** |
| *Data loss* | **Neither the** Custodians **of the cMiMiR database or its** Suppliers **shall be liable for loss of the data being accessed.** |
| *Timeliness* | **Neither the** Custodians **of the cMiMiR database or its** Suppliers **warrant the accuracy and timeliness of the cMiMiR database.** |
| *Loss or damage resulting from information* | Custodians **of the cMiMiR database shall not be liable to** Subscriber **or any third party for any incidental, special or consequential loss or damage stemming from omitted or inaccurate data. The** Subscriber **assumes the sole responsibility for all use of and agrees to indemnify and hold** Custodians **of the cMiMiR database and its suppliers harmless form any liability or claim of any person arising from the subscriber’s use of the cMiMiR database.** |
| *Risk associated with use of information* | **The** Subscriber **acknowledges that** Custodians **of the cMiMiR database do not guarantee or warrant the correctness, completeness, currency, merchantability or fitness of purpose of the information. The** Subscriber **also acknowledges that every decision, to some degree or another, represent an assumption of risk. The** Subscriber**, therefore, agrees that** Custodians **of the cMiMiR database shall not be liable to the** Subscriber **for any loss or injury arising about of, or caused, in whole or in part by** Custodians **of the cMiMiR database’s negligent acts or omissions in procuring, compiling, collecting, interpreting, reporting, communicating or delivering information. The** Subscriber **agrees that** Custodians **of the cMiMiR database will not be liable for any consequential damages.** |
| *Responsibility for information* | Custodians **of the cMiMiR database accepts no responsibility whatsoever expressed or implied, with respect to the information contained in the cMiMiR database.**  **The** Subscriber **accepts responsibility not to pass on or sell data derived from the cMiMiR database unless already agreed with the cMiMiR** Scientific Advisory Committee **and the responsible** Research Ethics Committee**.** |
