# Supplementary material for: MiMiR – an integrated platform for microarray data sharing, mining and analysis
Source: BMC Bioinformatics. 2008 Sep 18;9:379. doi: 10.1186/1471-2105-9-379 (PMC2572073; doi:10.1186/1471-2105-9-379)
Supplement: Additional File 8 — cMiMiR Consent Form. [file 1471-2105-9-379-S8.doc]

| **Patient Information Sheet (Prospective Recruitment)** | |
| --- | --- |
| Title of Project | The Development of a Data Warehouse for the Collection of Clinical Microarray Gene Expression Information |
| Principal Investigators | Timothy Aitman, Professor of Clinical and Molecular Genetics.  Dr. Laurence Game, Head of Microarray Centre. |
| Contact Details | Microarray Centre,  Medical Research Council, Clinical Sciences Centre  Hammersmith Hospital  Du Cane Road  London W12 0NN  Tel: 020 8383 8336  Fax: 020 8383 8557  Website: <http://www.csc.mrc.ac.uk/> |
| Project sponsors | The Department of Health |

You are being invited to take part in a research study. Before you take part it is important that you understand why the research is being done and what it will involve. Please take time to read the following information carefully. Talk to others about the study if you wish.

**Part 1** tells you the purpose of this study and what will happen to you if you take part.

**Part 2** gives you more information about the conduct of this study

Please ask us if there is anything that is not clear or if you would like more information. Take time to decide whether or not you wish to take part.

**The Medical Research Council (MRC) & Imperial College, London**

**Part 1- General Information**

**What are genes?**

The human body contains thousands of genes. They are invisible to the naked eye but play a very important role in your well being. **Genes** are your body’s instruction manual - they affect the way you look and also the way your body works, so your health depends on your genes as well as on your diet and lifestyle.

Despite the fact that the body contains so many different genes, the majority of them are common to all human beings. However, there are some differences between people and this means that, apart from identical twins, everyone has what is known as a different ***genotype***. It’s this difference that explains why people have different looks, such as height and hair colour. It can also explain why there are differences in people’s health, for example:

- why some people are more likely to get a particular disease than others
- why diseases progress more easily in some people than others
- why medicines work in some people and not others.

**What is DNA**

We now know that people’s genes are constructed from large molecules called **DNA**. This DNA contains information, represented by a complex sequence of coded sub-units which is effectively a code, similar to Morse-code. Cells are able to ‘read’ these sequences and produce various bi-products according to this code. Throughout the body we have a wide range of specialised cells which perform different functions. The **DNA sequences** which are ‘read’ by different cells can vary, depending on the functions that the cells are designed to perform.

**What is gene expression?**

**Gene expression** refers to a reading and decoding process of DNA sequence information which takes place within living cells. This process involves the controlled synthesis of molecules within the cell to be used in a variety of metabolic processes. There are many thousands of genes in the human genome. Each gene, when expressed, results in the synthesis of an intermediate molecule called **RNA**. RNA performs a similar function to DNA in that it is able to represent information which the cell needs in order to function. RNA molecules are much smaller than DNA since they only need to carry the code for a single gene at a time. In this capacity, RNA facilitates transfer of the information within cells. It is possible to estimate the level of expression for any one gene by measuring the amount of gene-specific RNA within a cell sample.

**What is the purpose of this study?**

We want to collect information about the way that different peoples’ genes respond to various conditions and store it in a computer system. Scientists can use different techniques to examine people’s gene expression. For this project we will be using a technique called ***DNA Microarrays*** which allows for the examination of thousands of your genes in one go.

In addition to the gene expression results obtained from the microarray technique, we would also like to store information about your lifestyle and medical history (for example, what diseases you have had and what medicines were used to treat you).

We intend to store all of this information in a large, secure database (or Data Warehouse). We want make this information available to scientists and doctors throughout the country, to help them with their research.

The precise form of the gene expression tests to be carried out and the additional lifestyle and health records will depend on the clinical trial that has recruited you.

**Why is this information needed?**

Understanding more about different human genes will help doctors and scientists understand more about many diseases and how to treat them.

Using the information in our computer system, scientists can look at people’s “genetic make up” and medical history to look for patterns or “links” in the way that different genes behave and how the body responds to diseases and medicines (e.g. they may find out which genes are linked to the development of a particular disease, making it easier to predict who might be at risk.)

In the future, scientists may also be able to work out which medicines will work best for different people (i.e. for people with different “genetic make ups”). This may help doctors to make sure that individuals get the best medicine for the way their own body works - something called “*Personalised Medicine”.*

Because we are all different, and have different “genetic make ups”, the more information we can get from different people, the better we can understand the human body. It will also give scientists the opportunity to compare their findings with those of other scientists – this helps them to build a much bigger and clearer picture.

This is why we want to make information on the computer system available to other research scientists - to help them in their research.

As more experiments are undertaken, more information will be available for scientists and doctors to make conclusions from their work.

**Why have I been chosen to take part?**

If you have been asked to read this sheet it means that at some stage you have taken part in a clinical trial whose investigators have chosen to work with us. They will be routinely approaching patients who are involved in their trial to ask them if they are prepared to allow their information to be recorded within our computer system.

**Do I have to take part?**

No. It is up to you to decide whether or not to take part. If you do, you will be given this information sheet to keep and be asked to sign a consent form. You are still free to withdraw at any time and without giving a reason. A decision to withdraw at any time, or a decision not to take part, will not affect the standard of care you receive.

**What does this mean for me?**

By agreeing to allow us to store your information your medical treatment will be completely unaffected. Information about your medical history will be combined with the results of microarray analysis and stored within our computer system. Doctors and scientists will then be able to use all of this information to help them understand more about diseases, how they develop and what the best way of treating / preventing them is. These doctors and scientists who refer to this information will not be able to identify you.

Before agreeing to take part in this study you may want to ask a number of questions so you can make an informed decision. We have provided answers to the ones most people ask below, but if there is more that you want to know you should feel free to contact the custodians of the Data Warehouse (see contact details on the first page of this sheet).

**Has this activity been approved by Research Ethics Committee?**

Yes. The custodians of the Data Warehouse have been granted ethical approval by a Multi-centre Research Ethics Committee. This gives them permission to collect patient details from multiple clinical trials. The clinician/scientist who is dealing with your case is acting on our behalf in seeking your consent for transfer of information to our Data Warehouse.

The trial for which you have been recruited has its own local Research Ethics Committee that has given approval for the trial to take place. As part of this process the local Research Ethics Committee has examined this documentation (Patient Information Sheet and Consent Form) and given approval for patient data to be supplied to the Data Warehouse.

***Permission is conditional on you giving us your consent.***

The following points will tell you what is involved and what we are asking you to consent to.

**Part 2 - Answers to specific questions**

| ***1. What information about me will be stored on the computer storage system?*** |
| --- |
| As you go through the clinical trial, some information about your medical condition will be stored. The type of information will be:   - your medical condition - your past medical history - your lifestyle (smoking, drinking, exercise etc) - results of laboratory tests (including gene expression)   The precise form of information will vary, depending on the particular clinical trial that originally recruited you, however this will definitely include details of your gene expression from microarray examination. |

| ***2. Will this information be stored under my name?*** |
| --- |
| No – the information about you will not be stored under your name. Only the doctor and scientists directly involved in your clinical trial will know it is your information. If the information is shared with another scientist to help them in their research, they will not know who the information has come from. This is a process called “link-anonymisation”.  The doctors and scientists who are responsible for your clinical trial will assign a unique code which links all the samples and information collected to you but allows the information to be kept separately and securely. The code allows other researchers to access an anonymous copy of information that has been collected about you.  When they assign this code to your record they will remove all identifiable information from your record (e.g. name, address, GP name). To protect you further they will replace your full date of birth with simply the month and year of your birth. |

| ***3. How will the link-anonymised information be transferred to the data warehouse?*** |
| --- |
| The link-anonymised record will either be transferred to the data warehouse over a secure computer network or, if this is not possible, an encrypted file will be created by the clinical trial personnel and stored on a removable data storage media (i.e. floppy disk, CD-ROM) for transfer to the MRC Microarray Centre for decryption and importation into the data warehouse.  For data transfer on removable media we use the Rijndael cryptographic algorithm which, in 2001, was specified by the National Institute of Standards and Technology (NIST) in Federal Information Processing Standards (FIPS) Publication 197 as the Advanced Encryption Standard (AES). The AES was announced by NIST as an approved encryption technique for use by the U.S. government, private businesses, and individuals. When properly implemented as a key component of an overall security protocol, the AES permits a very high degree of cryptographic security, yet is fast and efficient in operation. |

| ***4. Who is responsible for looking after the computer system and the information stored on it?*** |
| --- |
| The people responsible for the computer system are doctors and scientists working both for the Medical Research Council and Imperial College, London. Their security policy is drawn from the three principles of data security:   1. Confidentiality: ensuring that link-anonymised information is accessible only to authorised people 2. Integrity: safeguarding the accuracy and completeness of the data 3. Availability: Ensuring that authorised users have access to the information system when required   To achieve this they have a dedicated team of software developers, database administrators and support personnel working within a highly secure organisational Wide Area Network (WAN). The database is located behind two firewalls and all data management is mediated by their own middleware software which has been written specifically to support flexible but secure remote access. All access is achieved through password protected, individualised authentication. Data integrity is assured by adoption of rigorous testing, and change management in combination with regular backups and transaction logging. Multiple user access mechanisms are being developed to support web-browser and rich client access to the underlying data warehouse. |

| ***5. How long will you keep my information for?*** |
| --- |
| The intention is to keep the information forever so that, in time, the data warehouse can gradually expand to provide a valuable resource of knowledge for future doctors and scientists. |

| ***6. Can I change my mind? What if I decide I don’t want my information stored in the computer system anymore?*** |
| --- |
| You are free to withdraw at any time and without giving a reason. A decision to withdraw at any time, or a decision not to take part, will not affect the standard of care you receive. You may request for your information to be removed from the data Warehouse either by contacting the clinical trial doctor / scientist or the Custodians of the Data Warehouse (whose contact details appear on the first page of this document). |

| ***7. Will you want more information when the trial is finished?*** |
| --- |
| On completion of your initial trial, the doctors and scientists who recruited you for that trial may wish to collect follow-up information, allowing them to gain further information about how your medical condition has progressed. If this is the case then they will contact you to explain what they which to do and seek your consent for any further action.  Once again, if any information is collected this will be link-anonymised (see 2) by the trial personnel before being sent to us for inclusion in the data warehouse. |

| ***8. If the information I give is looked at, will I know?*** |
| --- |
| No. You will not be informed of who is looking at the information, or when. In addition, the scientists looking at the information you have given will not know that it is your information. |

| ***9. Who will see my Information?*** |
| --- |
| Your link-anonymised information will be made available to other doctors and scientists. However, anyone who wishes to use the information in the computer system will have to make a formal application to do so. Any such application will involve an assessment of both the scientific merit and ethical implications of an applicant’s proposal.  A Scientific Advisory Committee will examine all applicants to determine whether there is sufficient scientific merit in the request to access the data warehouse. Only if this committee is satisfied will the applicant be able to apply for Research Ethics Approval to access the data warehouse. At this stage a Research Ethics Committee will examine the applicant’s proposal in respect of any ethical issues, reaching a judgement on the suitability of providing access to the applicant.  The Custodians of the data warehouse will maintain a log of all activity in respect of access to information contained within the Warehouse and this log will be regularly reviewed. |

| ***10. Will you receive any feedback?*** |
| --- |
| Although the personnel running the clinical trial for which you have been recruited may provide you with their own feedback, it will not be possible for you to receive any feedback from the data warehouse custodians. |

| ***11. Will you make money from the information I give?*** |
| --- |
| No. The information in the computer system is to be used by doctors and scientists for research. If, in the future, we decide to commercialise any aspect of this data warehouse, including either the database technology or the data contained within the data warehouse, this commercialisation will be carried out in conjunction with MRC Technology Ltd., as prescribed in the “MRC Code of Conduct”.  If you have any further questions about the MRC technology transfer and commercialisation you can contact:  Dr John Kelly  Technology Transfer Manager  Medical Research Council Technology 1 - 3 Burtonhole Lane Mill Hill London   NW7 1AD Tel:   + 44  (0) 20 8906 7118 (Direct Line) Fax:  + 44  (0) 20 8906 7201 |

| ***12. What if there is a problem?*** |
| --- |
| In the event that something does go wrong and you are harmed during the research study there are no special compensation arrangements. If you are harmed and this is due to someone’s negligence then you may have grounds for a legal action for compensation against (name of Sponsor Organisation, NHS Trust, Private Clinic) but you may have to pay your legal costs. The normal National Health Service complaints mechanisms will still be available to you (if appropriate).  If any break of confidentiality occurs this is the responsibility of the team who have recruited you for your clinical trial. It is their responsibility to safeguard your medical record and to ensure that all identifying information is removed from your record before it is passed to us for inclusion in the data warehouse. |

| ***13. Who is organising and funding the research?*** |
| --- |
| This research project is coordinated by the Medical Research Council and Imperial College, London. It has been funded by the Department of Health under the New and Emerging Applications of Technology funding stream. |

| ***14. Who has reviewed the study?*** |
| --- |
| This study has been approved by the Eastern Multi-Centre Research Ethics Committee.  Ref: 05/MRE05/69 |

**The team developing this warehouse would like to thank you for your help in its development. The information you give may help to made medical breakthroughs which may help you or help others in the future.**
