# Supplementary material for: MiMiR – an integrated platform for microarray data sharing, mining and analysis
Source: BMC Bioinformatics. 2008 Sep 18;9:379. doi: 10.1186/1471-2105-9-379 (PMC2572073; doi:10.1186/1471-2105-9-379)
Supplement: Additional File 9 — cMiMiR Data Mapping Tool user guide and practical example. [file 1471-2105-9-379-S9.doc]

**Castillo T. et al. ‘cMiMiR: A Framework for Clinical Microarray Data Mining and Analysis’. BMC Medical Genomics, 2008.**

**Prototype Data Mapping Tool: user guide and practical example**

**1. Using the Unified Medical Language System (UMLS) Knowledge Source Service (KSS)**

The prototype Data Mapping Tool uses the Java API available from the Unified Medical Language System (UMLS) to search for clinical terms from SNOMED-CT and other vocabularies. Remote searching of UMLS clinical terms relies upon a mechanism called Remote Method Invocation (RMI) which allows to execute searches on the remote UMLS server hosted in the USA and returns results as XML files. This technique requires prior registration of the client (your own) IP address with the US server to protect against malicious, unsolicited requests from bogus clients.

The registration process to access the UMLS is done via the following link <http://kswebp2.nlm.nih.gov/UMLSKS/servlet/Turbine/template/admin,user,KSS_login.vm> . Follow the link to obtain a UMLS licence number and create a new user account. Once the account has been activated (appropriate notifications are sent by email following online registration) the client IP address would have to be inserted to the user profile (via the ‘Edit Views/Profile’ link).

*Please Note:*

(a) If you are using an internet provider which may allocate IP addresses dynamically, you need to make sure you have a static IP address or that your address at the time you attempt to access the service matches the one registered with the UMLS service. If not you may have to update your user profile at the above UMLS link (you will have to log in with the user name and password you specified during registration).

(b) If you are behind a firewall or using NAT, the IP address must be the external one for your computer. Talk to your system administrator for more information.

(c) This application was written based on the KSS API v.5. Our understanding is that this is now slowly being phased out and some functionality may not be operable in the future. We have noticed that the ability to perform “approximate search”, meaning returning all terms containing or being related to the term being queried, is not operational anymore. Therefore the current implementation uses the “exact match” which is slightly more limiting in that the caller should be more precise in the term they use in their query.

(d) We have experienced intermittent problems with accessing the UMLS services in the past few weeks which is probably due to the ongoing work at the UMLS and are not related to the Data Mapping Tool. The Command Prompt should help to identify problems with connecting to the UMLS server.

**2. Using the stand alone version of the cMiMiR Data Mapping Tool and practical example**

**General**

The Data Mapping Tool has been designed to allow for patient records from external data sources to be imported into the cMiMiR database by creating formal mapping specifications for the description of data sources using standard terminologies.

The following guide describes how a user of the Data Mapping Tool would employ it to create such a mapping specification. Once such a specification has been created, data can be automatically extracted from the data source and inserted into cMiMiR as an XML file. Please note that the Data Mapping Tool available for testing is a stand alone application and access to cMiMiR has therefore been disabled.

**Linking to the Data Source**

For the purpose of the testing of the Tool, an Access database containing random anonymised clinical data is provided as an example of data source (called ‘anonym_pat_data.mdb’). Please note that the example data source contains randomly generated data and is not password-protected. In a real situation we would expect a higher level of protection of the data sources, even though in an anonymised format.

In order for the Data Mapping Tool to be able to locate and automatically connect to the data source, an appropriate link in the form of an *ODBC Data Source* must be set up:

1. Go to the Windows ODBC Data Source Administrator:

Programs 🡪 Control Panel 🡪 Administrative Tools 🡪 ODBC Data Source Administrator


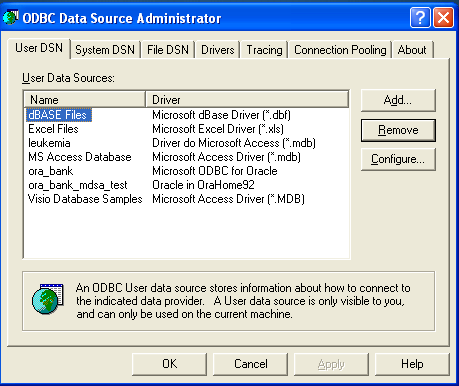


2. Click on “Add” and in the dialog that appears choose the “Microsoft Access Driver” and click “Finish”.


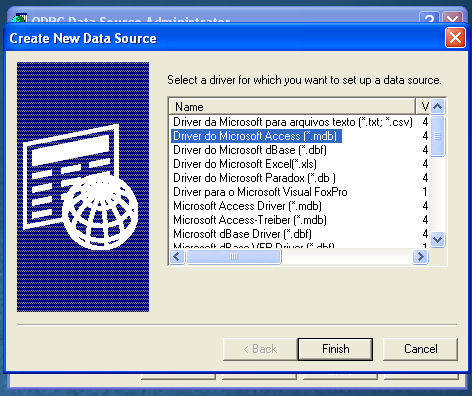


3. In the following dialog click “Select” to point to where you have saved the *anonym_pat_data.mdb* file and enter a name for the data source, “*clinical_db*” for this demonstration. The final dialog should look something like the picture below, although depending on the location of *anonym_pat_data.mdb* on your disk it might slightly differ.


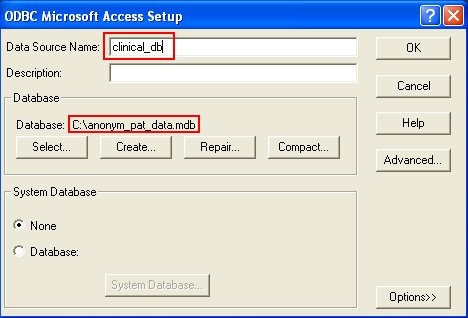


4. Click OK and this should create the ODBC link.

Now you are ready to run the Mapping Tool.

**Prerequisites**

(a) Java version **1.5_06** and above. You can find which is the default java version for your system by executing the following from a DOS command prompt:

java -version

(b) Having registered with UMLS (see above), although this is not a prerequisite for the application to run, it is required in order to programmatically search clinical vocabulary repositories

**Running the cMiMiR Data Mapping Tool – a practical example**

You need to go to the folder where you have downloaded / copied the distribution files and double-click on the file: *run_mapping_tool.bat*

The data mapping tool should start and a screen similar to the one below should appear. You should select File 🡪 New, to create a new mapping specification.


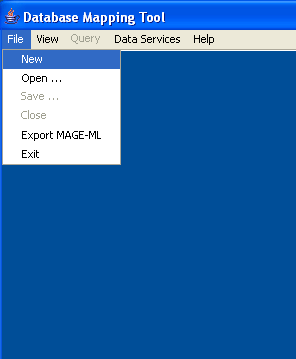


In the following dialog you will have to tell the mapping tool the ODBC link name to the data source. Click on the root node of the tree on the left-hand side of the dialog for the data source details to appear. We have not set up any user name or password so they can be left empty. Insert jdbc:odbc:*clinical_db* and click “Save”. You should see something very similar to the one below:


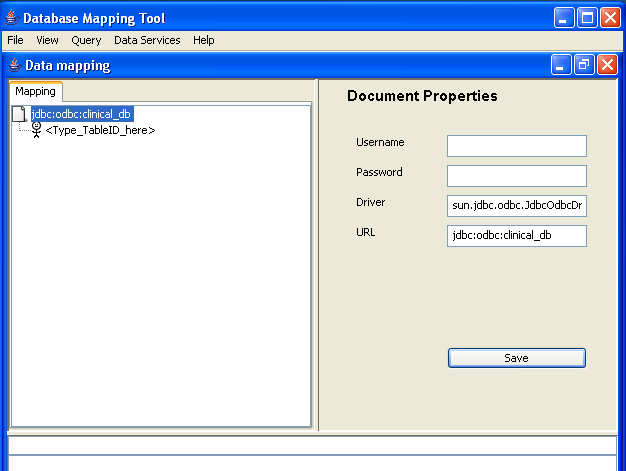


Now the mapping tool knows about the data source and you are ready to create the mapping specification. Note that you could have created the specification manually but the tool can look into the data source and automatically find all available data fields for you, thereby minimising effort, as seen below.

The root patient node is represented by the stick man and is the table with the clinical data we’re interested in creating the mapping specification for.

You should click on the “stick-man” node (root table node) and see the following


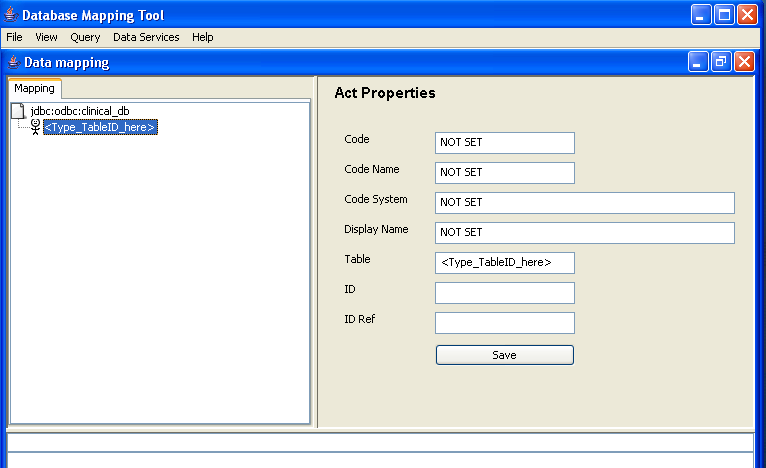


The root Act tag normally points to a patient(C0030705) in UMLS version 2006AD, so the Code, Code Name and Code System could be set but the corresponding table name in the data source still needs to be defined.

Right click on the “stick-man” and choose the table with the clinical data: *tblPatients* in this demo.


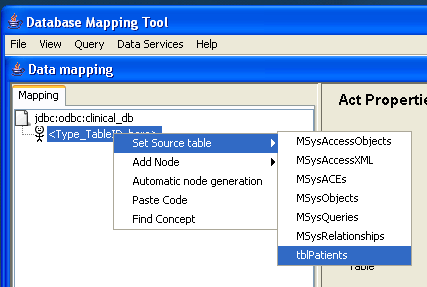


Now the root table (Act) has been defined.


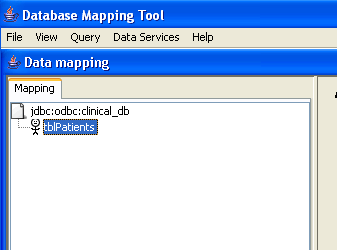


Now the tool has all available information to go into the data source, find all table fields (columns) and insert them automatically for you into the mapping.

Right-click on the tblPatients node and choose “Automatic Node Generation”


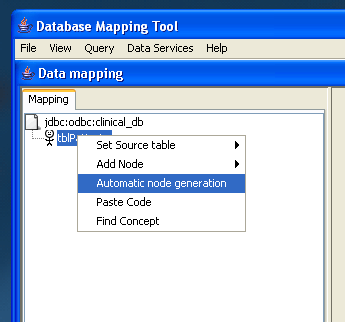


Your screen should look like the following (you may have to expand the tree, click on the + symbol of the root node)


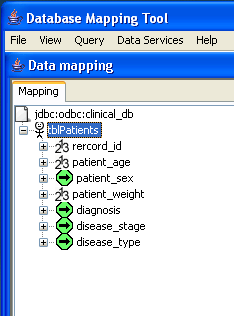


As mentioned above this could have also been accomplished manually via the mapping tool if instead of automatic node generation you manually added each node via the “Add Node” option(s) available (Act, Code, Numeric, Boolean, String, etc)


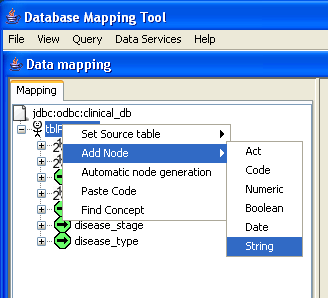


One thing that is required for the (later) automatic data extraction and subsequent saving to MIMIR (not demonstrated here) is the setup of the primary key in the data source. In our case this is the patient record ID.

Right-click on the *record_id* node and choose “Use As Primary Key” and click “Save”


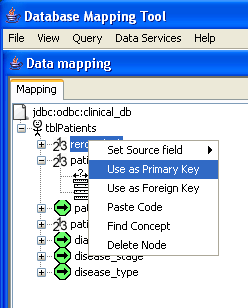


You can now start assigning Code, Code Name / System and Display Name to each of the entries automatically generated. You should click “Save” to save your changes in memory. To save to a local file (saving to MIMIR is not demonstrated since it requires remote access to the MIMIR repository) see below.

For example, to assign values to the node named *patient_sex*


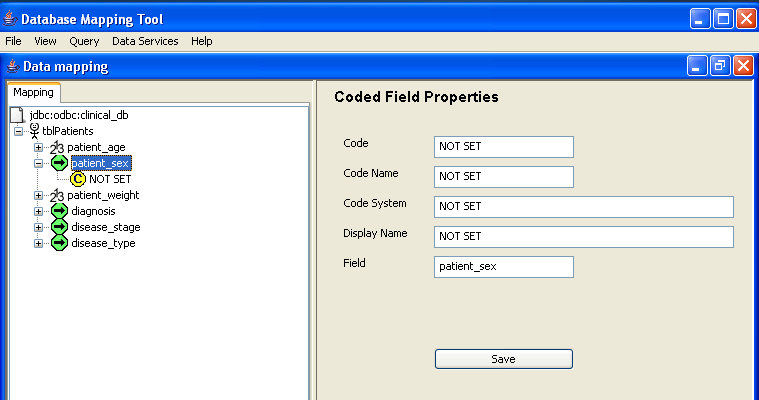


You can either manually enter it or look it up via the search functionality provided via the UMLS KSS.

Enter “Sex” in the dialog and hit the “Search” button on the bottom. The lookup might take 1-2 seconds and you should get back something that should look similar to the following


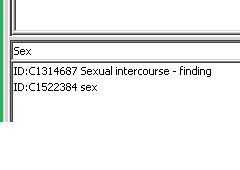


By double-clicking on the appropriate entry, the relevant information will be filled in the corresponding Coded Value Properties boxes. Once again, click “Save” to save changes in memory.


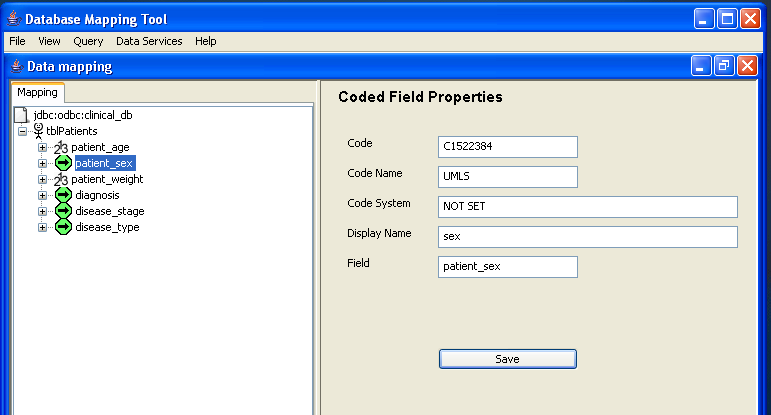


In order to set the different possible values for this entry, you need to expand the node and work in a similar fashion for each entry. In this demonstration, the two possible entries are Male (represented as “M” in the database) and Female (represented as “F” in the database). Therefore two values would need to be added (one exists already by default – but it’s not set) below the patient_sex node. Either via UMLS lookup or manually the value can be set as below.

Note that once you have clicked “Save”, the node assumes the “Value” from the Value field of the Coded Value Properties (while initially it’s “NOT SET”).


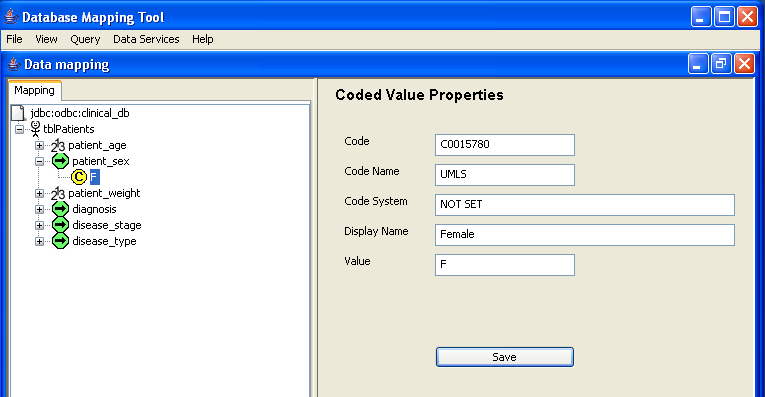


When you are ready to save your changes to a file, click from the menu: File 🡪 Save and an appropriate Save dialog will appear and prompt you to store the file on your disk. The resulting file is an XML file so you must save it with an *xml* extension.


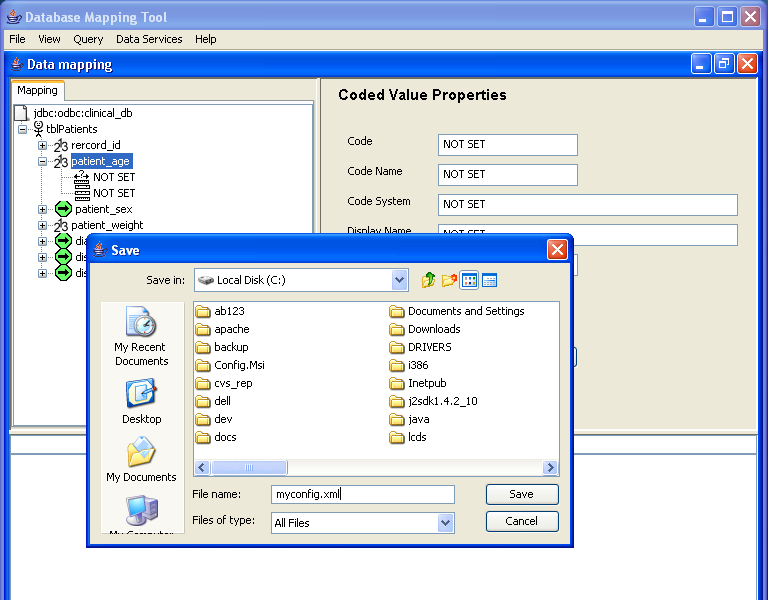


For a complete data mapping specification example file you may want to open *leukemia.xml*, either in a text / xml editor or in the Data Mapping Tool, via File🡪Open and then select the *leukemia.xml* file.
